# Assessment of depression and pain in the general population: a scoping review

**DOI:** 10.1186/s12888-026-08510-2

**Published:** 2026-08-11

**Authors:** Kira Schubert, Kathrin Wandscher, Kathrin Jobski, Sonja Sydekum, Carsten Bantel, Falk Hoffmann

**Affiliations:** 1 Fakultät VI Medizin und Gesundheitswissenschaften, Department für Versorgungsforschung, Carl von Ossietzky Universität, Ammerländer Heerstraße 114-118, 26129 Oldenburg, Germany; 2 Department of Health Services Research, School of Medicine and Health Sciences, Carl von Ossietzky Universität Oldenburg, Oldenburg, Germany; 3 University Department of Anesthesiology, Critical Care, Emergency and Pain Medicine, Klinikum Oldenburg, Oldenburg, Germany

**Keywords:** Depression, Pain, Comorbid, Measurement

## Abstract

**Background:**

Depression and pain, which often overlap, are highly prevalent in the general population. However, the available evidence synthesis on their comorbidity is limited to specific patients. We aimed to give an overview on the assessment of co-occurring depression and pain in the general population focusing on measurements for both disorders.

**Methods:**

This scoping review was registered (10.17605/OSF.IO/DCE3Q*)* and received no funding. MEDLINE (via PubMed), PsycINFO (via EBSCOhost) and EMBASE (via Elsevier) were searched from inception to 10th of December 2024. Cross-sectional and cohort studies with ≥ 100 participants ≥ 14 years, presenting prevalences and/or effect estimates for both depression and pain were included. Data analysis was performed following the principles of Arksey and O´Malley.

**Results:**

We screened 16.916 records and included 60 studies reported in 61 articles, published between 2001 and 2024, of which 42 were cross-sectional and 18 were cohort-studies. Depression was assessed with 19 different measurements, including 17 instruments (most often CES-D, GDS and PHQ), and 2 question based measures. Pain was assessed with 20 different measurements, including 17 instruments (most often VAS, BPI and FPS-R), and 3 question-based measures. The reported comorbid prevalence varied: pain by depression 6.8–87% (median: 50.2%; *n* = 12) and depression by pain 11-84.5% (median: 46.5%; *n* = 14).

**Conclusion:**

Depression and pain show high mutual comorbidity in the general population (about 50%), but measurements in the literature were heterogenous in terms of versions, cut-offs, definitions or assessed characteristics. In patients with either depression or pain, we propose a standardized initial assessment also considering the multidimensionality of pain.

**Clinical trial number:**

Not applicable.

**Supplementary Information:**

The online version contains supplementary material available at 10.1186/s12888-026-08510-2.

## Introduction

Depression and pain are among the most common diseases in the general population, resulting in an immense individual and societal burden [1, 2]. Especially when comorbid, they increase symptom severity, worsen clinical outcomes and influence the course of diagnostic and therapeutic procedures [2–4]. Although both conditions often overlap, they are rarely considered together, especially in the general population [5, 6].

This high relevance is emphasized by the large number of existing studies examining depression and pain in specific populations [7]. Most of these studies examine patients with previously diagnosed depression and investigate associations with pain [8]. Other studies focus on patients with chronic painful conditions such as rheumatoid arthritis [9] or osteoarthritis [10] and possible comorbid depression. Several studies have also looked at the co-occurrence of depression and pain in the general population and the evaluation of possible comorbid relationships. However, the prevalence of co-occurring depression and pain varies widely according to the assessment instrument used [11, 12].

The relationship between co-morbid depression and pain has also been investigated by systematic reviews, but these remain limited to assessing both disorders in specific populations or settings [13–15]. Even existing scoping reviews with a focus on assessment instruments are limited to certain populations such as people with shoulder pain or low back pain [16, 17]. This leads to a restricted transferability of the results on the general population, but there is currently no evidence synthesis on assessing co-occurring depression and pain in the general population.

Therefore, this scoping review aims to provide a comprehensive overview on the existing studies examining co-occurring depression and pain in the general population with a focus on the measurements used for the assessment of both disorders. Furthermore, we will map the reported outcomes to supplement the overview.

## Materials and methods

We followed the methodological framework for scoping reviews described by the Joanna Briggs Institute (JBI) [18], and reported according to the extension of the Preferred Reporting Items for Systematic Reviews and Meta-Analysis statement for scoping reviews (PRISMA-ScR) [19]. The protocol was registered in the Open Science Framework (10.17605/OSF.IO/DCE3Q*).*

### Inclusion criteria

We included published cross-sectional or cohort studies (prospective and retrospective) with a minimum sample size of 100 participants to ensure adequate precision of proportion estimates and reduce the risk of selection bias [20]. We excluded case-control studies and other study types (e.g. review articles, interventional or qualitative studies), editorials, PhD theses and conference abstracts as well as gray literature. There were no restrictions on study length, date or publication language.

We used the PCC (population, concept, context) approach for scoping reviews recommended by the JBI to define further eligibility criteria [18].

#### Population

We included studies focusing on adults from the general population [21] irrespective of lower age limit (e.g. ≥14 years, ≥ 40 years or ≥ 60 years), sex (only women, men or both), geographical location (e.g. nationwide, region or city) or ethnicity (e.g. Asian Americans or Hispanics). Only studies comprising both individuals with/without depression and with/without pain were included. Studies limited to children or adolescents (e.g. including only participants aged ≤ 19 years) [22], or occupational groups (e.g. veterans or flight crews) were excluded, as well as studies limited to certain physical or mental disorders (e.g. rheumatoid arthritis, migraine, anxiety or dementia), medical interventions or treatments (e.g. operations, acupuncture or physiotherapy).

#### Concept

We included studies assessing any depression [23] and pain [24], both defined and measured by the study author’s criteria. To be included, studies had to present results as frequencies (e.g. prevalence) and/or effect estimates (e.g. odds ratios or risk ratios) for pain by depression or depression by pain. We excluded studies assessing depression or pain only by specific medications. Studies assessing or reporting only specific pain sites, types or mood disorders were also excluded. Studies using combined scales for depression (e.g. anxiety/depression with the Kessler Psychological Distress Scale) or for pain (e.g. pain/discomfort with the EQ-5D [European Quality of Life 5 Dimensions]) or reporting only the total score rather than the subscale score (e.g. subscales for depression and anxiety in the HADS [Hospital Anxiety and Depression Scale]) were also excluded.

#### Context

Studies from any country worldwide including national, regional or community sample frames that report measuring depression and pain were included. We excluded studies solely restricted to specific settings with institutionalized individuals, workplaces or educational institutions because their sample composition does not represent the general population.

### Search strategy and study selection

We searched the electronic databases MEDLINE (via PubMed), PsycINFO (via EBSCOhost) and EMBASE (via Elsevier) from inception to December 10th, 2024. Search strategies used in previous reviews for depression [25], pain [26], comorbidity [27, 28], and general population [29] were adapted (Table s1). To supplement database searches, we performed one forward (citing) and backward (cited) citation analysis of all included studies using Web of Science Core Collections on eligible references on February 20th, 2025. For studies not indexed in Web of Science Core Collections, no citation analysis was performed either manually or in other citation indexes.

All search results were imported into an Endnote library (Version 21, Clarivate, Philadelphia, PA, USA) and, after removing all duplicates, to Rayyan QCRI [30]. The screening process was piloted on 100 title/abstracts by KJ, KW, SSy, and KS. After finding consensus, the remaining title/abstracts were screened by KS and one other reviewer (KJ, KW, SSy) independently. The full texts of all articles meeting the inclusion criteria were also assessed independently by KS and another reviewer (KJ, KW). Any disagreements were resolved by discussion or by a third reviewer (KJ, KW, FH). Full texts written in other languages than English or German were translated into German by persons fluent in the original language and German.

### Data extraction

Characteristics of the studies (author, year of publication, country, study design, year of data and primary objective with first reporting), the participants (sample size, gender, age), setting (geographical location, sampling frame), assessment (instruments for assessing depression and pain, frequency of use, description as reported in the included studies), and findings (results as frequencies and/or effect estimates for pain by depression or depression by pain as single values, total values or follow-up values) were extracted by one reviewer (KS) and verified by a second reviewer (SSy). All data were extracted and transferred into the figures and tables presented in the article. The extraction process was previously piloted on five articles before. For the structured extraction of the pain characteristics, we developed an operationalization for duration/frequency, intensity and presence. Another operationalization was developed for the reported outcomes regarding the prevalence of depression, pain, pain by depression and depression by pain. The following (unambiguous) values were extracted and used for calculations: if a single value was reported, that value was preferred; if there were individual values (e.g. for different severities) and a total value, the total value was preferred; if there were multiple values due to different time points (baseline vs. follow-up), the follow-up value was preferred; otherwise, if there were multiple values for a prevalence and no total value, no value was preferred. Discrepancies were resolved by discussion or by a third reviewer (KW, KJ, FH).

### Quality assessment

As scoping reviews typically do not include an assessment of bias [18], none was performed.

### Deviation from the protocol

All content and procedures described in the protocol were generally followed, but an adjustment was made for the citation analysis due to limited personnel resources. We only performed one iteration of the forward and backward citation analysis, since this search already yielded over 4,000 articles.

### Data synthesis

We analyzed the data according to the methodological framework for scoping reviews by Arksey and O’Malley [31]. As a first step, we descriptively analyzed baseline characteristics, including study and participant characteristics, primary research objective and the included studies’ empirical and analytical methods. Secondly, we analyzed the assessment instruments for depression and pain used by each study. During this process, we summarized and visualized the frequency in which they were used and identified overlaps and differences in format. In the final step, we explored the presented outcomes on depression, pain and comorbid frequencies or effect estimates as presented by the studies and calculated medians. Multiple articles of the same study were linked. The results were synthesized by one reviewer (KS) and discussed with another reviewer (SSy).

## Results

### Study selection

**Fig. 1:**
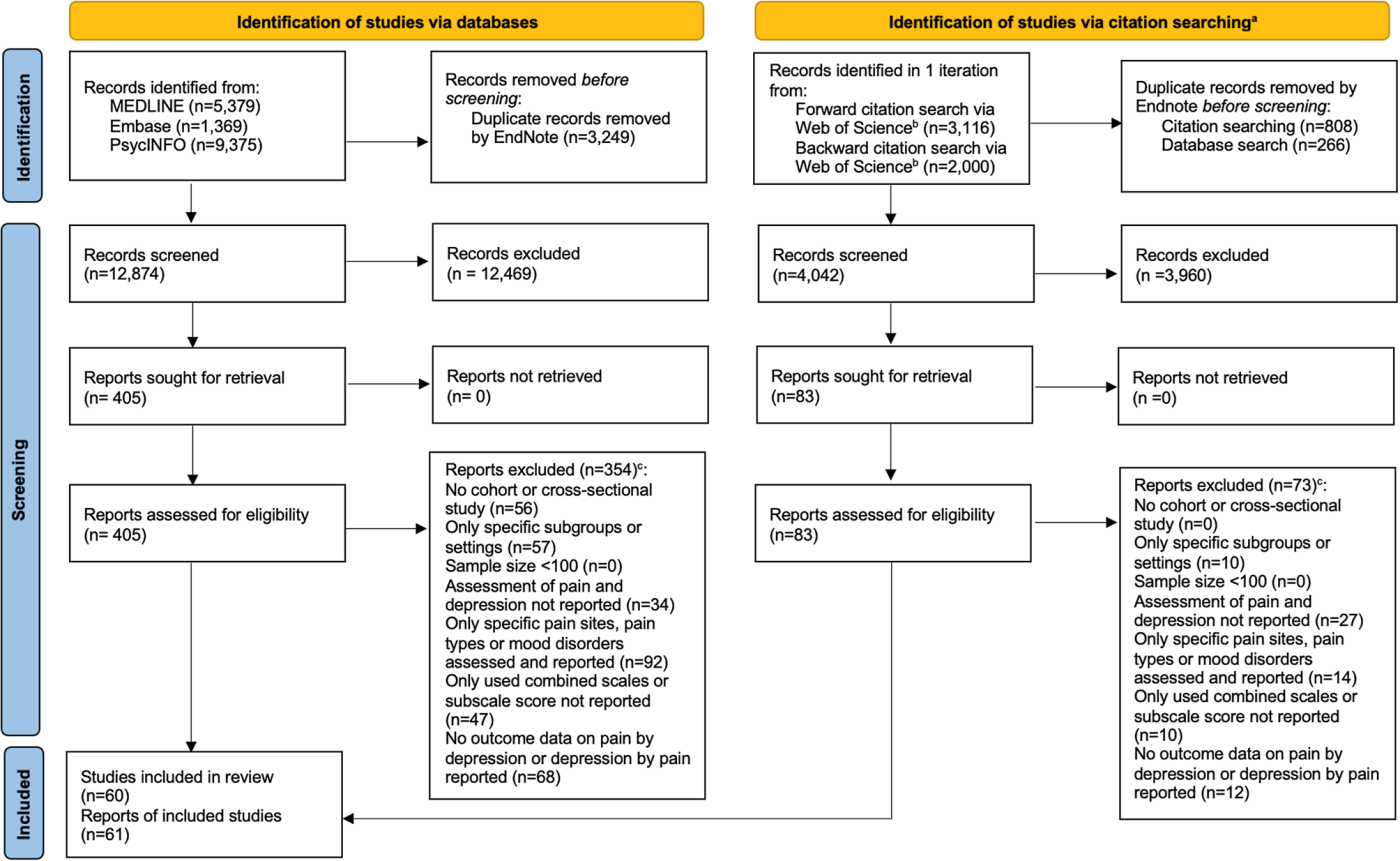
PRISMA flow-chart. ^a^Citation searching was conducted and reported following the TARCiS Statement [93]. ^b^Two studies were excluded from the citation searching as they were not indexed in Web of Science Core Collection [32, 33]. ^c^The assignment of the reasons for exclusion was done hierarchically in the order presented

The database search identified 12,874 records (Fig. 1). After title/abstract screening, 405 full texts were read and 51 articles reporting on 50 studies were included. The citation search identified 4,042 articles, of which 10 were included. Two articles were not indexed in Web of Science Core Collections and were therefore not included in the citation search [32, 33]. Overall, 60 studies reported in 61 articles were included [32–92]. Most articles were written in English (*n* = 58), two in Spanish and one in German. Supplemental Material (Table s2) shows all excluded records after full-text screening with reasons.

### Study and participants characteristics

Details of the included studies are shown in Table 1. 13 studies were from the United States of America, 7 from China, 4 from Germany, 3 from Spain and England, 2 each from Australia, Canada, Chile, Mexico, the Netherlands, 13 countries with one study each, and 6 studies from multiple countries with one global study. 42 studies were cross-sectional studies and 18 were prospective cohort studies. The year of data ranged from 1992 to 2021. In 8 studies, the year of data was unclear. 57 studies reported their primary objective in the title/abstract, focusing on pain and depression (*n* = 22) or only on pain (*n* = 22) or depression (*n* = 13). 2 studies did not report a primary objective.

The sample size ranged from 236 to 91,912 participants. The minimum age criteria for enrollment varied, with 21 studies having a lower age limit between 14 and 35 years, while the remaining required participants to be over 45 years (*n* = 18), or over 60 years (*n* = 20). The mean age of participants ranged from 35.2 to 80 years (*n* = 30) and the proportion of females from 47.5 to 100% (*n* = 43).

**Table 1:** Characteristics of the included studies

Authors; (year)	Country	Study design; year of data	Primary objective (first reporting of the objective)	Sampling frame; geographical location	Sample size
Atlantis et al. (2011) [36]	Australia	Prospective cohort study; 1994–2006	N/R	Aged ≥ 65 years; Melbourne	*n* = 1,000
Wong et al. (2020) [78]	Australia	Cross-sectional study; 2011–2013	Pain (ti/ab)	Aged ≥ 45 years; Kimberly region (KHAP)	*n* = 236
Costa Cabral et al. (2014) [90]	Brazil	Cross-sectional study; 2011–2012	Pain (ti/ab)	Aged ≥ 15 years; São Paulo City	*n* = 826
Shega et al. (2013) [70]	Canada	Prospective cohort study; 1996	Pain (ti/ab)	Aged ≥ 65 years; Canadia (CSHA)	*n* = 4,694
Tripp et al. (2006) [33]	Canada	Cross-sectional study; 2004	Pain (ti/ab)	Aged ≥ 18 years; Ontario	*n* = 1,067
Del pilar et al. (2024) [42]	Chile	Cross-sectional study; 2009–2010	Pain and depression (ti/ab)	Aged ≥ 60 years; Chile (EN-DPM)	*n* = 4,172
Moreno et al. (2022) [85]	Chile	Cross-sectional study; N/R	Depression (ti/ab)	Aged ≥ 70 years; Chile	*n* = 796
Chi et al. (2004) [40]	China	Cross-sectional study; 2000	Depression (ti/ab)	Aged ≥ 60 years; Hong Kong	*n* = 917
Chen et al. (2012) [39]	China	Cross-sectional-study; 2010	Pain and depression (ti/ab)	Aged ≥ 16 years; Beijing	*n* = 2,469
Gong et al. (2017) [47]	China	Cross-sectional study; N/R	Depression (ti/ab)	Aged ≥ 60 years; Dangtu county	*n* = 3,182
Liu et al. (2018) [55]	China	Cross-sectional study; 2010–2011	Pain (ti/ab)	Aged ≥ 19 years; Liuyang region	*n* = 2,052
Ma et al. (2021) [84]	China	Cross-sectional study; 2015	Depression (ti/ab)	Aged ≥ 45 years; China (CHARLS)	*n* = 15,213
Si et al. (2019) [88]	China	Cross-sectional study; 2016	Pain (ti/ab)	Aged ≥ 60 years; Jinan	*n* = 1,219
Tian et al. (2017) [89]	China	Cross-sectional study; 2015–2016	Pain and depression (ti/ab)	Aged ≥ 60 years; Jinan	*n* = 1,788
Borda et al. (2016) [32]	Colombia	Cross-sectional study; 2012	Pain (ti/ab)	Aged ≥ 60 years; Bogotá (SABE-Bogotá)	*n* = 2,000
Arola et al. (2010) [35]	England	Prospective cohort study; 2002–2005	Pain and depression (ti/ab)	Aged ≥ 51 years; North Staffordshire (NorStOProject)	*n* = 4,234
Chou (2006) [41]	England	Prospective cohort study; 2002–2003 and 2004–2005	Pain and depression (ti/ab)	Aged ≥ 65 years; England (ELSA 1 and 2)	*n* = 3,741
Westoby et al. (2008) [77]	England	Cross-sectional study; 2002–2005	Pain (ti/ab)	Aged ≥ 50 years; North Staffordshire (NorStOProject)	*n* = 7,878
Ahrend et al. (2024) [34]	Germany	Cross-sectional study; 2019–2020	Pain and depression (ti/ab)	Aged ≥ 18 years; Germany	*n* = 22,708
Bauer et al. (2016) [37]	Germany	Prospective cohort study; 2012	Pain and depression (f/t)	All born in/before 1943; Southern Germany (KORA-Age)	*n* = 724
Denkinger et al. (2014) [43]	Germany	Cross-sectional study; 2009–2010	Pain and depression (ti/ab)	Aged ≥ 65 years; Ulm (ActiFE-Ulm)	*n* = 1,130
Häuser et al. (2013) [91]	Germany	Cross-sectional study; 2012	Pain (ti/ab)	Aged ≥ 14 years; Germany	*n* = 2,515
Pengpid & Peltzer (2021) [64]	India	Cross-sectional study; 2017–2018	Depression (ti/ab)	Aged ≥ 45 years; India (LASI 1)	*n* = 72,262
Regan et al. (2013) [65]	Ireland	Cross-sectional study; 2009–2011	Depression (ti/ab)	Aged ≥ 50 years; Ireland (TILDA 1)	*n* = 8,032
Goral et al. (2010) [48]	Israel	Cross-sectional study; 2003–2004	Pain (ti/ab)	Aged ≥ 21 years; Israel (INHS)	*n* = 4,855
Yunus et al. (2018) [79]	Malaysia	Prospective cohort study; 2013–2016	Pain (ti/ab)	Aged ≥ 60 years; Kuala Pilah	*n* = 1,927
Garcia-Perez et al. (2022) [46]	Mexico	Cross-sectional study; 2018	Depression (ti/ab)	Aged ≥ 50 years; Mexico (MHAS)	*n* = 14,230
Gutierrez et al. (2022) [82]	Mexico	Prospective cohort study; 2012–2015	Pain and depression (ti/ab)	Aged ≥ 60 years; Mexico (MHAS)	*n* = 7,237
Geerlings et al. (2001) [81]	Netherlands	Prospective cohort study; N/R	Pain and depression (ti/ab)	Aged 55–85 years; Netherlands (LASA)	*n* = 652
Hilderink et al. (2012) [51]	Netherlands	Prospective cohort study; 1992–2004	Pain and depression (ti/ab)	Aged 55–85 years; Netherlands (LASA)	*n* = 2,028
Gureje et al. (2009) [49]	Nigeria	Cross-sectional study; 2002–2003	Depression (ti/ab)	Aged ≥ 18 years; 21 of 36 Nigerian states	*n* = 6,752
Satghare et al. (2016) [68]	Singapore	Cross-sectional study; N/R	Pain (ti/ab)	Aged ≥ 60 years; Singapore (WiSE)	*n* = 2,565
Zager et al. (2023) [80]	Slovenia	Cross-sectional study; 2019	N/R	Aged ≥ 15 years; Slovenia (EHIS)	*n* = 9,900
Dueñas et al. (2024) [44]	Spain	Cross-sectional study; 2022	Pain (ti/ab)	Aged 18–85 years; Spain (Pain Barometer)	*n* = 7,058
Rivera et al. (2019) [66]	Spain	Cross-sectional study; 2013–2014	Depression (ti/ab)	Aged 18–75 years; Andalusia (PISMA-ep)	*n* = 4,507
Sendra-Gutierrez et al. (2017) [69]	Spain	Cross-sectional study; 2014	Depression (ti/ab)	Aged ≥ 65 years; Spain (EHS Spain)	*n* = 6,520
Thomtén et al. (2011) [74]	Sweden	Prospective cohort study; N/R	Pain (ti/ab)	Aged 18–64 years; Stockholm	*n* = 2,300
Rouch et al. (2023) [67]	Switzerland	Prospective cohort study; 2003–2021	Pain and depression (ti/ab)	Aged 35–75 years; Lausanne (CoLaus/PsyCoLaus)	*n* = 2,578
Ko et al. (2018) [54]	Taiwan	Cross-sectional study; 2011	Pain and depression (ti/ab)	Aged ≥ 55 years; Taiwan (HALST)	*n* = 2,315
Erbaydar & Çilingiroğlu (2010) [92]	Turkey	Cross-sectional study; N/R	Pain and depression (ti/ab)	Aged 18–64 years; Ankara	*n* = 259
Bollinger et al. (2020) [38]	USA	Cross-sectional study; 2014	Pain (ti/ab)	Aged ≥ 18 years; Hawaii and Pacific Islands (NHPI-Survey)	*n* = 1,334
Emptage et al. (2005) [45]	USA	Prospective cohort study; 1994	Pain and depression (ti/ab)	All born between 1931 and 1941; USA (HRS)	*n* = 8,280
Janevic et al. (2017) [53]	USA	Cross-sectional study; 2010	Pain (ti/ab)	Aged ≥ 51 years; American (HRS)	*n* = 1,925
McCarthy et al. (2009) [56]	USA	Cross-sectional study; 2004–2007	Pain (ti/ab)	Aged ≥ 70 years; New York City (EAS)	*n* = 840
Miller & Cano (2009) [57]	USA	Cross-sectional study; 2006–2007	Pain and depression (ti/ab)	Aged ≥ 18 years; State of Michigan	*n* = 1,179
Mullins et al. (2023) [58]	USA	Cross-sectional study; 2019	Pain and depression (ti/ab)	Aged ≥ 18 years; USA (NHIS)	*n* = 31,977
Ohayon & Schatzberg (2010) [62]	USA	Cross-sectional study; 2003–2004	Pain and depression (ti/ab)	Aged ≥ 18 years; State of California	*n* = 3,243
Patel et al. (2013) [63]	USA	Cross-sectional study; 2011	Pain (ti/ab)	Aged ≥ 65 years; USA (NHATS)	*n* = 7,601
Shega et al. (2014) [71]	USA	Prospective cohort study; 2005–2011	Pain (ti/ab)	Aged 57–85 years; Chicago (NSHAP)	*n* = 2,799
Shi et al. (2010) [72]	USA	Prospective cohort study; 1992–2006	Pain (ti/ab)	Aged ≥ 51 years; USA (HRS)	*n* = 18,439
Steffens et al. (2009) [73]	USA	Cross-sectional study; 2001–2003	Depression (ti/ab)	Aged ≥ 71 years; USA (ADAMS from HRS)	*n* = 856
Tian et al. (2005) [75]	USA	Cross-sectional study; 1996	Pain and Depression (ti/ab)	Aged 55–65 years; USA (HRS 3)	*n* = 7,350
Uebelacker et al. (2013) [76]	USA	Prospective cohort study; 1997–2001	Depression (ti/ab)	Aged 50–79 years; USA (WHI)	*n* = 91,912
Qiu et al. (2022) [86]	China & England	Prospective cohort study; 2011 and 2013–2015 (CHARLS); 2002/2003 and 2004/2005–2016/2017 (ELSA)	Pain and depression (ti/ab)	Aged ≥ 45 years; China (CHARLS 1–3); Aged ≥ 50 years; England (ELSA 1–8)	*n* = 9,566 (CHARLS); *n* = 8,011 (ELSA)
Langley (2011) [83]	Europe	Cross-sectional study; 2008	Pain (ti/ab)	Aged ≥ 18 years; France, Germany, Italy, Spain and UK (NHWS)	*n* = 53,524
Ohayon & Schatzberg (2003) [61] with companion report Ohayon (2004) [60]	Europe	Cross-sectional study; 1994–1999	Pain and depression (ti/ab)	Aged 15–100 years; Germany, Italy, Portugal, Spain and UK	*n* = 18,980
Horackova et al. (2018) [52]	Europe & Israel	Cross-sectional study; 2004–2015	Depression (ti/ab)	Aged ≥ 50 years; Europe and Israel (SHARE)	*n* = 28,796
Ogliari et al. (2023) [59]	Europe & Israel	Prospective cohort-study; 2013 & 2015	Pain and depression (ti/ab)	Aged ≥ 50 years; Europe and Israel (SHARE)	*n* = 28,515
Raggi et al. (2020) [87]	China, Europe & Mexico	Prospective cohort study; N/R	Pain (ti/ab)	Aged N/R; ATHLOS project (CHARLS 1 and 2, COURAGE 1 and 2, HRS 9–11, Health 2000/2001 1 and 2, MHAS 2 and 3, SHARE 4 and 5)	*n* = 13,545
Gureje et al. (2007) [50]	Worldwide	Cross-sectional study; N/R	Pain and depression (ti/ab)	Aged ≥ 18 years, except for Colombia and Mexico (18–65 years), Japan (≥ 20 years) and Israel (≥ 21 years); 17 countries (WMHS)	*n* = 85,088

**Abbreviations**: ActiFE-Ulm: Activity and Function in the Elderly Ulm; ADAMS: Aging, Demographics, and Memory Study; ATHLOS: Aging Trajectories of Health – Longitudinal Opportunities and Synergies; CHARLS: China Health and Retirement Longitudinal Study; CoLaus/PsyCoLaus: Cohorte Lausannoise/Psychological Cohorte Lausannoise; COURAGE: COllaborative Research on AGEing in Europe; CSHA: Canadian Study of Healthy Aging; EAS: Einstein Aging Study; EHIS: European Health Interview Survey; EHS: European Health Survey; ELSA: English Longitudinal Study of Ageing; EN-DPM: Estudio National de Dependencia en las Personas Mayores; EU: European Union; f/t: full text; HALST: Healthy Aging Longitudinal Study in Taiwan; HRS: Health and Retirement Survey; INHS: Israel National Health Survey; KHAP: Kimberley Healthy Adults Project; KORA-Age: Cooperative Health Research in the Region of Augsburg; LASA: Longitudinal Aging Study Amsterdam; LASI: Longitudinal Ageing Study India; MHAS: Mexican Health and Aging Study; NHATS: National Health and Aging Trends Survey; NHIS: National Health Interview Survey; NHPI: Native Hawaiian and Pacific Islander Survey; NHWS: National Health and Wellness Survey; NorStOProject: North Staffordshire Osteoarthritis Project; NSHAP: National Social Life, Health, and Aging Project; N/R: Not reported; PISMA-ep: Plan Integral de Salud Mental de Andalucía; SABE-Bogotá: Salud, Bienestar y Envejecimiento Bogotá; SHARE: Survey of Health Aging and Retirement in Europe; ti/ab: title/abstract; TILDA: The Irish Longitudinal Study of Ageing; UK: United Kingdom; USA: United States of America; WHI: Women’s Health Initiative; WiSE: Well-being of the Singapore Elderly Study; WMHS: World Mental Health Survey

### Assessment of depression and pain in the general population

#### Assessment of depression

The included studies used 19 different measurements to assess depression, consisting of 17 instruments, and 2 different question-based measures (Table 2). Of these measurements, 18 were self-reported. The most frequently used instrument was the Center for Epidemiologic Studies Depression Scale (CES-D; *n* = 15) followed by the Geriatric Depression Scale (GDS; *n* = 9), the Patient Health Questionnaire (PHQ; *n* = 8), and the Composite International Diagnostic Interview (CIDI; *n* = 6), the Hospital Anxiety and Depression Scale (HADS; *n* = 5), the Sleep-EVAL (*n* = 3) and the European Union Developed Scale (EURO-D; *n* = 2). 7 studies described the assessment of depression by a question-based measure, asking 1 question (*n* = 6) or 3 questions (*n* = 1). Additionally, the studies used different versions of the instruments regarding the number of items. This applied to the CES-D (6 versions), the PHQ (5 versions), the CIDI (4 versions), the GDS (2 versions), the HADS (2 versions) and the Sleep-EVAL (2 versions). 2 studies assessed depression by combining 2 measurements, the Composite International Diagnostic Interview Short Form (CIDI-SF) with the Neuropsychiatric Interview (NPI), and the CES-D with the CIDI-SF. Another 2 studies used the GDS-15 in combination with 1 close-ended question to assess depression. 6 instruments were mainly used in populations ≥ 60 years: the GDS (*n* = 9), the HADS (*n* = 3), the Geriatric Mental State Automated Geriatric Examination for Computer Assisted Taxonomy (GMS-AGECAT; *n* = 1), the Mental Health Inventory 5 (MHI-5; *n* = 1), the NPI (*n* = 1) and the Psychogeriatric Assessment Scale (PAS; *n* = 1). The CIDI was used both in populations over and under 60 years.

27 studies reported a duration of depressive symptoms (Table s3). Of those, 11 studies reported a duration of depressive symptoms over the last week, 10 over 2 weeks, 4 over 12 months and 2 studies assessed the symptoms over the last month. 33 studies did not report the assessed duration of depressive symptoms.

**Table 2:** Assessment of depression and most frequently used tools

Measurement	Frequency used in studies	Items
**Instruments**		
CES-D [41, 45, 46, 51, 53, 54, 65, 71, 72, 75, 76, 81, 82, 84, 86]	15	6 items (*n* = 1);(76) 8 items (*n* = 6);(41, 45, 53, 72, 75, 86) 9 items (*n* = 2);(46, 82) 10 items (*n* = 2);(84, 86) 11 items (*n* = 1);(71) 20 items (*n* = 4)(51, 54, 65, 81)
GDS [32, 37, 40, 42, 47, 79, 85, 88, 89]	9	15 items (*n* = 7);(32, 37, 40, 42, 47, 79, 85) 5 items (*n* = 2)(88, 89)
PHQ [33, 34, 55, 58, 63, 69, 80, 91]	8	2 items (PHQ-2, *n* = 1);(63) 4 items (PHQ-4, *n* = 1);(91) 8 items (PHQ-8, *n* = 3);(34, 58, 80) 9 items (PHQ-9, *n* = 2);(33, 55) Number of items N/R (*n* = 1)(69)
CIDI [39, 49, 50, 64, 73, 75]	6	9 items (CIDI 3.0, *n* = 1);(39) 9 items (CIDI-SF, *n* = 1);(64) 8 items (CIDI-SF, *n* = 1);(73) Number of items N/R (CIDI-SF, *n* = 1; CIDI 3.0 *n* = 1; CIDI-WMH *n* = 1)(49, 50, 75)
HADS [35, 43, 44, 77, 90]	5	14 items (HADS-A and HADS-D, *n* = 4);(35, 44, 77, 90) 7 items (HADS-D, *n* = 1)(43)
Sleep-EVAL [60–62]	3	36 items (*n* = 1);(60) 48 items (*n* = 1);(62) Number of items N/R (*n* = 1)(61)
EURO-D [52, 59]	2	12 items
BDI [92]	1	Number of items N/R
DIGS [67]	1	Number of items N/R
GHQ-12 [74]	1	12 items
GMS-AGECAT [68]	1	Number of items N/R
KICA-dep [78]	1	Number of items N/R
MHI-5 [70]	1	5 items
MINI [66]	1	2 items per disorder
NPI [73]	1	Number of items N/R
PAS [36]	1	Number of items N/R
SCID [57]	1	9 items
**Question-based measures**		
One question [48, 56, 83, 85, 87]	5	1 item (diagnosis of depression, *n* = 3);(48, 56, 85) 1 item (self-report of depression, *n* = 2)(83, 87)
Three questions [38]	1	3 items (presence, medication and intensity of symptoms)

**Abbreviations**: BDI: Becks Depression Inventory; CIDI: Composite International Diagnostic Interview; CES-D: Center for Epidemiologic Studies Depression Scale; DIGS: Diagnostic Interview for Genetic Studies; GHQ-12: General Health Questionnaire 12; GMS-AGECAT: Geriatric Mental State Automated Geriatric Examination for Computer Assisted Taxonomy; HADS: Hospital Anxiety and Depression Scale; PHQ: Patient health questionnaire; MHI-5: Mental Health Inventory-5; MINI: Mini International Neuropsychiatric Interview; NPI: Neuropsychiatric Interview; N/R: Not reported; PAS: Psychogeriatric Assessment Scales; SCID: Structured Clinical Interview for DSM-IV-TR

#### Assessment of pain

Across all included studies, the assessment of pain was heterogenous. There were twenty measurements consisting of seventeen different instruments and three different question-based measurements reported for the assessment of pain (Table 3). The most often used instrument was the Visual Analog Scale (VAS; *n* = 4), followed by the Brief Pain Inventory (BPI; *n* = 2), the Faces Pain Scale Revised (FPS-R; *n* = 2) the Nottingham Health Profile 5 (NHP-5; *n* = 2) and the Verbal Descriptor Scale (VDS; *n* = 2). Forty-six studies assessed pain with a question-based measurement that consisted of either one question (*n* = 17), two questions (*n* = 16) or three (*n* = 6) and more questions (*n* = 6).

Twenty studies did neither assess pain duration/frequency nor intensity (Table s4). Pain duration/frequency was assessed by 9, pain intensity by 28 and pain duration/frequency plus intensity by 10 studies (see operationalization of pain characteristics in Table s7). Most studies did not define the examined pain type (*n* = 35). Among those defining the pain type, 2 assessed acute pain and 24 examined chronic pain with varying definitions. Of those 24 studies assessing chronic pain, 19 specified durations of either three months (*n* = 10), six months (*n* = 4), seven months (*n* = 1) or twelve months (*n* = 4), Overall, 11 studies added further criteria (e.g., regarding intensity or persistence).

**Table 3:** Assessment of pain with frequency, selected characteristics and description

Measurement	Frequency used in studies	Pain characteristics^*,°^	Description as reported by the included studies
**Instruments**			
VAS [43, 55, 74, 92]	4	Intensity	11-point visual scale from “0 = no pain” to “10 = worst imaginable pain/very severe pain” on horizontal line
BPI [67, 78]	2	Duration/frequency, Intensity	3 items duration/frequency, intensity (*n* = 1);(78) 3 items, intensity (*n* = 1)(67)
FPS-R [88, 89]	2	Intensity	Visual scale from 0–10 “mild” to “severe” pain
NHP 5 [51, 81]	2	N/R	5-items
VDS [70, 71]	2	Intensity	5-point scale; (70) 7-point scale(71)
CPG [90]	1	Duration/frequency, intensity	8 items
GCPS [33]	1	Duration/frequency, Intensity	7 items
MOS SF-12 [35]	1	N/R	2 items
NRS [43]	1	Intensity	11-point scale
Pain Disability Index [53]	1	Intensity	Scale from 0–10, most severe pain on average last year
Pain Questionnaire 10/66 [68]	1	Duration/frequency, intensity	3 items
PQ [74]	1	Duration/frequency	4 items
SF-36 [76]	1	N/R	2 items
STOPNEP [67]	1	Duration/frequency	11 items
TPI [56]	1	Duration/frequency, intensity	2 items
U.S. Health Interview Survey [50]	1	N/R	3 items
WPI [91]	1	N/R	1 item
**Question-based measures**			
One question [32, 34, 36, 40, 42, 47, 49, 52, 55, 58, 64, 66, 69, 80, 85, 87, 91]	17	Duration/frequency, intensity, N/R	1 item, duration/frequency (*n* = 2);(36, 58) 1 item intensity (*n* = 7);(32, 34, 42, 47, 69, 80, 85) 1 item, N/R (*n* = 8);(40, 49, 52, 55, 64, 66, 87, 91)
Two questions [37, 38, 41, 44, 45, 59, 63, 65, 71, 73, 75, 77, 79, 82, 86, 88]	16	Duration/frequency, intensity, N/R	2 items, duration/frequency (*n* = 2);(44, 88) 2 items, intensity (*n* = 7);(41, 45, 59, 65, 73, 75, 86) 2 items duration/frequency and intensity (*n* = 1);(38) 2 items, N/R (*n* = 6)(37, 63, 71, 77, 79, 82)
Three or more questions [39, 43, 46, 48, 54, 57, 60–62, 72, 83, 84]	12	Duration/frequency, intensity	3 items, duration/frequency (*n* = 1);(57) 3 items, intensity (*n* = 4);(46, 54, 72, 84) 3 items, N/R (*n* = 1);(48) ≥ 4 items, duration/frequency and intensity (*n* = 3);(43, 62, 83) ≥ 4 items, N/R (*n* = 3)(39, 60, 61)

Abbreviations: BPI: Brief Pain Inventory; CPG: Chronic pain grade; FPS-R: Faces Pain Scale Revised; GCPS: Graded Chronic Pain Status; GPQ: German pain questionnaire; MOS SF-12: Medical Outcome Survey Short Form 12; NHP-5: Nottingham Health Profile 5; NRS: Numerical rating scale; N/A: not applicable; N/R: not reported; PAS: Psychogeriatric Assessment Scale; PQ: Pain Questionnaire; SF-36: Short Form 36; STOPNEP: Study of Prevalence of Neuropathic Pain; TPI: Total Pain Index; VAS: Visual Analog Scale; VDS: Verbal Descriptor Scale; WPI: Widespread pain index

*only duration/frequency and intensity mentioned; N/R equals neither duration/frequency nor intensity assessed; for further information on other assessed characteristics see Table s4

°the operationalization of the pain characteristics is presented in Table s7

#### Assessment of depression and pain

**Fig. 2:**
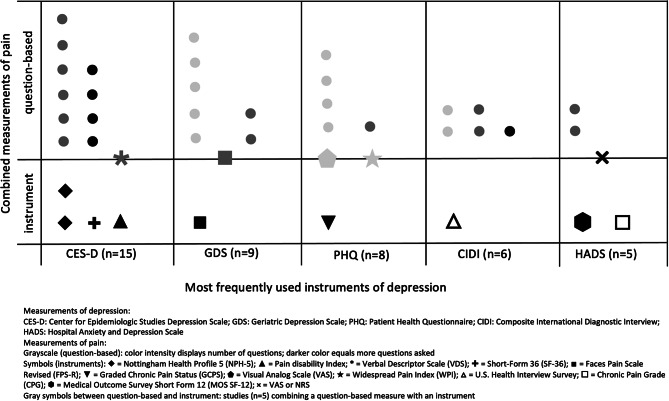
Combination of measurements for the assessment of depression and pain

The combination of measurements for depression and pain was heterogenous across all studies (Table s3 and s4). Although within each individual study, a single method was used to assess depression and pain. Figure 2 visualizes the combination for the five most frequently used instruments of depression with the combined instruments and measures for pain (*n* = 43). Among those, most studies combined an instrument-based assessment of depression with a question-based measure for pain (*n* = 30). For example, the 15 studies using the CES-D for the assessment of depression used question-based measures (*n* = 10), instruments (*n* = 4) or an instrument plus a question-based measure for the assessment of pain (*n* = 1).

The 22 studies focusing on pain and depression (see Table 1) also applied different measures. Most of these studies assessed depression using the CES-D (*n* = 8), HADS (*n* = 2), PHQ-8 (*n* = 2), or Sleep-EVAL (*n* = 2), and pain with question-based measures (*n* = 15), the VAS (*n* = 2), or the NHP-5 (*n* = 2) (Table S5).

### Occurrence of depression and pain

The prevalence of depression ranged from 3.3% to 50.2% (median: 11.6%; *n* = 29) and the prevalence of pain ranged from 8.2% to 79.5% (median: 37.4%; *n* = 29; Table s6). Outcomes for pain by depression were presented by 47 studies, and 56 reported outcomes for depression by pain. Regarding the results for pain by depression, 22 reported prevalences, 25 calculated effect estimates, and 16 studies reported both. The comorbid prevalence for pain by depression ranged from 6.8% to 87% (median: 50.2%; *n* = 12) and effect estimates were mostly odds ratios (*n* = 23). Regarding the results for depression by pain, 28 studies reported prevalences, 28 reported effect estimates, and 18 reported both. The comorbid prevalence for depression by pain ranged from 11% to 84.5% (median: 46.5%; *n* = 14) and effect estimates for depression by pain were mostly odds ratios (*n* = 23). A total of 2 studies assessed both prevalences and effect estimates for pain by depression and depression by pain.

## Discussion

In this scoping review, which included 60 studies reported in 61 articles, we discovered a heterogenous landscape of measurements for depression and pain in the general population. Nineteen different measurements consisting of 17 instruments, and two question-based measures were used in the assessment of depression. Pain was assessed with 17 different measurements, of which most were question-based. We found inconsistencies regarding the assessed pain characteristics, with 20 studies not assessing pain duration/frequency or intensity. The comorbid median prevalence for pain by depression was 50.2% and for depression by pain 46.5%.

One finding in the assessments of depression was the general heterogeneity, although more instruments than question-based measures were used. This might be explained as different instruments have been developed for specific purposes, such as the GDS for older populations [94], whereas others like the HADS [95] are suitable for broader age spans. That, in combination with the use of different versions regarding the number of items applied, could explain the heterogeneity within the assessments of depression. The most prominent example in the included studies is the use of six different versions of the CES-D and four versions of the PHQ. Although it is widely accepted to apply different versions in different settings, such as the use of less items in time-critical situations, this has an impact on the accuracy of the assessment [96–98]. A previous systematic review analyzing the comparability of the PHQ-2 and the PHQ-9 concluded that fewer items are more time-effective while capturing fewer symptoms [99]. If fewer symptoms are assessed, some patients might not be successfully identified [100], and need further assessment with a more in depth questionnaire if appropriate [99]. Another important aspect which needs consideration is the difference between pain-related emotional distress and clinical depression. Pain-related emotional distress is a phenomenon commonly encountered in contact with patients suffering from pain, as especially chronic pain is often accompanied by depressed mood, anger or hopelessness [101]. If generally assessing a patient with pain for depression, the measurement should include the duration of symptoms as required by the International Classification of Diseases Version 10 (ICD-10 [102]), or the Diagnostic and statistical manual of mental disorders Version 5 (DSM-5 [23]). 27 of our included studies reported a duration of depressive symptoms and of those only 10 used the two-week criterion like the ICD-10 or DSM-5 require. The most frequently used assessment for depression in our included studies was the CES-D which, if reported, assessed for depressive symptoms during the last week. Therefore, the available data does not allow the distinction between pain-related emotional distress and clinical depression based on the time-criterion, and the prevalence for depression in patients with pain must be interpreted with caution.

Another finding regarding the assessment of pain was the use of twenty measurements, whereby most studies only used question-based measures. This does not consider the multidimensionality of pain as it is a complex disorder with a plethora of possible underlying causes [103]. The exact assessment of the intensity, duration and frequency influences further diagnostic and therapeutic interventions. Those characteristics were not assessed by most of the question-based measures, since twenty studies did not consider pain duration/frequency or intensity. Underdiagnosis can lead to chronification over time, increasing the individual and societal burden [104, 105]. Grimmer-Sommers et al. compared different properties of pain measurements and concluded that a combination of measurements is necessary to include all dimensions of pain, especially in chronic pain [106]. When reported, most studies assessed chronic pain and even these studies differed greatly in their definitions. That studies included in our review differed in terms of the assessed pain type and characteristics can be explained by their various objectives. About two thirds of the included studies did not measure the co-occurrence pain and depression as a primary aim, which might explain the large differences between measurements and might also result in a suboptimal assessment of both conditions. However, even those studies primarily interested in both depression and pain, measured these conditions differently, which might result in a suboptimal assessment of both conditions. Those with a focus on depression with pain as an influential factor measured fewer characteristics of pain and did mostly not define the pain type compared to those studies with a focus on pain. A previous review underlines that different methodological approaches of studies influence the assessment and recognition of comorbidities of depression [107].

The comorbid prevalence for pain by depression varied between 6.8% and 87% with a median of 50.2%. We found a similar range for depression by pain from 11% to 84.5% with a median of 46.5%. This range for the comorbid prevalence is in line with previous research in specific populations [108, 109]. Two studies reported comorbid prevalences for both pain by depression and depression by pain [41, 81]. Both included older participants and aimed to examine pain and depression with a prospective cohort study design, which makes their samples comparable. Both used the CES-D for the assessment of depression, whereas Chou [41] assessed pain with a question-based measure, and Geerlings et al. [81] assessed pain with the NHP 5. Despite the different approach on the pain assessment, they found increased prevalences for pain by depression (49.3% and 87%) compared to depression by pain (43.0% and 61%). Nonetheless, the comparatively higher comorbid prevalence for pain by depression indicates that many patients with depression seem to suffer from pain. Regarding the assessment of pain in the included studies, most of them using a question-based measure only asked for the general presence of pain, while those studies using instruments such as the BPI gained much more information. Si et al. [88] and Tian et al. [89] chose the FPS-R with respect to the special needs of Chinese elderly, such as lesser formal education, aiming to measure pain in depressed patients. Shega et al. [71] give similar explanations for their choice with the VDS to assess intensity in older populations.

Given the connection between depression and pain, patients presenting for either disorder should be assessed in a uniform way. This need for a standardized assessment especially applies to the need for a structured instrument for pain. Although the FPS-R [110] and the VDS [111] only consider pain intensity, this might be a fitting approach in the initial assessment of pain in general. Previous research has shown that patients reporting pain on intensity scales such as the VDS, VAS or NRS above the cut-off of 5 points should receive some form of treatment [112, 113]. As pain is often comorbid with depression, and sometimes masked by depressive symptoms, an assessment of pain is of great importance [3]. Persistent pain is known to impact the success of therapeutic interventions for depression and an increase in painful symptoms can lead to worsened depressive symptoms [114, 115]. Therefore, in clinical practice the assessment of pain intensity as the initial characteristic is an accessible tool which can also be used to then evaluate the success of treatments. A patient presenting with a high pain intensity can be monitored with scales like the VAS or NRS. An increase or decrease in subjective pain intensity under treatment can be objectified and used for adjustments or an alternative approach on relieving the patient’s pain. Visual (VAS) or numerical (NRS) scales have been proven to be reliable and transformable between each other [116, 117]. This also improves communication between different medical specialties if more than one health professional is involved into the treatment. An initial measurement of the intensity can in the next step be supplemented by other instruments depending on the individual patient’s needs. Older patients for example might have other comorbidities compared to middle-aged patients where other causes of pain are more prevalent [118, 119]. For those populations, an instrument such as the BPI or the GCPS could be appropriate, since they include a variety of characteristics such as pain-related disability or duration. Applied to our included studies, in larger samples a measurement that takes less time could be preferred whereas in older populations, with potentially multimorbid participants, a more detailed instrument might be appropriate. This also means that future studies should be aware that instruments need to be fitted to the special needs of the concerning population. An instrument measuring pain in older depressed patients might have to be different to one used in their middle-aged counterpart. Therefore, guidelines should clearly recommend such uniform and standardized instruments to assess pain in depressed patients and vice versa with respect to the patients’ needs.

### Strengths and limitations

This is the first scoping review that examines co-occurring depression and pain in the general population. The review’s main strengths are the comprehensive search strategy, with no restrictions regarding the language and publication period of articles, and that studies from over 23 countries were included. There are, however, some important limitations to consider. First, since some articles did not report their primary objective in the title/abstract, we might have missed studies providing this information in the full text. To minimize this risk, we read 405 full texts and further performed a forward (cited) and backward (citation) analysis, which provided another 4,000 records that were screened and lead to the inclusion of 10 additional articles. However, due to limited resources we only performed one iteration. Second, the assessments for depression and pain of the included studies were very heterogenous regarding the general number of different measurements, the number of items applied or the format of the question-based measures. This limits the comparability of the outcomes presented for co-occurring depression and pain. However, since we focused to map the assessments used and did not further specify the desired results this was necessary to identify as many relevant studies as possible. During the analysis of the measurements, we were dependent on the information as reported by the included studies. Since the descriptions differed and we aimed to summarize the measurements in the most concise format, this could lead to incomplete or incorrect definitions. To gain a broader scope, we included studies with samples from the general population with extensive in- and exclusion criteria, accepting the resulting heterogeneity between the studies. We did not perform a quality assessment of the included studies. However, this is in line with the guidelines for conducting scoping reviews [18] and is based on the general aim for the scoping review to consider all available evidence.

## Conclusion

This scoping review provides a comprehensive overview on the assessment of co-occurring depression and pain in the general population and included 60 studies, of which only about one third initially focused on both conditions. We found a high comorbidity for both conditions (50.2% for pain by depression; 46.5% for depression by pain), with prevalences largely varying between studies. This also reflects the methodological heterogeneity with a wide range of instruments and question-based measures used for the assessment of depression and particularly pain. Depression was mostly assessed with standardized instruments, whereas pain was assessed with question-based measures often failing to address the multidimensionality of pain. This methodological heterogeneity highlights the need for a standardized initial assessment in patients presenting with either depression or pain also considering the multidimensionality of pain for a better informed treatment. This should also be considered in future studies and guideline recommendations especially in the general population.

## Supplementary Information

Below is the link to the electronic supplementary material.

Supplementary Material 1

Supplementary Material 2

## Data Availability

All data supporting the findings of this study are available within the paper and its Supplementary Information. For example, assessments of depression for each individual study are provided in Supplementary Table S3.
