# Supplementary material for: Assessment of depression and pain in the general population: a scoping review
Source: BMC Psychiatry. 2026 Aug 11;26:623. doi: 10.1186/s12888-026-08510-2 (PMC13471267; doi:10.1186/s12888-026-08510-2)
Supplement: Supplementary file 2 [file 12888_2026_8510_MOESM2_ESM.docx]

**Supplemental Material**

| Table s1. Search strategy………………………………………………………………………………………………………………………………………. | 2 |
| --- | --- |
| Table s2. Excluded records after full-text screening with reasons…………………………………………………………………………. | 3 |
| Table s3. Assessments of depression and pain for each individual study…………………………..………………………………….. | 24 |
| Table s4. Assessments of pain for each individual study………………………………………………………………………………………… | 29 |
| Table s5. Measurements for depression and pain in studies with the primary objective to assess both conditions.. | 36 |
| Table s6. Results for pain by depression or depression by pain as frequencies and/or effect estimates …………………………………………………………………………………….……………………………………………………………………………………… | 37 |
| Table s7. Operationalization of pain characteristics presence, duration/frequency and intensity………………………………………………………………………………………………………………………………………………………………………. | 48 |
| References (supplemental material)……………………………………………………………………………………………………………………. | 49 |

Table s1. Search strategy

| **Sources for search** | **Search terms** |
| --- | --- |
| ***Database (platform)*** | |
| MEDLINE  (PubMed)^a^ | ("depression"[mh] OR "depressive disorder"[mh] OR "dysthymic disorder"[mh] OR "depressive disorder, major"[mh] OR "depression"[tiab] OR "depressive"[tiab] OR "depressed"[tiab] OR "dysthymi*"[tiab] OR "mood disorder"[tiab:~2] OR "mood disorders"[tiab:~2] OR "psychological factors"[tiab:~2]) AND ("Pain"[mh] OR "Acute Pain"[mh] OR "Chronic Pain"[mh] OR "Pain"[tiab]) AND ("Epidemiology"[mh] OR "Risk Factors"[mh] OR "Comorbidity"[mh] OR "associat*"[tiab] OR "comorbid*"[tiab] OR "co morbid*"[tiab] OR "co-occur*"[tiab] OR "concurren*"[tiab] OR "risk factor*"[tiab] OR "predictor"[tiab] OR "predictors"[tiab] OR "epidemiolog*"[tiab]) AND ("Population"[mh] OR "Health Surveys"[mh] OR "general population"[tiab:~2] OR "health survey"[tiab:~2] OR "nationally survey"[tiab:~2] OR "nationally sample"[tiab:~2] OR "population based study"[tiab:~2] OR "national sample*"[tiab] OR "community sample*"[tiab] OR "representative sample*"[tiab] OR "population based sample*"[tiab] OR "population sample*"[tiab]) |
| Emabse  (Elsevier)^a^ | ('depression'/exp OR 'major depression'/de OR 'dysthymia'/de OR 'depression':ti,ab OR 'depressive':ti,ab OR 'depressed':ti,ab OR 'dysthymi*':ti,ab OR (mood NEXT/3 disorder*) OR (psychological NEXT/3 factors)) AND ('pain'/exp OR 'chronic pain'/de OR 'pain':ti,ab) AND ('epidemiology'/de OR 'risk factor'/exp OR 'comorbidity'/exp OR 'associat*':ti,ab OR 'comorbid*':ti,ab OR 'co morbid*':ti,ab OR 'co-occur*':ti,ab OR 'concurren*':ti,ab OR 'risk factor*':ti,ab OR 'predictor*':ti,ab OR 'epidemiolog*':ti,ab) AND ('population'/exp OR 'health survey'/exp OR (general NEXT/3 population) OR (health NEXT/3 survey) OR (nationally NEXT/3 survey) OR (nationally NEXT/3 sample) OR ('population based' NEXT/3 study) OR 'national sample*':ti,ab OR 'community sample*':ti,ab OR 'representative sample*':ti,ab OR 'population based sample*':ti,ab OR 'population sample*':ti,ab) |
| PsycINFO  (EBSCOhost)^a^ | (DE "Affective Disorders" OR DE "Major Depression" OR DE "Dysthymic Disorder" OR TI ("depression" OR "depressive" OR "depressed" OR "dysthymi*") OR AB ("depression" OR "depressive" OR "depressed" OR "dysthymi*") OR AB (mood W2 disorder* OR psychological W2 factors)) AND (DE "Pain" OR DE "Acute Pain" OR DE "Chronic Pain" OR TI "Pain" OR AB "Pain") AND (DE "Epidemiology" OR DE "Risk Factors" OR DE "Comorbidity" OR TI ("associat*" OR "comorbid*" OR "co morbid*" OR "co-occur*" OR "concurren*" OR "risk factor*" OR "predictor*" OR "epidemiolog*") OR AB ("associat*" OR "comorbid*" OR "co morbid*" OR "co-occur*" OR "concurren*" OR "risk factor*" OR "predictor*" OR "epidemiolog*")) AND (DE "Population" OR DE "Health Status" OR TI (general W2 population OR health W2 survey OR nationally W2 survey OR nationally W2 sample OR "population-based" W2 study) OR AB (general W2 population OR health W2 survey OR nationally W2 survey OR nationally W2 sample OR "population-based" W2 study) OR TI ("national sample*" OR "community sample*" OR "representative sample*" OR "population-based sample*" OR "population sample*") OR AB ("national sample*" OR "community sample*" OR "representative sample*" OR "population-based sample*" OR "population sample*")) |
| Notes:  Search strategies used in other systematic reviews for depression (Davison et al.(1)), pain (Beecham et al.(2)), comorbidity (Ipser et al.(3); Majewski et al.(4)), and general population (Xiong et al.(5)) were adapted. | |

Table s2. Excluded records after full-text screening with reasons

| **Record** | **Reason for exclusion** |
| --- | --- |
| ***Database search*** | |
| Abdin E, Chong SA, Vaingankar JA, et al. Impact of mental disorders and chronic physical conditions on quality-adjusted life years in Singapore. *Sci Rep*. 2020-2-142020;10(1):2695. doi:10.1038/s41598-020-59604-0 | 7 |
| Aggarwal VR, McBeth J, Zakrzewska JM, Lunt M, Macfarlane GJ. The epidemiology of chronic syndromes that are frequently unexplained: do they have common associated factors? *Int J Epidemiol*. 2006-4 2006;35(2):468-76. doi:10.1093/ije/dyi265 | 5 |
| Ahern MM, Hendryx M. Community participation and the emergence of late-life depressive symptoms: Differences between women and men. *Journal of Women's Health*. 2008 2008;17(9):1463-1470. doi:10.1089/jwh.2007.0752 | 2 |
| Ai Z, Tang C, Peng P, Wen X, Tang S. Prevalence and influencing factors of chronic pain in middle-aged and older adults in China: results of a nationally representative survey. *Front Public Health*. 2023 2023;11:1110216. doi:10.3389/fpubh.2023.1110216 | 4 |
| Al-Hamzawi AO, Rosellini AJ, Lindberg M, Petukhova M, Kessler RC, Bruffaerts R. The role of common mental and physical disorders in days out of role in the Iraqi general population: Results from the WHO World Mental Health Surveys. *Journal of Psychiatric Research*. 2014 2014;53(1):23-29. doi:10.1016/j.jpsychires.2014.02.006 | 7 |
| Al-Windi A. Depression in general practice. *Nord J Psychiatry*. 2005 2005;59(4):272-7. doi:10.1080/08039480500227733 | 4 |
| Alfonso H, Beer C, Yeap BB, Hankey GJ, Flicker L, Almeida OP. Perception of worsening health predicts mortality in older men: The Health in Men Study (HIMS). *Archives of Gerontology and Geriatrics*. 2012 2012;55(2):363-368. doi:10.1016/j.archger.2012.04.005 | 7 |
| Alonso J, Petukhova M, Vilagut G, et al. Days out of role due to common physical and mental conditions: Results from the WHO World Mental Health surveys. *Molecular Psychiatry*. 2011 2011;16(12):1234-1246. doi:10.1038/mp.2010.101 | 7 |
| Anand N, Brian M, Onojighofia T, Akindele B, Hubbard J, Schwarz D. Factors that might affect pain perception: A study on the role of age, gender and depression on pain perception. *PM and R*. 2015 2015;7(9):S90-S91. doi:10.1007/978-3-030-87266-3_39 | 1 |
| Anandakumar D, Ratnatunga SS, Dayabandara M, Hanwella R, de Silva VA. Depressive disorder in patients attending the outpatient department of a tertiary care hospital in Colombo. *Ceylon Med J*. 2016-9 2016;61(3):118-122. doi:10.4038/cmj.v61i3.8347 | 4 |
| Andersson HI. The epidemiology of chronic pain in a Swedish rural area. *Quality of Life Research*. 1994 1994;3:S19-S26. doi:10.1007/BF00433371 | 4 |
| Andersson HI, Ejlertsson G, Leden I, Scherstén B. Impact of chronic pain on health care seeking, self care, and medication. Results from a population-based Swedish study. *J Epidemiol Community Health*. 1999-8 1999;53(8):503-9. doi:10.1136/jech.53.8.503 | 4 |
| Andorsen OF, Ahmed LA, Emaus N, Klouman E. A prospective cohort study on risk factors of musculoskeletal complaints (pain and/or stiffness) in a general population. The Tromsø study. *PLoS One*. 2017 2017;12(7):e0181417. doi:10.1371/journal.pone.0181417 | 5 |
| Andreasson A, Tognetti A, Jones M, Lekander M, Lasselin J. Assessing sickness behavior in the French: Validation of the French translation of the sickness questionnaire (SicknessQ) in a non-clinical French population. *Brain Behav Immun Health*. 2023-12 2023;34:100708. doi:10.1016/j.bbih.2023.100708 | 4 |
| Angst F, Benz T, Lehmann S, et al. Extended overview of the longitudinal pain-depression association: A comparison of six cohorts treated for specific chronic pain conditions. *J Affect Disord*. 2020-8-1 2020;273:508-516. doi:10.1016/j.jad.2020.05.044 | 1 |
| Ansari S, Anand A, Hossain B. Multimorbidity and depression among older adults in India: Mediating role of functional and behavioural health. *PLoS One*. 2022 2022;17(6):e0269646. doi:10.1371/journal.pone.0269646 | 4 |
| Antonopoulou MD, Alegakis AK, Hadjipavlou AG, Lionis CD. Studying the association between musculoskeletal disorders, quality of life and mental health. A primary care pilot study in rural Crete, Greece. *BMC Musculoskelet Disord*. 2009-11-20 2009;10:143. doi:10.1186/1471-2474-10-143 | 6 |
| Aparecida Damasceno D, Ferreira Aleixo G, Barbosa Luciano J, Nogueira CM, Pinto JM. Factors Related to Recurrent Falls Among Older Adults Attending Primary Health Care: A Biopsychosocial Perspective. *Exp Aging Res*. 2024-5 2024;50(3):348-359. doi:10.1080/0361073x.2023.2195293 | 7 |
| Arnow BA, Blasey CM, Constantino MJ, et al. Catastrophizing, depression and pain-related disability. *Gen Hosp Psychiatry*. 2011-3 2011;33(2):150-6. doi:10.1016/j.genhosppsych.2010.12.008 | 6 |
| Asbury M, Helstrom A, Benson A, et al. Experience of pain in the elderly in relation to mood, anxiety and physical quality of life in a pace/ bhl program. *American Journal* *of Geriatric Psychiatry*. 2013 2013;21(3):S109-S110. doi:10.1016/j.jagp.2012.12.146 | 1 |
| Ashworth M, Durbaba S, Whitney D, Crompton J, Wright M, Dodhia H. Journey to multimorbidity: longitudinal analysis exploring cardiovascular risk factors and sociodemographic determinants in an urban setting. *BMJ Open*. 2019-12-23 2019;9(12):e031649. doi:10.1136/bmjopen-2019-031649 | 7 |
| Askari MS, Andrade LH, Filho AC, et al. Dual burden of chronic physical diseases and anxiety/mood disorders among São Paulo megacity mental health survey sample, Brazil. *Journal of Affective Disorders*. 2017 2017;220:1-7. doi:10.1016/j.jad.2017.05.027 | 6 |
| Atherton K, Power C. Health inequalities with the National Statistics-Socioeconomic classification: disease risk factors and health in the 1958 British birth cohort. *Eur J Public Health*. 2007-10 2007;17(5):486-91. doi:10.1093/eurpub/ckl269 | 7 |
| Attal N, Lanteri-Minet M, Laurent B, Fermanian J, Bouhassira D. The specific disease burden of neuropathic pain: Results of a French nationwide survey. *Pain*. 2011 2011;152(12):2836-2843. doi:10.1016/j.pain.2011.09.014 | 7 |
| Azevedo LF, Costa-Pereira A, Mendonça L, Dias CC, Castro-Lopes JM. Epidemiology of chronic pain: a population-based nationwide study on its prevalence, characteristics and associated disability in Portugal. *J Pain*. 2012-8 2012;13(8):773-83. doi:10.1016/j.jpain.2012.05.012 | 5 |
| Azevedo LF, Costa-Pereira A, Mendonça L, Dias CC, Castro-Lopes JM. Chronic pain and health services utilization: is there overuse of diagnostic tests and inequalities in nonpharmacologic treatment methods utilization? *Med Care*. 2013-10 2013;51(10):859-69. doi:10.1097/MLR.0b013e3182a53e4e | 7 |
| Backe IF, Patil GG, Nes RB, Clench-Aas J. The relationship between physical functional limitations, and psychological distress: Considering a possible mediating role of pain, social support and sense of mastery. *SSM Popul Health*. 2018-4 2018;4:153-163. doi:10.1016/j.ssmph.2017.12.005 | 7 |
| Bailly F, Petit A, Kab S, Foltz V, Badard M, Fautrel B. DEMOGRAPHICAL, SOCIOPROFESSIONAL FACTORS AND HABITS ASSOCIATED WITH CHRONIC WIDESPREAD PAIN IN A LARGE GENERAL POPULATION COHORT. *Annals of the Rheumatic Diseases*. 2023 2023;82:1914-1915. doi:10.1136/annrheumdis-2023-eular.1875 | 1 |
| Baker TA, Buchanan NT, Corson N. Factors influencing chronic pain intensity in older black women: examining depression, locus of control, and physical health. *J Womens Health (Larchmt)*. 2008-6 2008;17(5):869-78. doi:10.1089/jwh.2007.0452 | 2 |
| Baker TA, Whitfield KE. Physical functioning in older blacks: an exploratory study identifying psychosocial and clinical predictors. *J Natl Med Assoc*. 2006-7 2006;98(7):1114-20. | 2 |
| Baladón L, Rubio-Valera M, Serrano-Blanco A, Palao DJ, Fernández A. Gender differences in the impact of mental disorders and chronic physical conditions on health related quality of life among non-demented primary care elderly patients. *Quality of Life Research*. 2016 2016;25(6):1461-1474. doi:10.1007/s11136-015-1182-5 | 6 |
| Barcellos S, Jacobson M, Stone AA. Varied and unexpected changes in the well-being of seniors in the United States amid the COVID-19 pandemic. *PLoS One*. 2021 2021;16(6):e0252962. doi:10.1371/journal.pone.0252962 | 7 |
| Barkow K, Heun R, Ustün TB, Maier W. Identification of items which predict later development of depression in primary health care. *Eur Arch Psychiatry Clin Neurosci*. 2001 2001;251:Ii21-6. doi:10.1007/bf03035122 | 5 |
| Barry DT, Glenn CP, Hoff RA, Potenza MN. Group differences in pain interference, psychiatric disorders, and general medical conditions among Hispanics and whites in the U.S. general population. *Psychiatry Res*. 2017-12 2017;258:337-343. doi:10.1016/j.psychres.2017.08.049 | 5 |
| Barry DT, Pilver C, Potenza MN, Desai RA. Prevalence and psychiatric correlates of pain interference among men and women in the general population. *Journal of Psychiatric Research*. 2012 2012;46(1):118-127. doi:10.1016/j.jpsychires.2011.09.004 | 5 |
| Barry DT, Pilver CE, Hoff RA, Potenza MN. Pain interference and incident mood, anxiety, and substance-use disorders: findings from a representative sample of men and women in the general population. *J Psychiatr Res*. 2013-11 2013;47(11):1658-64. doi:10.1016/j.jpsychires.2013.08.004 | 5 |
| Baune BT, Caniato RN, Garcia-Alcaraz MA, Berger K. Combined effects of major depression, pain and somatic disorders on general functioning in the general adult population. *Pain*. 2008-8-31 2008;138(2):310-317. doi:10.1016/j.pain.2008.01.002 | 5 |
| Bazargan M, Yazdanshenas H, Gordon D, Orum G. Pain in Community-Dwelling Elderly African Americans. *J Aging Health*. 2016-4 2016;28(3):403-25. doi:10.1177/0898264315592600 | 2 |
| Beesdo K, Jacobi F, Hoyer J, Low NC, Höfler M, Wittchen HU. Pain associated with specific anxiety and depressive disorders in a nationally representative population sample. *Soc Psychiatry Psychiatr Epidemiol*. 2010-1 2010;45(1):89-104. doi:10.1007/s00127-009-0045-1 | 5 |
| Benjet C, Casanova L, Borges G, Medina-Mora ME. [The impact of common mental and physical health conditions on days out of role: costs for the individual and society]. *Salud Publica Mex*. 2013-5 2013;55(3):248-56. | 7 |
| Bergh I, Steen G, Waern M, et al. Pain and its relation to cognitive function and depressive symptoms: a Swedish population study of 70-year-old men and women. *J Pain Symptom Manage*. 2003-10 2003;26(4):903-12. doi:10.1016/s0885-3924(03)00329-4 | 7 |
| Beutel ME, Wiltink J, Ghaemi Kerahrodi J, et al. Somatic symptom load in men and women from middle to high age in the Gutenberg Health Study - association with psychosocial and somatic factors. *Sci Rep*. 2019-3-14 2019;9(1):4610. doi:10.1038/s41598-019-40709-0 | 7 |
| Bierman A, Lee Y. Chronic Pain and Psychological Distress Among Older Adults: A National Longitudinal Study. *Res Aging*. 2018-6 2018;40(5):432-455. doi:10.1177/0164027517704970 | 7 |
| Blay SL, Andreoli SB, Dewey ME, Gastal FL. Co-occurrence of chronic physical pain and psychiatric morbidity in a community sample of older people. *International Journal of Geriatric Psychiatry*. 2007 2007;22(9):902-908. doi:10.1002/gps.1761 | 6 |
| Boerma T, Hosseinpoor AR, Verdes E, Chatterji S. A global assessment of the gender gap in self-reported health with survey data from 59 countries. *BMC Public Health*. 2016-7-30 2016;16:675. doi:10.1186/s12889-016-3352-y | 7 |
| Bonnewyn A, Katona C, Bruffaerts R, et al. Pain and depression in older people: comorbidity and patterns of help seeking. *J Affect Disord*. 2009-10 2009;117(3):193-6. doi:10.1016/j.jad.2009.01.012 | 7 |
| Borges LJ, Benedetti TR, Xavier AJ, d'Orsi E. Associated factors of depressive symptoms in the elderly: EpiFloripa study. *Rev Saude Publica*. 2013-8 2013;47(4):701-10. doi:10.1590/s0034-8910.2013047003844 | 4 |
| Boyd E, Quiton R, Leibel D, et al. Pain and poverty: A study of the intersectionality of demographic and socioeconomic factors on pain interference. *Journal of Pain*. 2016 2016;17(4):S35-S36. doi: | 1 |
| Buist-Bouwman MA, de Graaf R, Vollebergh WA, Ormel J. Comorbidity of physical and mental disorders and the effect on work-loss days. *Acta Psychiatr Scand*. 2005-6 2005;111(6):436-43. doi:10.1111/j.1600-0447.2005.00513.x | 5 |
| Buys L, Roberto KA, Miller E, Blieszner R. Prevalence and predictors of depressive symptoms among rural older Australians and Americans. *Aust J Rural Health*. 2008-2 2008;16(1):33-9. doi:10.1111/j.1440-1584.2007.00948.x | 7 |
| Byers AL, Bruce ML. The epidemiology of major depressive disorder and comorbidity in older adults. *American Journal of Geriatric Psychiatry*. 2009 2009;17:A87. doi:10.1097/01.JGP.0000346964.46544.ec | 1 |
| Cai B, Oderda GM. The association between pain and depression and some determinants of depression for the general population of the United States. *J Pain Palliat Care Pharmacother*. 2012-9 2012;26(3):257-65. doi:10.3109/15360288.2012.703292 | 5 |
| Calabrese SK, Lyness JM, Sörensen S, Duberstein PR. Personality and the association of pain and depression. *Am J Geriatr Psychiatry*. 2006-6 2006;14(6):546-9. doi:10.1097/01.Jgp.0000218323.20981.10 | 7 |
| Calvó-Perxas L, Vilalta-Franch J, Turró-Garriga O, López-Pousa S, Garre-Olmo J. Gender differences in depression and pain: A two year follow-up study of the Survey of Health, Ageing and Retirement in Europe. *J Affect Disord*. 2016-3-15 2016;193:157-64. doi:10.1016/j.jad.2015.12.034 | 5 |
| Caramenti M, Castiglioni I. Determinants of Self-Perceived Health: The Importance of Physical Well-Being but Also of Mental Health and Cognitive Functioning. *Behav Sci (Basel)*. 2022-12-6 2022;12(12)doi:10.3390/bs12120498 | 7 |
| Castelli MS. The relationship of pain, sleep, mood and function in a community sample. *Sleep*. 2015 2015;38:A300. | 1 |
| Caudill-Slosberg M, Golden D, Batchvarov V, et al. Musculoskeletal/chronic pain assessment during nurse based screening clinics for non-communicable diseases: Kenya. *Journal of Pain*. 2013 2013;14(4):S13. doi:10.1016/j.jpain.2013.01.062 | 1 |
| Chang SM, Kim JH, Won SH. Mental-physical comorbidity in Korean adults: Results from a nationwide general population survey in Korea. *European Neuropsychopharmacology*. 2016 2016;26:S351-S352. doi: 10.4306/pi.2016.13.5.496 | 6 |
| Chin WY, Choi EPH, Wan EYF, Lam CLK. Health-related quality of life mediates associations between multi-morbidity and depressive symptoms in Chinese primary care patients. *Family Practice*. 2016 2016;33(1):61-68. doi:10.1093/fampra/cmv090 | 7 |
| Chiu YH, Silman AJ, Macfarlane GJ, et al. Poor sleep and depression are independently associated with a reduced pain threshold. Results of a population based study. *Pain*. 2005-6 2005;115(3):316-321. doi:10.1016/j.pain.2005.03.009 | 5 |
| Chodosh J, Ferrell BA, Shekelle PG, Wenger NS. Quality indicators for pain management in vulnerable elders. *Annals of Internal Medicine*. 2001 2001;135(8):731-735. doi:10.7326/0003-4819-135-8_part_2-200110161-00012 | 1 |
| Chong SA, Abdin E, Nan L, Vaingankar JA, Subramaniam M. Prevalence and impact of mental and physical comorbidity in the adult Singapore population. *Annals of the Academy of Medicine Singapore*. 2012 2012;41(3):105-114. | 5 |
| Chowdhury PP, Balluz L, Strine TW. Health-related quality of life among minority populations in the United States, BRFSS 2001-2002. *Ethn Dis*. 2008 2008;18(4):483-7. | 7 |
| Christensen KS, Sokolowski I, Olesen F. Case-finding and risk-group screening for depression in primary care. *Scand J Prim Health Care*. 2011-6 2011;29(2):80-4. doi:10.3109/02813432.2011.554009 | 4 |
| Clausen T, Romøren TI, Ferreira M, Kristensen P, Ingstad B, Holmboe-Ottesen G. Chronic diseases and health inequalities in older persons in Botswana (southern Africa): a national survey. *J Nutr Health Aging*. 2005-11 2005;9(6):455-61. | 5 |
| Cordingley L, Nicholl B, McBeth J. Personal social support networks are associated with the onset of chronic widespread pain. *Rheumatology*. 2009 2009;48:i95. doi:10.1093/rheumatology/kep728 | 1 |
| Costantino H, Ken-Opurum J, Jung S, Kim B, Grillo V, Stankus A. PMH10 Depression Severity Is Associated with Physical PAIN Severity across Countries. *Value in Health Regional Issues*. 2020 2020;22:S63. doi:10.1016/j.vhri.2020.07.332 | 1 |
| Coste J, Valderas JM, Carcaillon-Bentata L. Estimating and characterizing the burden of multimorbidity in the community: A comprehensive multistep analysis of two large nationwide representative surveys in France. *PLoS Med*. 2021-4 2021;18(4):e1003584. doi:10.1371/journal.pmed.1003584 | 5 |
| Cöster L, Kendall S, Gerdle B, Henriksson C, Henriksson KG, Bengtsson A. Chronic widespread musculoskeletal pain--A comparison of those who meet criteria for fibromyalgia and those who do not. *European Journal of Pain*. 2008 2008;12(5):600-610. doi:10.1016/j.ejpain.2007.10.001 | 2 |
| Creed FH, Tomenson B, Chew-Graham C, et al. Multiple somatic symptoms predict impaired health status in functional somatic syndromes. *Int J Behav Med*. 2013-6 2013;20(2):194-205. doi:10.1007/s12529-012-9257-y | 5 |
| Croft P, Rigby AS, Boswell R, Schollum J, Silman A. The prevalence of chronic widespread pain in the general population. *J Rheumatol*. 1993-4 1993;20(4):710-3. | 5 |
| Czaderny K. Risk factors for depression. New evidence on selenium deficiency and depressive disorders. *Psychiatr Pol*. 2020-12-31 2020;54(6):1109-1121. doi:10.12740/pp/112967 | 7 |
| de Heer EW, Gerrits MM, Beekman AT, et al. The association of depression and anxiety with pain: a study from NESDA. *PLoS One*. 2014 2014;9(10):e106907. doi:10.1371/journal.pone.0106907 | 2 |
| de Heer EW, Ten Have M, van Marwijk HWJ, et al. Pain as a risk factor for common mental disorders. Results from the Netherlands Mental Health Survey and Incidence Study-2: a longitudinal, population-based study. *Pain*. 2018-4 2018;159(4):712-718. doi:10.1097/j.pain.0000000000001133 | 6 |
| De La Rosa JS, Brady BR, Herder KE, et al. The unmet mental health needs of U.S. adults living with chronic pain. *Pain*. 2024-7-30 2024;165(12):2877-87. doi:10.1097/j.pain.0000000000003340 | 2 |
| De La Rosa JS, Brady BR, Ibrahim MM, et al. Co-occurrence of chronic pain and anxiety/depression symptoms in U.S. adults: prevalence, functional impacts, and opportunities. *Pain*. 2024-3-1 2024;165(3):666-673. doi:10.1097/j.pain.0000000000003056 | 6 |
| de la Vega R, Groenewald C, Bromberg MH, Beals-Erickson SE, Palermo TM. Chronic pain prevalence and associated factors in adolescents with and without physical disabilities. *Dev Med Child Neurol*. 2018-6 2018;60(6):596-601. doi:10.1111/dmcn.13705 | 2 |
| de Vitta A, Machado Maciel N, Bento TPF, Genebra C, Simeão S. Multisite musculoskeletal pain in the general population: a cross-sectional survey. *Sao Paulo Med J*. 2022-1 2022;140(1):24-32. doi:10.1590/1516-3180.2021.0134.R1.05052021 | 5 |
| Demyttenaere K, Bonnewyn A, Bruffaerts R, Brugha T, De Graaf R, Alonso J. Comorbid painful physical symptoms and depression: prevalence, work loss, and help seeking. *J Affect Disord*. 2006-6 2006;92(2):185-93. doi:10.1016/j.jad.2006.01.007 | 5 |
| Dhanju S, Kennedy SH, Abbey S, et al. The impact of comorbid pain and depression in the United States: results from a nationally representative survey. *Scand J Pain*. 2019-4-24 2019;19(2):319-325. doi:10.1515/sjpain-2018-0323 | 5 |
| Didoné LS, Jesus ITM, Santos-Orlandi AA, et al. Factors associated with depressive symptoms in older adults in context of social vulnerability. *Revista brasileira de enfermagem*. 2020 2020;73:e20190107. doi:10.1590/0034-7167-2019-0107 | 2 |
| Ding ZJ, Wang GP, Zhang YL, et al. [Epidemiological survey of mental disorders in the rural left behind elderly aged 60 years and older in Gansu]. *Zhonghua Yi Xue Za Zhi*. 2019-8-20 2019;99(31):2429-2434. doi:10.3760/cma.j.issn.0376-2491.2019.31.005 | 7 |
| Dominick CH, Blyth FM, Nicholas MK. Unpacking the burden: understanding the relationships between chronic pain and comorbidity in the general population. *Pain*. 2012-2 2012;153(2):293-304. doi:10.1016/j.pain.2011.09.018 | 5 |
| Dorner TE, Stein KV, Hahne J, Wepner F, Friedrich M, Mittendorfer-Rutz E. How are socio-demographic and psycho-social factors associated with the prevalence and chronicity of severe pain in 14 different body sites? A cross-sectional population-based survey. *Wien Klin Wochenschr*. 2018-1 2018;130(1):14-22. doi:10.1007/s00508-017-1223-x | 6 |
| Dragioti E, Tsamakis K, Larsson B, Gerdle B. Predictive association between immigration status and chronic pain in the general population: results from the SwePain cohort. *BMC Public Health*. 2020-9-29 2020;20(1):1462. doi:10.1186/s12889-020-09546-z | 7 |
| Duran J, Zitko P, Margozzini P. Chronic musculoskeletal pain and chronic widespread pain in Chile: Prevalence and associated factors. *Annals of the Rheumatic Diseases*. 2020 2020;79:466. doi:10.1136/annrheumdis-2020-eular.6692 | 1 |
| Eytan A, Alberque C. Depression and somatic comorbidity. *Aust N Z J Psychiatry*. 2001-10 2001;35(5):692-3. doi:10.1080/0004867010060522 | 1 |
| Fekadu A, O'Donovan MC, Alem A, et al. Validity of the concept of minor depression in a developing country setting. *J Nerv Ment Dis*. 2008-1 2008;196(1):22-8. doi:10.1097/NMD.0b013e31815fa4d4 | 2 |
| Felez-Nobrega M, Haro JM, Koyanagi A. Multimorbidity, depression with anxiety symptoms, and decrements in health in 47 low- and middle-income countries. *J Affect Disord*. 2022-11-15 2022;317:176-184. doi:10.1016/j.jad.2022.08.110 | 5 |
| Fernandes GS, Parekh SM, Moses J, et al. Depressive symptoms and the general health of retired professional footballers compared with the general population in the UK: a case-control study. *BMJ Open*. 2019-9-8 2019;9(9):e030056. doi:10.1136/bmjopen-2019-030056 | 7 |
| Fernández A, Saameño JA, Pinto-Meza A, et al. Burden of chronic physical conditions and mental disorders in primary care. *Br J Psychiatry*. 2010-4 2010;196(4):302-9. doi:10.1192/bjp.bp.109.074211 | 5 |
| Fernández M, Lera L, Albala C. [Association between pain and depression in chilean older adults. Reality that requires an immediate approach]. *Rev Med Chil*. 2024-1 2024;152(1):69-79. doi:10.4067/s0034-98872024000100069 |  |
| Ferreira KASL, Bastos TRPD, de Andrade DC, et al. Prevalence of chronic pain in a metropolitan area of a developing country: A population-based study. *Arquivos de Neuro-Psiquiatria*. 2016 2016;74(12):990-998. doi:10.1590/0004-282x20160156 | 4 |
| Ferreira KDS, Schwalbach MT, Schwalbach J, Speciali JG. A pain unit in a developing country: Epidemiology of chronic pain in maputo, Mozambique. *Pain Medicine (United States)*. 2014 2014;15(11):1986-1988. doi:10.1111/pme.12544 | 1 |
| Fielding R, Wong WS. Prevalence of chronic pain, insomnia, and fatigue in Hong Kong. *Hong Kong Med J*. 2012-8 2012;18:9-12. | 6 |
| Forlani C, Atti AR, Ferrari B, Dalmonte E, De Ronchi D. Depression, cognitive impairment, somatic complaints and disability. data from the “Faenza community aging study”. *European Psychiatry*. 2009 2009;24:S780. doi:10.1016/S0924-9338(09)71013-2 | 1 |
| Frank P, Batty GD, Pentti J, et al. Association Between Depression and Physical Conditions Requiring Hospitalization. *JAMA Psychiatry*. 2023-7-1 2023;80(7):690-699. doi:10.1001/jamapsychiatry.2023.0777 | 5 |
| Fröhlich C, Jacobi F, Wittchen H-U. DSM-IV pain disorder in the general population: An exploration of the structure and threshold of medically unexplained pain symptoms. *European Archives of Psychiatry and Clinical Neuroscience*. 2006 2006;256(3):187-196. doi:10.1007/s00406-005-0625-3 | 5 |
| Gadermann AM, Alonso J, Vilagut G, Zaslavsky AM, Kessler RC. Comorbidity and disease burden in the National Comorbidity Survey Replication (NCS-R). *Depress Anxiety*. 2012-9 2012;29(9):797-806. doi:10.1002/da.21924 | 7 |
| Gajović G, Kocić S, Radovanović S, Vukomanović IS, Janićijević K, Radević S. Prevalence of depression in elderly and relations to chronic diseases. *Vojnosanitetski Pregled*. 2021 2021;78(5):504-510. doi:10.2298/VSP190719098G | 4 |
| Gameroff MJ, Olfson M. Major depressive disorder, somatic pain, and health care costs in an urban primary care practice. *J Clin Psychiatry*. 2006-8 2006;67(8):1232-9. doi:10.4088/jcp.v67n0809 | 2 |
| García-Esquinas E, Rodríguez-Sánchez I, Ortolá R, et al. Gender Differences in Pain Risk in Old Age: Magnitude and Contributors. *Mayo Clin Proc*. 2019-9 2019;94(9):1707-1717. doi:10.1016/j.mayocp.2019.03.034 | 7 |
| Gaynes BN, Burns BJ, Tweed DL, Erickson P. Depression and health-related quality of life. *J Nerv Ment Dis*. 2002-12 2002;190(12):799-806. doi:10.1097/00005053-200212000-00001 | 4 |
| Gellert P, Ernsting C, Salm F, Oedekoven M, Kanzler M, Kuhlmey A. Disease-specific knowledge in individuals with and without chronic conditions. *Journal of Public Health*. 2018 2018;26(2):145-150. doi:10.1007/s10389-017-0855-7 | 7 |
| Glette M, Stiles TC, Jensen MP, Nilsen TIL, Borchgrevink PC, Landmark T. Impact of pain and catastrophizing on the long-term course of depression in the general population: the HUNT pain study. *Pain*. 2021-6-1 2021;162(6):1650-1658. doi:10.1097/j.pain.0000000000002168 | 6 |
| Gómez-Restrepo C, Bohórquez A, Pinto Masis D, Gil Laverde JFA, Sepúlveda MR, Díaz-Granados N. The prevalence of and factors associated with depression in Colombia. *Revista Panamericana de Salud Publica/Pan American Journal of Public Health*. 2004 2004;16(6):378-386. doi:10.1590/S1020-49892004001200003 | 6 |
| Gonçalves B, Fagulha T, Ferreira A, Reis N. Depressive symptoms and pain complaints as predictors of later development of depression in Portuguese middle-aged women. *Health Care Women Int*. 2014 2014;35(11):1228-44. doi:10.1080/07399332.2013.862795 | 5 |
| Grembowski D, Paschane D, Diehr P, Katon W, Martin D, Patrick DL. Managed care and patient ratings of the quality of specialty care among patients with pain or depressive symptoms. *BMC Health Serv Res*. 2007-2-16 2007;7:22. doi:10.1186/1472-6963-7-22 | 2 |
| Griffith LE, Gilsing A, Mangin D, et al. Multimorbidity Frameworks Impact Prevalence and Relationships with Patient-Important Outcomes. *Journal of the American Geriatrics Society*. 2019 2019;67(8):1632-1640. doi:10.1111/jgs.15921 | 5 |
| Gureje O, Ademola A, Olley BO. Depression and disability: comparisons with common physical conditions in the Ibadan study of aging. *J Am Geriatr Soc*. 2008-11 2008;56(11):2033-8. doi:10.1111/j.1532-5415.2008.01956.x | 5 |
| Gureje O, Kola L, Ademola A, Olley BO. Profile, comorbidity and impact of insomnia in the Ibadan study of ageing. *Int J Geriatr Psychiatry*. 2009-7 2009;24(7):686-93. doi:10.1002/gps.2180 | 7 |
| Gureje O, Von Korff M, Simon GE, Gater R. Persistent pain and well-being: a World Health Organization Study in Primary Care. *Jama*. 1998-7-8 1998;280(2):147-51. doi:10.1001/jama.280.2.147 | 5 |
| Hajek A, König HH. The moderating role of flexible goal adjustment in the link between pain and depressive symptoms: findings based on a nationally representative sample of older adults. *Psychogeriatrics*. 2020-9 2020;20(5):602-607. doi:10.1111/psyg.12537 | 7 |
| Hannerz H, Holtermann A, Madsen IEH. Musculoskeletal pain as a predictor for depression in the general working population of Denmark. *Scand J Public Health*. 2021-8 2021;49(6):589-597. doi:10.1177/1403494819875337 | 2 |
| Hao RQ, Wang XM, Du ML, et al. [Status and related factors of self-reported pain among working-age population in Inner Mongolia Autonomous Region]. *Zhonghua Liu Xing Bing Xue Za Zhi*. 2016-12-10 2016;37(12):1625-1629. doi:10.3760/cma.j.issn.0254-6450.2016.12.014 | 6 |
| Hauber D, Zank S. WWII trauma impacts physical and mental health in the oldest old: results from a German population-based study. *Aging Ment Health*. 2022-4 2022;26(4):834-842. doi:10.1080/13607863.2021.1876637 | 2 |
| Häuser W, Glaesmer H, Schmutzer G, Brähler E. Widespread pain in older Germans is associated with posttraumatic stress disorder and lifetime employment status--results of a cross-sectional survey with a representative population sample. *Pain*. 2012-12 2012;153(12):2466-2472. doi:10.1016/j.pain.2012.09.006 | 5 |
| Häuser W, Schmutzer G, Glaesmer H, Brähler E. [Prevalence and predictors of pain in several body regions. Results of a representative German population survey]. *Schmerz*. 2009-10 2009;23(5):461-70. doi:10.1007/s00482-009-0817-2 | 5 |
| Higgins C, Sharma S, Bimali I, et al. Cross-sectional study examining the epidemiology of chronic pain in Nepal. *Pain Rep*. 2023-3 2023;8(2):e1067. doi:10.1097/pr9.0000000000001067 | 7 |
| Hirsch JK, Sirois FM, Molnar D, Chang EC. Pain and Depressive Symptoms in Primary Care: Moderating Role of Positive and Negative Affect. *Clin J Pain*. 2016-7 2016;32(7):562-7. doi:10.1097/ajp.0000000000000292 | 7 |
| Hoebel J, Kuntz B, Müters S, Lampert T. [Subjective social status and health-related quality of life among adults in Germany. Results from the German General Social Survey (ALLBUS 2010)]. *Gesundheitswesen*. 2013-10 2013;75(10):643-51. doi:10.1055/s-0032-1333211 | 4 |
| Hooten MW, Shi Y, Gazelka HM, Warner DO. The effects of depression and smoking on pain severity and opioid use in patients with chronic pain. *Pain*. 2011-1 2011;152(1):223-229. doi:10.1016/j.pain.2010.10.045 | 2 |
| Hu H, Liu W, Zhang S, Pan J, Zheng X. Depression mediates the relationship between smoking and pain: Evidence from a nationally representative study in a low- and middle-income country. *Addict Behav*. 2021-8 2021;119:106937. doi:10.1016/j.addbeh.2021.106937 | 7 |
| Hu L, Liu Z-Z, Wang Z-Y, Jia C-X, Liu X. Associations between pain and depressive symptoms: A longitudinal study of Chinese adolescents. *Journal of Affective Disorders*. 2022 2022;299:675-681. doi:10.1016/j.jad.2021.12.095 | 2 |
| Huang DL, Bardhan I, Shin J, Karp JF, Park M. Chronic Pain and Mood Disorders in Asian Americans. *Asian Pac Isl Nurs J*. 2021 2021;5(4):217-226. doi:10.31372/20200504.1115 | 5 |
| Ishizaki T, Yoshida H, Suzuki T, Shibata H. The association between self-rated health status and increasing age among older Japanese living in a rural community over a 6-year period: a longitudinal data analysis. *Gerontology*. 2009 2009;55(3):344-52. doi:10.1159/000183749 | 7 |
| Jakobsson U. The epidemiology of chronic pain in a general population: results of a survey in southern Sweden. *Scand J Rheumatol*. 2010 2010;39(5):421-9. doi:10.3109/03009741003685616 | 4 |
| Jakobsson U, Hallberg IR, Westergren A. Overall and health related quality of life among the oldest old in pain. *Qual Life Res*. 2004-2 2004;13(1):125-36. doi:10.1023/b:Qure.0000015286.68287.66 | 2 |
| Jakobsson U, Hallberg IR, Westergren A. Exploring determinants for quality of life among older people in pain and in need of help for daily living. *J Clin Nurs*. 2007-3 2007;16(3):95-104. doi:10.1111/j.1365-2702.2006.01584.x | 2 |
| Jakobsson U, Klevsgård R, Westergren A, Hallberg IR. Old People in Pain: A Comparative Study. *Journal of Pain and Symptom Management*. 2003 2003;26(1):625-636. doi:10.1016/S0885-3924(03)00145-3 | 2 |
| James RJE, Ferguson E. Depression, Cognition, and Pain: Exploring Individual, Cultural and Country-Level Effects Across Europe. *J Pain*. 2023-6 2023;24(6):1104-1115. doi:10.1016/j.jpain.2023.03.006 | 7 |
| Janosky JE, South-Paul JE, Lin CJ. Pain and depression in a cohort of underserved, community-dwelling primary care patients. *Journal of the American Board of Family Medicine*. 2012 2012;25(3):300-307. doi:10.3122/jabfm.2012.03.080138 | 5 |
| Järemo P, Arman M, Gerdle B, Gottberg K. Facing Invalidation: A Further Challenge when Living with Chronic Widespread Pain. *J Rehabil Med*. 2022-4-22 2022;54:jrm00280. doi:10.2340/jrm.v54.67 | 2 |
| Järemo P, Arman M, Gerdle B, Larsson B, Gottberg K. Illness beliefs among patients with chronic widespread pain - associations with self-reported health status, anxiety and depressive symptoms and impact of pain. *BMC Psychol*. 2017-7-5 2017;5(1):24. doi:10.1186/s40359-017-0192-1 | 2 |
| Jensen B, Rogers HL, Perrin PB, et al. Influence of anxiety and depressive symptoms on bodily pain: A longitudinal comparison between colombian coronary heart disease patients and controls. *Psychosomatic Medicine*. 2013 2013;75(3):A100-A101. doi:10.1097/01.psy.0000429452.16135.1b | 1 |
| Jiménez-Trujillo I, López-de-Andrés A, Del Barrio JL, Hernández-Barrera V, Valero-de-Bernabé M, Jiménez-García R. Gender Differences in the Prevalence and Characteristics of Pain in Spain: Report from a Population-Based Study. *Pain Med*. 2019-12-1 2019;20(12):2349-2359. doi:10.1093/pm/pnz004 | 5 |
| Joffe H, Chang Y, Dhaliwal S, et al. Lifetime history of depression and anxiety disorders as a predictor of quality of life in midlife women in the absence of current illness episodes. *Arch Gen Psychiatry*. 2012-5 2012;69(5):484-92. doi:10.1001/archgenpsychiatry.2011.1578 | 2 |
| Jones EA, McBeth J, Nicholl B, et al. What characterizes persons who do not report musculoskeletal pain? Results from a 4-year Population-based longitudinal study (the Epifund study). *J Rheumatol*. 2009-5 2009;36(5):1071-7. doi:10.3899/jrheum.080541 | 5 |
| Jung IK, Kim SJ. Somatic symptoms and late life depression: Focus on the association of the number of somatic symptoms with severity of depression. *European Neuropsychopharmacology*. 2013 2013;23:S339. doi:10.1016/S0924-977X(13)70533-9 | 2 |
| Kadam UT, Thomas E, Croft PR. Is chronic widespread pain a predictor of all-cause morbidity? A 3 year prospective population based study in family practice. *J Rheumatol*. 2005-7 2005;32(7):1341-8. doi: | 6 |
| Kaila-Kangas L, Haukka E, Miranda H, et al. Co-occurrence of chronic musculoskeletal diseases and common mental disorders as a predictor of work disability in Finland. *European Journal of Epidemiology*. 2013 2013;28(1):S128. doi:10.1007/s10654-013-9820-0 | 1 |
| Kaila-Kangas L, Haukka E, Miranda H, et al. Common mental and musculoskeletal disorders as predictors of disability retirement among Finns. *Journal of Affective Disorders*. 2014 2014;165:38-44. doi:10.1016/j.jad.2014.04.036 | 1 |
| Karatel M, Bulut ZI, Sari EO, Pelin Z, Yakut Y. The profile of musculoskeletal pain and its associations with sleep quality and depression during the COVID-19 in Turkey. *Korean Journal of Pain*. 2022 2022;35(1):78-85. doi:10.3344/kjp.2022.35.1.78 | 2 |
| Kelleher C, Doyle F, Conroy R, Hickey A. Does pain mediate the relationship between physical activity and depression in older adults? Findings from two nationally representative studies. *Irish Journal of Medical Science*. 2013 2013;182:S285. doi:10.1007/s11845-013-0985-z | 1 |
| Kempen GI, Miedema I, van den Bos GA, Ormel J. Relationship of domain-specific measures of health to perceived overall health among older subjects. *J Clin Epidemiol*. 1998-1 1998;51(1):11-8. doi:10.1016/s0895-4356(97)00234-5 | 7 |
| Keshavarzi S, Ahmadi SM, Lankarani KB. The impact of depression and malnutrition on health-related quality of life among the elderly Iranians. *Glob J Health Sci*. 2014-11-26 2014;7(3):161-70. doi:10.5539/gjhs.v7n3p161 | 7 |
| Kim DJ. Factors affecting depression and health-related quality of life in the elderly during the COVID-19 pandemic. *Osong Public Health Res Perspect*. 2023-12 2023;14(6):520-529. doi:10.24171/j.phrp.2023.0166 | 6 |
| Kim S, Lee J, Boone D. Protective and Risk Factors at the Intersection of Chronic Pain, Depression, Anxiety, and Somatic Amplification: A Latent Profile Approach. *Journal of Pain Research*. 2022 2022;15:1107-1121. doi:10.2147/JPR.S340382 | 7 |
| Kishi T, Matsuda Y, Mukai T, et al. A cross-sectional survey to investigate the prevalence of pain in Japanese patients with major depressive disorder and schizophrenia. *Compr Psychiatry*. 2015-5 2015;59:91-7. doi:10.1016/j.comppsych.2015.02.004 | 2 |
| Kobayashi-Gutiérrez A, Martinez-Bonilla G, Bernard-Medina AG, et al. Depression and its correlation with in patients pain in the rheumatology service of a Mexican teaching hospital. *Rheumatol Int*. 2009-8 2009;29(10):1169-75. doi:10.1007/s00296-008-0834-y | 2 |
| Kohler IV, Ciancio A, Kämpfen F, et al. Pain Is Widespread and Predicts Poor Mental Health Among Older Adults in Rural Malawi. *Innov Aging*. 2022 2022;6(3):igac008. doi:10.1093/geroni/igac008 | 7 |
| Koren Y, Leveille SG, You T. Brief Pain Inventory Pain Interference Subscale: Assessing Interference With Daily Living Activities in Older Adults With Multisite Musculoskeletal Pain. *Front Pain Res (Lausanne)*. 2022 2022;3:897725. doi:10.3389/fpain.2022.897725 | 5 |
| Kostadinovic M, Nikolic D, Cirovic D, et al. Health predictors of pain in elderly-a Serbian population-based study. *Diagnostics*. 2019 2019;9(2) doi:10.3390/diagnostics9020047 | 5 |
| Kotwal A, Waite L, Dale W, Perissinotto CM, Covinsky K, Smith AK. Pain and loneliness: Results of a nationally-representative survey of community-dwelling older adults. *Journal of the American Geriatrics Society*. 2019 2019;67:S54-S55. doi:10.1111/jgs.15898 | 1 |
| Kovacs D, Eszlari N, Petschner P, et al. Interleukin-6 promoter polymorphism interacts with pain and life stress influencing depression phenotypes. *J Neural Transm (Vienna)*. 2016-5 2016;123(5):541-8. doi:10.1007/s00702-016-1506-9 | 5 |
| Koyama F, Yoda T, Hirao T. Insomnia and depression: Japanese hospital workers questionnaire survey. *Open Medicine (Poland)*. 2017 2017;12(1):391-398. doi:10.1515/med-2017-0056 | 2 |
| Koyanagi A, Smith L, Shin JI, et al. Multimorbidity and subjective cognitive complaints: Findings from 48 low- and middle-income countries of the World Health Survey 2002–2004. *Journal of Alzheimer's Disease*. 2021 2021;81(4):1737-1747. doi:10.3233/JAD-201592 | 5 |
| Krawczyk P. Depressive disorders at general practitioner's office - Diagnostics and pharmacotherapy. *Pediatria i Medycyna Rodzinna*. 2015 2015;10(2):174-189. doi:10.15557/PiMR.2014.0021 | 1 |
| Kroenke K. Patients presenting with somatic complaints: epidemiology, psychiatric comorbidity and management. *Int J Methods Psychiatr Res*. 2003 2003;12(1):34-43. doi:10.1002/mpr.140 | 1 |
| Krogstad H, Loge JH, Grotmol KS, et al. Symptoms in the general Norwegian adult population - prevalence and associated factors. *BMC Public Health*. 2020-6-23 2020;20(1):988. doi:10.1186/s12889-020-09109-2 | 7 |
| Kruger TM, Abner EL, Mendiondo M, Schmitt FA, Smith CD, Jicha GA. Differential reports of pain and depression differentiate mild cognitive impairment from cognitively intact elderly participants. *J Geriatr Psychiatry Neurol*. 2012-6 2012;25(2):107-12. doi:10.1177/0891988712445097 | 2 |
| Kurlowicz LH, Outlaw FH, Ratcliffe SJ, Evans LK. An Exploratory Study of Depression Among Older African American Users of an Academic Outpatient Rehabilitation Program. *Archives of Psychiatric Nursing*. 2005 2005;19(1):3-9. doi:10.1016/j.apnu.2004.11.001 | 2 |
| Lacasse A, Choinière M, Connelly JA. Knowledge, beliefs, and attitudes of the Quebec population toward chronic pain: Where are we now? *Can J Pain*. 2017 2017;1(1):151-160. doi:10.1080/24740527.2017.1369849 | 4 |
| Laguardia J, Campos MR, Travassos CM, Najar AL, Anjos LA, Vasconcellos MM. Psychometric evaluation of the SF-36 (v.2) questionnaire in a probability sample of Brazilian households: results of the survey Pesquisa Dimensões Sociais das Desigualdades (PDSD), Brazil, 2008. *Health Qual Life Outcomes*. 2011-8-3 2011;9:61. doi:10.1186/1477-7525-9-61 | 7 |
| Lam CLK, Guo VY, Yu EYT, Fung CSC, Wong CKH, Tiwari AFY. Changes in health-related quality of life among adults from low-income families in Hong Kong. *Quality of Life Research*. 2016 2016;25(1):193-194. doi:10.1007/s11136-016-1390-7 | 1 |
| Landi F, Onder G, Cesari M, Russo A, Barillaro C, Bernabei R. Pain and its relation to depressive symptoms in frail older people living in the community: an observational study. *J Pain Symptom Manage*. 2005-3 2005;29(3):255-62. doi:10.1016/j.jpainsymman.2004.06.016 | 2 |
| Landmark T, Dale O, Romundstad P, Woodhouse A, Kaasa S, Borchgrevink PC. Development and course of chronic pain over 4 years in the general population: The HUNT pain study. *Eur J Pain*. 2018-10 2018;22(9):1606-1616. doi:10.1002/ejp.1243 | 6 |
| Lang-Illievich K, Rumpold-Seitlinger G, Szilagyi IS, et al. Biological, psychological, and social factors associated with worsening of chronic pain during the first wave of the COVID-19 pandemic: a cross-sectional survey. *British Journal of Anaesthesia*. 2021 2021;127(1):e37-e39. doi:10.1016/j.bja.2021.04.010 | 4 |
| Langley PC, Van Litsenburg C, Cappelleri JC, Carroll D. The burden associated with neuropathic pain in Western Europe. *J Med Econ*. 2013 2013;16(1):85-95. doi:10.3111/13696998.2012.729548 | 5 |
| Larsson B, Gerdle B, Bernfort L, Levin L, Dragioti E. Distinctive subgroups derived by cluster analysis based on pain and psychological symptoms in Swedish older adults with chronic pain - a population study (PainS65+). *BMC Geriatr*. 2017-9-2 2017;17(1):200. doi:10.1186/s12877-017-0591-4 | 2 |
| Laskawska MS, Hébert HL, Richardson C, et al. The core minimum dataset for measuring pain outcomes in pain services across Scotland. Developing and testing a brief multi-dimensional questionnaire. *Br J Pain*. 2022-10 2022;16(5):504-517. doi:10.1177/20494637221092907 | 2 |
| Lau JH, Lee ES, Zhang Y, et al. Association between self-reported sedentary behavior and health-related quality of life among multimorbidity patients in Singapore. *American Journal of Health Promotion*. 2021 2021;35(7):929-938. doi:10.1177/08901171211001274 | 2 |
| Lawson KD, Mercer SW, Wyke S, et al. Double trouble: The impact of multimorbidity and deprivation on preference-weighted health related quality of life a cross sectional analysis of the Scottish Health Survey. *International Journal for Equity in Health*. 2013 2013;12(1). doi:10.1186/1475-9276-12-67 | 5 |
| Lazarevič P, Brandt M. Diverging ideas of health? Comparing the basis of health ratings across gender, age, and country. *Social Science and Medicine*. 2020 2020;267 . doi:10.1016/j.socscimed.2020.112913 | 7 |
| Lee HS, Lee S, Park S, et al. The association between somatic and psychological discomfort and health-related quality of life according to the elderly and non-elderly. *Quality of Life Research*. 2018 2018;27(3):673-681. doi:10.1007/s11136-017-1715-1 | 2 |
| Lee S, Guo WJ, Tsang A, et al. Impaired role functioning and treatment rates for mental disorders and chronic physical disorders in metropolitan China. *Psychosomatic Medicine*. 2009 2009;71(8):886-893. doi:10.1097/PSY.0b013e3181baa65e | 7 |
| Lee S, Tsang A. A population-based study of depression and three kinds of frequent pain conditions and depression in Hong Kong. *Pain Med*. 2009-1 2009;10(1):155-63. doi:10.1111/j.1526-4637.2008.00541.x | 5 |
| Lee W, Hong K, Lim SS, Kim DH, Yoon JH. The Association Between Symptomatic and Diagnostic Depression and Pain Among the Elderly Population in South Korea. *J Nerv Ment Dis*. 2017-9 2017;205(9):699-704. doi:10.1097/nmd.0000000000000633 | 2 |
| Leggett A, Zarit SH, Nguyen NH, Hoang CN, Nguyen HT. The influence of social factors and health on depressive symptoms and worry: a study of older Vietnamese adults. *Aging Ment Health*. 2012 2012;16(6):780-6. doi:10.1080/13607863.2012.667780 | 7 |
| Leibson CL, Garrard J, Nitz N, et al. The role of depression in the association between self-rated physical health and clinically defined illness. *The Gerontologist*. 1999 1999;39(3):291-298. doi:10.1093/geront/39.3.291 | 2 |
| Leiknes KA, Finset A, Moum T, Sandanger I. Course and predictors of medically unexplained pain symptoms in the general population. *J Psychosom Res*. 2007-2 2007;62(2):119-28. doi:10.1016/j.jpsychores.2006.08.009 | 5 |
| Leone M, Kuja-Halkola R, Leval A, et al. Association of Youth Depression with Subsequent Somatic Diseases and Premature Death. *JAMA Psychiatry*. 2021 2021;78(3):302-310. doi:10.1001/jamapsychiatry.2020.3786 | 2 |
| Leung YY, Teo SL, Chua MB, Raman P, Liu C, Chan A. Living arrangements, social networks and onset or progression of pain among older adults in Singapore. *Geriatrics and Gerontology International*. 2016 2016;16(6):693-700. doi:10.1111/ggi.12539 | 6 |
| Levinson D, Karger CJ, Haklai Z. Chronic physical conditions and use of health services among persons with mental disorders--Results from the Israel National Health Survey. *General Hospital Psychiatry*. 2008 2008;30(3):226-232. doi:10.1016/j.genhosppsych.2008.02.007 | 6 |
| Li HC, Zhang MY, Wang G, et al. Association between painful physical symptoms and clinical outcomes in Chinese patients with major depressive disorder: a three-month observational study. *Chin Med J (Engl)*. 2010-8-5 2010;123(15):2063-9. doi:10.3760/cma.j.issn.0366-6999.2010.15.020 | 2 |
| Li W, Zhao N, Yan X, et al. The prevalence of depressive and anxiety symptoms and their associations with quality of life among clinically stable older patients with psychiatric disorders during the COVID-19 pandemic. *Transl Psychiatry*. 2021-1-26 2021;11(1):75. doi:10.1038/s41398-021-01196-y | 2 |
| Liu C, Qin X, Kang M, et al. Assessing the association of smoking and alcohol consumption with chronic pain in depression and anxiety disorders. *International Journal of Mental Health and Addiction*. 2024 2024;doi:10.1007/s11469-024-01357-2 | 5 |
| Liu M, Hou T, Nkimbeng M, et al. Associations between symptoms of pain, insomnia and depression, and frailty in older adults: A cross-sectional analysis of a cohort study. *Int J Nurs Stud*. 2021-5 2021;117:103873. doi:10.1016/j.ijnurstu.2021.103873 | 2 |
| Liu XK, Xiao SY, Zhou L, Hu M, Zhou W, Liu HM. Sleep quality and covariates as predictors of pain intensity among the general population in rural China. *J Pain Res*. 2018 2018;11:857-866. doi:10.2147/jpr.S156731 | 7 |
| Lockett H, Jury A, Tuason C, Lai J, Fergusson D. Comorbidities between mental and physical health problems: An analysis of the New Zealand Health Survey data. *New Zealand Journal of Psychology*. 2018 2018;47(3):5-11. | 4 |
| LoMartire R, Äng BO, Gerdle B, Vixner L. Psychometric properties of Short Form-36 Health Survey, EuroQol 5-dimensions, and Hospital Anxiety and Depression Scale in patients with chronic pain. *Pain*. 2020-1 2020;161(1):83-95. doi:10.1097/j.pain.0000000000001700 | 2 |
| Lourenc¸o S, Lucas R, Ramos E, Barros H. Association between individual psychosocial context and early chronic musculoskeletal pain in young adults. *Annals of the Rheumatic Diseases*. 2013 2013;72. doi:10.1136/annrheumdis-2013-eular.2284 | 1 |
| Lu L, Shen H, Tan L, et al. Prevalence and factors associated with anxiety and depression among community-dwelling older adults in Hunan, China: a cross-sectional study. *BMC Psychiatry*. 2023 2023;23(1). doi:10.1186/s12888-023-04583-5 | 4 |
| Lucena L, Lavigne G, Fabbro CD, Andersen ML, Tufik S, Hachul H. Association between night pain and quality of life in women: A general population sleep study. *Eur J Pain*. 2023-3 2023;27(3):401-412. doi:10.1002/ejp.2066 | 5 |
| Macfarlane GJ, Pye SR, Finn JD, et al. Investigating the determinants of international differences in the prevalence of chronic widespread pain: Evidence from the European Male Ageing Study. *Annals of the Rheumatic Diseases*. 2009 2009;68(5):690-695. doi:10.1136/ard.2008.089417 | 5 |
| Madhukar H. Trivedi MWJK, MD ; Ella Daly, MB, MRCPsych ; Anita H. Clayton, MD ; Ellen Frank, PhD. Recent advances in the treatment of depression in the presence of physical symptoms. *J Clin Psychiatry*. 2006-2 2006;67(2):310-21. doi:10.4088/jcp.v67n0220 | 1 |
| Magni G, Marchetti M, Moreschi C, Merskey H, Luchini SR. Chronic musculoskeletal pain and depressive symptoms in the National Health and Nutrition Examination. I. Epidemiologic Follow-up Study. *Pain*. 1993 1993;53(2):163-168. doi:10.1016/0304-3959(93)90076-2 | 5 |
| Magni G, Moreschi C, Rigatti-Luchini S, Merskey H. Prospective study on the relationship between depressive symptoms and chronic musculoskeletal pain. *Pain*. 1994-3 1994;56(3):289-297. doi:10.1016/0304-3959(94)90167-8 | 5 |
| Mall S, Lund C, Vilagut G, Alonso J, Williams DR, Stein DJ. Days out of role due to mental and physical illness in the South African stress and health study. *Soc Psychiatry Psychiatr Epidemiol*. 2015-3 2015;50(3):461-8. doi:10.1007/s00127-014-0941-x | 7 |
| Marcuzzi A, Skarpsno ES, Nilsen TIL, Mork PJ. The interplay between multisite pain and insomnia on the risk of anxiety and depression: the HUNT study. *BMC Psychiatry*. 2022-2-16 2022;22(1):124. doi:10.1186/s12888-022-03762-0 | 4 |
| Marrie RA, Patten S, Greenfield J, et al. Physical comorbidities are associated with an increased risk of psychiatric comorbidity in multiple sclerosis. *Neurology*. 2016 2016;86(16). doi:10.1212/WNL.86.16_supplement.S37.004 | 2 |
| Marsman A, Pries L-K, ten Have M, et al. Do current measures of polygenic risk for mental disorders contribute to population variance in mental health? *Schizophrenia Bulletin*. 2020 2020;46(6):1353-1362. doi:10.1093/schbul/sbaa086 | 6 |
| Martín Lascuevas P, Ballina García FJ, Hernandez Mejía R, Cueto Espinar A. [Prevalence of localized pain of the locomotor system in the general population]. *Aten Primaria*. 1995-4-30 1995;15(7):449-51. | 5 |
| Martínez NT, Gómez-Restrepo C, Ramírez S, Rodríguez MN. [Prevalence of Mood and Anxiety Disorders on People with Chronic Conditions. Results from the National Mental Health Survey in Colombia 2015]. *Rev Colomb Psiquiatr*. 2016-12 2016;45:141-146. doi:10.1016/j.rcp.2016.06.001 | 4 |
| Marttinen MK, Kautiainen H, Haanpää M, et al. Low socio-economic status, obesity and depression independently associated with pain in the elderly - A Finnish population based study. *Scandinavian Journal of Pain*. 2018 2018;18:S9-S10. doi:10.1515/sjpain-2018-9902 | 1 |
| Mathew AJ, Chopra A, Thekkemuriyil DV, George E, Goyal V, Nair JB. Impact of musculoskeletal pain on physical function and health-related quality of life in a rural community in south India: a WHO-ILAR-COPCORD-BJD India study. *Clin Rheumatol*. 2011-11 2011;30(11):1491-7. doi:10.1007/s10067-011-1824-0 | 4 |
| Mathur A, Graham-Engeland JE, Sliwinski MJ, Smyth JM, Lipton RB, Katz MJ. Psychological vulnerabilities explain an association between chronic life stress and pain: The role of resilience resources. *Psychosomatic Medicine*. 2016 2016;78(3):A63. doi:10.1097/PSY.0000000000000343 | 1 |
| McBeth J, Lacey RJ, Wilkie R. Predictors of new-onset widespread pain in older adults: results from a population-based prospective cohort study in the UK. *Arthritis Rheumatol*. 2014-3 2014;66(3):757-67. doi:10.1002/art.38284 | 5 |
| McBeth J, Tomenson B, Chew-Graham CA, et al. Common and unique associated factors for medically unexplained chronic widespread pain and chronic fatigue. *Journal of Psychosomatic Research*. 2015 2015;79(6):484-491. doi:10.1016/j.jpsychores.2015.10.004 | 5 |
| McIntosh AM, Hall LS, Zeng Y, et al. Genetic and Environmental Risk for Chronic Pain and the Contribution of Risk Variants for Major Depressive Disorder: A Family-Based Mixed-Model Analysis. *PLoS Med*. 2016-8 2016;13(8):e1002090. doi:10.1371/journal.pmed.1002090 | 6 |
| McWilliams LA, Cox BJ, Enns MW. Mood and anxiety disorders associated with chronic pain: an examination in a nationally representative sample. *Pain*. 2003-11 2003;106(1):127-33. doi:10.1016/s0304-3959(03)00301-4 | 5 |
| Meana M, Cho R, DesMeules M. Chronic Pain: The Extra Burden on Canadian Women. *BMC Womens Health*. 2004-8-25 2004;4:S17. doi:10.1186/1472-6874-4-s1-s17 | 5 |
| Means-Christensen AJ, Roy-Byrne PP, Sherbourne CD, Craske MG, Stein MB. Relationships among pain, anxiety, and depression in primary care. *Depress Anxiety*. 2008 2008;25(7):593-600. doi:10.1002/da.20342 | 2 |
| Milani SA, Howrey B, Rodriguez MA, Samper-Ternent R, Wong R. Gender differences in activity-limiting pain trajectories over a 17-year period in the Mexican Health and Aging Study. *Pain*. 2022-2-1 2022;163(2):e285-e292. doi:10.1097/j.pain.0000000000002292 | 2 |
| Miró J, Sánchez-Rodríguez E, Nolla MC, Costa RM, Pais-Ribeiro J, Ferreira-Valente A. The Role of Resilience, Happiness, and Social Support in the Psychological Function during the Late Stages of the Lockdown in Individuals with and without Chronic Pain. *Int J Environ Res Public Health*. 2022-5-31 2022;19(11)doi:10.3390/ijerph19116708 | 7 |
| Mobily PR, Herr KA, Clark MK, Wallace RB. An epidemiologic analysis of pain in the elderly: The Iowa 65+ Rural Health Study. *Journal of Aging and Health*. 1994 1994;6(2):139-154. Doi:10.1177/089826439400600201 | 4 |
| Morales M, Ingram M, Coulter KM, et al. Factors Associated with Depressive Symptoms among Mexican-Origin Adults in a Community Sample at the US Mexico Border Region. *Int J Environ Res Public Health*. 2023-5-31 2023;20(11)doi:10.3390/ijerph20116017 | 7 |
| Mossey J, Gallagher RM. Longitudinal experience of a nationally representative sample of the u.s. population 18 years and older: The importance of co-morbid major depression, alcohol and drug abuse. *Pain Medicine*. 2009 2009;10(1):263-264. doi:10.1111/j.1526-4637.2008.00537.x | 1 |
| Munce SE, Weller I, Robertson Blackmore EK, Heinmaa M, Katz J, Stewart DE. The role of work stress as a moderating variable in the chronic pain and depression association. *J Psychosom Res*. 2006-11 2006;61(5):653-60. doi:10.1016/j.jpsychores.2006.03.048 | 5 |
| Mundal I, Bjørngaard JH, Nilsen TIL, Nicholl BI, Gråwe RW, Fors EA. Long-term changes in musculoskeletal pain sites in the general population: The HUNT study. *The Journal of Pain*. 2016 2016;17(11):1246-1256. doi:10.1016/j.jpain.2016.08.006 | 5 |
| Mundal I, Gråwe RW, Bjørngaard JH, Linaker OM, Fors EA. Prevalence and long-term predictors of persistent chronic widespread pain in the general population in an 11-year prospective study: the HUNT study. *BMC Musculoskelet Disord*. 2014-6-20 2014;15:213. doi:10.1186/1471-2474-15-213 | 5 |
| Mundal I, Gråwe RW, Bjørngaard JH, Linaker OM, Fors EA. Psychosocial factors and risk of chronic widespread pain: An 11-year follow-up study - The HUNT study. *Pain*. 2014 2014;155(8):1555-1561. doi:10.1016/j.pain.2014.04.033 | 5 |
| Mundal IP, Fors EA, Lydersen S, Gra˚we RW. Predictors of long-term outcomes in chronic widespread pain in the general population: An 11 years prospective epidemiologic study. *Psychosomatic Medicine*. 2013 2013;75(3):A51-A52. doi:10.1097/01.psy.0000429452.16135.1b | 1 |
| Nakao M, Yano E. Somatic symptoms for predicting depression: one-year follow-up study in annual health examinations. *Psychiatry Clin Neurosci*. 2006-4 2006;60(2):219-25. doi:10.1111/j.1440-1819.2006.01489.x | 2 |
| Nazar G, Gatica-Saavedra M, Provoste A, et al. [Factors associated with depression in Chilean adults. Results of the 2016-2017 National Health Survey]. *Rev Med Chil*. 2021-10 2021;149(10):1430-1439. doi:10.4067/s0034-98872021001001430 | 4 |
| Niklasson J, Näsman M, Nyqvist F, et al. Higher morale is associated with lower risk of depressive disorders five years later among very old people. *Archives of Gerontology and Geriatrics*. 2017 2017;69:61-68. doi:10.1016/j.archger.2016.11.008 | 5 |
| Nordström A, Bodlund O. Every third patient in primary care suffers from depression, anxiety or alcohol problems. *Nord J Psychiatry*. 2008 2008;62(3):250-5. doi:10.1080/08039480802141129 | 6 |
| Novick D, Shi Q, Yue L, Moneta MV, Siddi S, Haro JM. Impact of pain and remission in the functioning of patients with depression in Mainland China, Taiwan, and Hong Kong. *Asia Pac Psychiatry*. 2018-6 2018;10(2):e12295. doi:10.1111/appy.12295 | 2 |
| Ohayon MM, Roberts LW. Links between occupational activities and depressive mood in young adult populations. *J Psychiatr Res*. 2014-2 2014;49:10-7. doi:10.1016/j.jpsychires.2013.10.002 | 2 |
| Ohayon MM, Stingl JC. Prevalence and comorbidity of chronic pain in the German general population. *J Psychiatr Res*. 2012-4 2012;46(4):444-50. doi:10.1016/j.jpsychires.2012.01.001 | 7 |
| Ojagbemi A, Bello T, Luo Z, Gureje O. Chronic Conditions, New Onset, and Persistent Disability in the Ibadan Study of Aging. *J Gerontol A Biol Sci Med Sci*. 2017-7-1 2017;72(7):997-1005. doi:10.1093/gerona/glv188 | 7 |
| Olsen LR, Mortensen EL, Bech P. Prevalence of major depression and stress indicators in the Danish general population. *Acta Psychiatr Scand*. 2004-2 2004;109(2):96-103. doi:10.1046/j.0001-690x.2003.00231.x | 7 |
| Olsson M, Björkelund AJ, Sandberg J, et al. Factors important for health-related quality of life in men and women: The population based SCAPIS study. *PLoS One*. 2023 2023;18(11):e0294030. doi:10.1371/journal.pone.0294030 | 7 |
| Onder G, Landi F, Gambassi G, et al. Association between pain and depression among older adults in Europe: results from the Aged in Home Care (AdHOC) project: a cross-sectional study. *J Clin Psychiatry*. 2005-8 2005;66(8):982-8. doi:10.4088/jcp.v66n0804 | 2 |
| Ozminkowski RJ, Musich S, Bottone FG, Jr., et al. The burden of depressive symptoms and various chronic conditions and health concerns on the quality of life among those with Medicare Supplement Insurance. *Int J Geriatr Psychiatry*. 2012-9 2012;27(9):948-58. doi:10.1002/gps.2806 | 5 |
| Pakriev S, Vasar V, Aluoja A, Saarma M, Shlik J. Prevalence of mood disorders in the rural population of Udmurtia. *Acta Psychiatr Scand*. 1998-3 1998;97(3):169-74. doi:10.1111/j.1600-0447.1998.tb09983.x | 4 |
| Pap D, Xenia G, Lazary J, Benko A, Molnar E, Bagdy GY. The association of pain vigilance and awareness with subclinical depression. *European Neuropsychopharmacology*. 2009 2009;19:S396. doi:10.1016/S0924-977X(09)70610-8 | 1 |
| Park M. Comparing comorbidity of chronic pain and depression between Asian Americans and European Americans. *European Psychiatry*. 2019 2019;56:S55. doi:10.1016/j.eurpsy.2019.01.003 | 1 |
| Park MJ, Choi KW, Na EJ, et al. Multiple types of somatic pain increase suicide attempts and depression: A nationwide community sample of Korean adults. *Compr Psychiatry*. 2019-4 2019;90:43-48. doi:10.1016/j.comppsych.2018.12.006 | 5 |
| Patel K, Turk D. Evaluation of pain intensity ratings among older adults with and without dementia: Findings from the national health and aging trends study. *Journal of Pain*. 2016 2016;17(4):S8. doi:10.1016/j.jpain.2016.01.034 | 1 |
| Patten SB. Long-term medical conditions and major depression in a Canadian population study at waves 1 and 2. *J Affect Disord*. 2001-3 2001;63(1):35-41. doi:10.1016/s0165-0327(00)00186-5 | 5 |
| Patten SB, Williams JVA, Lavorato DH, Bulloch AGM, Currie G, Emery H. Depression and painful conditions: Patterns of association with health status and health utility ratings in the general population. *Quality of Life Research: An International Journal of Quality of Life Aspects of Treatment, Care & Rehabilitation*. 2014 2014;23(1):363-371. doi:10.1007/s11136-013-0449-y | 5 |
| Patten SB, Williams JVA, Lavorato DH, Modgill G, Jetté N, Eliasziw M. Major depression as a risk factor for chronic disease incidence: Longitudinal analyses in a general population cohort. *General Hospital Psychiatry*. 2008 2008;30(5):407-413. doi:10.1016/j.genhosppsych.2008.05.001 | 5 |
| Patten SB, Williams JVA, Lavorato DH, et al. Patterns of association of chronic medical conditions and major depression. *Epidemiol Psychiatr Sci*. 2018-2 2018;27(1):42-50. doi:10.1017/s204579601600072x | 5 |
| Paulus DJ, Bakhshaie J, Garza M, et al. Pain severity and emotion dysregulation among Latinos in a community health care setting: relations to mental health. *Gen Hosp Psychiatry*. 2016-9 2016;42:41-8. doi:10.1016/j.genhosppsych.2016.07.002 | 7 |
| Pavlovic J, Wang C, Katz M, Derby C, Zimmerman M, Lipton R. Pain prevalence in the elderly. *Neurology*. 2015 2015;84. doi:10.1212/WNL.84.14_supplement.P3.300 | 1 |
| Pengpid S, Peltzer K. Self-rated physical and mental health among older adults 80 years and older: cross-sectional results from a National community sample in Thailand. *BMC Public Health*. 2023-7-7 2023;23(1):1304. doi:10.1186/s12889-023-16237-y | 7 |
| Pereira FG, França MH, Paiva MCA, Andrade LH, Viana MC. Prevalence and clinical profile of chronic pain and its association with mental disorders. *Rev Saude Publica*. 2017-11-17 2017;51:96. doi:10.11606/s1518-8787.2017051007025 | 6 |
| Pinto-Meza A, Fernández A, Fullana MA, et al. Impact of mental disorders and chronic physical conditions in health-related quality of life among primary care patients: results from an epidemiological study. *Qual Life Res*. 2009-10 2009;18(8):1011-8. doi:10.1007/s11136-009-9522-y | 5 |
| Polenick CA, Brooks JM, Birditt KS. Own and partner pain intensity in older couples: Longitudinal effects on depressive symptoms. *Pain*. 2017 2017;158(8):1546-1553. doi:10.1097/j.pain.0000000000000949 | 2 |
| Qadeer RA, Shanahan L, Ferro MA. Chronic disruptive pain in emerging adults with and without chronic health conditions and the moderating role of psychiatric disorders: Evidence from a population-based cross-sectional survey in Canada. *Scandinavian Journal of Pain*. 2017 2017;17:30-36. doi:10.1016/j.sjpain.2017.07.009 | 6 |
| Qasem N, Al-Ali MB, Sanwar MB, Aquily MQ. Psychological impact and mental health effect of COVID-19 among Jordanian population. *Jordan Medical Journal*. 2022 2022;56(1):1-16. doi:10.35516/jmj.v56i1.218 | 4 |
| Quiton RL, Leibel DK, Boyd EL, Waldstein SR, Evans MK, Zonderman AB. Sociodemographic patterns of pain in an urban community sample: an examination of intersectional effects of sex, race, age, and poverty status. *Pain*. 2020-5 2020;161(5):1044-1051. doi:10.1097/j.pain.0000000000001793 | 5 |
| Raftery MN, Sarma K, Murphy AW, De La Harpe D, Normand C, McGuire BE. Chronic pain in the Republic of Ireland - Community prevalence, psychosocial profile and predictors of pain-related disability: Results from the Prevalence, Impact and Cost of Chronic Pain (PRIME) study, Part 1. *Pain*. 2011 2011;152(5):1096-1103. doi:10.1016/j.pain.2011.01.019 | 6 |
| Ramage-Morin PL, Gilmour H. Chronic pain at ages 12 to 44. *Health Rep*. 2010-12 2010;21(4):53-61. | 6 |
| Rapti E, Damigos D, Apostolara P, Roka V, Tzavara C, Lionis C. Patients with chronic pain: evaluating depression and their quality of life in a single center study in Greece. *BMC Psychol*. 2019-12-21 2019;7(1):86. doi:10.1186/s40359-019-0366-0 | 2 |
| Rashiq S, Dick BD. Factors associated with chronic noncancer pain in the Canadian population. *Pain Res Manag*. 2009-11 2009;14(6):454-60. doi:10.1155/2009/919628 | 6 |
| Ray BM, Kelleran KJ, Fodero JG, Harvell-Bowman LA. Examining the Relationship Between Chronic Pain and Mortality in U.S. Adults. *J Pain*. 2024-10 2024;25(10):104620. doi:10.1016/j.jpain.2024.104620 | 6 |
| Reeves WC, Lin JM, Nater UM. Mental illness in metropolitan, urban and rural Georgia populations. *BMC Public Health*. 2013-4-30 2013;13:414. doi:10.1186/1471-2458-13-414 | 4 |
| Réthelyi JM, Berghammer R, Ittzés A, Szumska I, Purebl G, Csoboth C. Comorbidity of pain problems and depressive symptoms in young women: results from a cross-sectional survey among women aged 15-24 in Hungary. *Eur J Pain*. 2004-2 2004;8(1):63-9. doi:10.1016/s1090-3801(03)00074-0 | 5 |
| Réthelyi JM, Berghammer R, Kopp MS. Comorbidity of pain-associated disability and depressive symptoms in connection with sociodemographic variables: results from a cross-sectional epidemiological survey in Hungary. *Pain*. 2001-8 2001;93(2):115-121. doi:10.1016/s0304-3959(01)00301-3 | 5 |
| Robles E, Angelone C, Ondé D, Vázquez C. Somatic symptoms in the general population of Spain: Validation and normative data of the Patient Health Questionnaire-15 (PHQ-15). *Journal of Affective Disorders*. 2024 2024;362:762-771. doi:10.1016/j.jad.2024.07.087 | 7 |
| Roizenblatt S, Souza AL, Palombini L, Godoy LM, Tufik S, Bittencourt LR. Musculoskeletal Pain as a Marker of Health Quality. Findings from the Epidemiological Sleep Study among the Adult Population of São Paulo City. *PLoS One*. 2015 2015;10(11):e0142726. doi:10.1371/journal.pone.0142726 | 5 |
| Rosa JSDL, Brady BR, Ibrahim MM, et al. Chronic Pain is Experienced by the Majority of U.S. Adults with Unremitted Anxiety or Depression. *Journal of Pain*. 2024 2024;25(4):44. doi:10.1016/j.jpain.2024.01.204 | 1 |
| Rosso AL, Gallagher RM, Luborsky M, Mossey JM. Depression and self-rated health are proximal predictors of episodes of sustained change in pain in independently living, community dwelling elders. *Pain Medicine*. 2008 2008;9(8):1035-1049. doi:10.1111/j.1526-4637.2008.00533.x | 7 |
| Rothrock NE, Hays RD, Spritzer K, Yount SE, Riley W, Cella D. Relative to the general US population, chronic diseases are associated with poorer health-related quality of life as measured by the Patient-Reported Outcomes Measurement Information System (PROMIS). *Journal of Clinical Epidemiology*. 2010 2010;63(11):1195-1204. doi:10.1016/j.jclinepi.2010.04.012 | 4 |
| Rottenberg Y, Jacobs JM, Stessman J. Depression and health service utilization from age 70 to 85: the Jerusalem Longitudinal Study. *J Am Med Dir Assoc*. 2013-9 2013;14(9):711.e1-6. doi:10.1016/j.jamda.2013.06.001 | 5 |
| Rottenberg Y, Jacobs JM, Stessman J. Prevalence of pain with advancing age brief report. *J Am Med Dir Assoc*. 2015-3 2015;16(3):264.e1-5. doi:10.1016/j.jamda.2014.12.006 | 5 |
| Royall DR, Salazar R, Palmer RF. Latent variables may be useful in pain's assessment. *Health Qual Life Outcomes*. 2014-1-30 2014;12:13. doi:10.1186/1477-7525-12-13 | 7 |
| Roystonn K, Koh YS, Shafie S, et al. Understanding Major Depressive Disorder in Singapore: Insights from the second Singapore Mental Health Study (SMHS 2016). *J Affect Disord*. 2024-11-1 2024;364:295-304. doi:10.1016/j.jad.2024.08.046 | 5 |
| Rzewuska M, De Azevedo-Marques JM, Coxon D, et al. Epidemiology of multimorbidity within the Brazilian adult general population: Evidence from the 2013 National Health Survey (PNS 2013). *PLoS ONE*. 2017 2017;12(2). doi:10.1371/journal.pone.0171813 | 5 |
| Rzewuska M, Mallen CD, Strauss VY, Belcher J, Peat G. One-year trajectories of depression and anxiety symptoms in older patients presenting in general practice with musculoskeletal pain: A latent class growth analysis. *J Psychosom Res*. 2015-9 2015;79(3):195-201. doi:10.1016/j.jpsychores.2015.05.016 | 2 |
| Saastamoinen P, Laaksonen M, Kääriä SM, et al. Pain and disability retirement: A prospective cohort study. *Pain*. 2012 2012;153(3):526-531. doi:10.1016/j.pain.2011.11.005 | 2 |
| Sanders JB, Comijs HC, Bremmer MA, Deeg DJH, Beekman ATF. A 13-year prospective cohort study on the effects of aging and frailty on the depression-pain relationship in older adults. *International Journal of Geriatric Psychiatry*. 2015 2015;30(7):751-757. doi:10.1002/gps.4224 | 7 |
| Sanna L, Stuart AL, Pasco JA, et al. Physical comorbidities in men with mood and anxiety disorders: a population-based study. *BMC Med*. 2013-4-24 2013;11:110. doi:10.1186/1741-7015-11-110 | 4 |
| Santini ZI, Ekholm O, Koyanagi A, et al. Pain and associated functional impairment in the Danish general population: The role of mental well-being. *Journal of Public Mental Health*. 2023 2023;22(4):230-244. doi:10.1108/JPMH-07-2023-0065 | 7 |
| Sartorius N. Physical symptoms of depression as a public health concern. *J Clin Psychiatry*. 2003 2003;64:3-4. | 1 |
| Schaefert R, Höner C, Salm F, et al. Psychological and behavioral variables associated with the somatic symptom severity of general hospital outpatients in China. *Gen Hosp Psychiatry*. 2013-5 2013;35(3):297-303. doi:10.1016/j.genhosppsych.2012.11.001 | 5 |
| Schmitz N, Wang J, Malla A, Lesage A. Joint effect of depression and chronic conditions on disability: Results from a population-based study. *Psychosomatic Medicine*. 2007 2007;69(4):332-338. doi:10.1097/PSY.0b013e31804259e0 | 4 |
| Scott K, McGee MA, Schaaf D, Baxter J. Mental-physical comorbidity in an ethnically diverse population. *Soc Sci Med*. 2008-3 2008;66(5):1165-73. doi:10.1016/j.socscimed.2007.11.022 | 6 |
| Scott KM, Von Korff M, Alonso J, et al. Age patterns in the prevalence of DSM-IV depressive/anxiety disorders with and without physical co-morbidity. *Psychological Medicine*. 2008 2008;38(11):1659-1669. doi:10.1017/S0033291708003413 | 6 |
| Scott W, Jackson SE, Hackett RA. Perceived discrimination, health, and well-being among adults with and without pain: a prospective study. *Pain*. 2022-2-1 2022;163(2):258-266. doi:10.1097/j.pain.0000000000002368 | 7 |
| Selič P, Sv̌ab I, Rifel J, et al. The pattern of physical comorbidity and the psychosocial determinants of depression: A prospective cohort study on a representative sample of family practice attendees in Slovenia. *Mental Health in Family Medicine*. 2011 2011;8(3):147-155. | 4 |
| Sengupta S. Diagnosis of depression in general practice. *Indian Journal of Medical Sciences*. 2005 2005;59(5):217-225. doi:10.4103/0019-5359.16261 | 1 |
| Serra E. Depression in pain: Clinical aspects and therapy. *Douleurs*. 2014 2014;15(3):98-105. doi:10.1016/j.douler.2014.03.008 | 1 |
| Sharpe L, McDonald S, Correia H, et al. Pain severity predicts depressive symptoms over and above individual illnesses and multimorbidity in older adults. *BMC Psychiatry*. 2017-5-4 2017;17(1):166. doi:10.1186/s12888-017-1334-y | 2 |
| Sharpley CF, Bitsika V, Jesulola E, Agnew LL. Prevalence and structure of anxiety-depression in an Australian community sample. *Archives of Psychiatry and Psychotherapy*. 2016 2016;18(2):29-39. doi:10.12740/APP/62976 | 6 |
| She R, Yan Z, Jiang H, Vetrano DL, Lau JTF, Qiu C. Multimorbidity and Health-Related Quality of Life in Old Age: Role of Functional Dependence and Depressive Symptoms. *Journal of the American Medical Directors Association*. 2019 2019;20(9):1143-1149. doi:10.1016/j.jamda.2019.02.024 | 6 |
| Shega J, Andrew M, Herr K, Ersek M, Weiner D, Dale W. No differences in pain reported between older adults with and without Dementia. *Journal of the American Geriatrics Society*. 2012 2012;60:S237-S238. doi:10.1111/j.1532-5415.2012.04000.x | 1 |
| Shega J, Kotwal A, Weiner D, Dale W. The prevalence of pain and its relationship with health among a nationally representative sample of community-dwelling older adults in the united states: An analysis of the national social life, health, and aging project (nshap). *Journal of the American Geriatrics Society*. 2013 2013;61:S142. doi:10.1111/jgs.12263 | 1 |
| Shega JW, Paice JA, Rockwood K, Dale W. Is the presence of mild to moderate cognitive impairment associated with self-report of non-cancer pain? A cross-sectional analysis of a large population-based study. *J Pain Symptom Manage*. 2010-4 2010;39(4):734-42. doi:10.1016/j.jpainsymman.2009.09.016 | 6 |
| Shibata M, Ninomiya T, Jensen MP, et al. Alexithymia is associated with greater risk of chronic pain and negative affect and with lower life satisfaction in a general population: the Hisayama Study. *PLoS One*. 2014 2014;9(3):e90984. doi:10.1371/journal.pone.0090984 | 7 |
| Shiri R, Kaila-Kangas L, Ahola K, et al. The relation of co-occurring musculoskeletal pain and depressive symptoms with work ability. *J Occup Environ Med*. 2013-11 2013;55(11):1281-5. doi:10.1097/JOM.0b013e3182a4ffc4 | 5 |
| Silva AG, Queirós A, Cerqueira M, Rocha NP. Pain intensity is associated with both performance-based disability and self-reported disability in a sample of older adults attending primary health care centers. *Disabil Health J*. 2014-10 2014;7(4):457-65. doi:10.1016/j.dhjo.2014.05.001 | 7 |
| Silveira E, Skoog I, Sundh V, Allebeck P, Steen B. Health and well-being among 70-year-old migrants living in Sweden: Results from the H 70 gerontological and geriatric population studies in Göteborg. *Social Psychiatry and Psychiatric Epidemiology: The International Journal for Research in Social and Genetic Epidemiology and Mental Health Services*. 2002 2002;37(1):13-22. doi:10.1007/s127-002-8209-5 | 2 |
| Singh AN. Pain behaviours and psychiatric complications in pain syndrome. *International Medical Journal*. 2012 2012;19(3):205-207. | 1 |
| Skapinakis P, Rai D, Anagnostopoulos F, Harrison S, Araya R, Lewis G. Sleep disturbances and depressive symptoms: an investigation of their longitudinal association in a representative sample of the UK general population. *Psychol Med*. 2013-2 2013;43(2):329-39. doi:10.1017/s0033291712001055 | 5 |
| Sloane PD, Callahan L, Kahwati L, Mitchell CM. Development of a practice-based patient cohort for primary care research. *Family Medicine*. 2006 2006;38(1):50-58. | 5 |
| Smith DJ, Court H, McLean G, et al. Depression and multimorbidity: A cross-sectional study of 1,751,841 patients in primary care. *Journal of Clinical Psychiatry*. 2014 2014;75(11):1202-1208. doi:10.4088/JCP.14m09147 | 4 |
| Smyth R. Depression in physical illness. *Journal of the Royal College of Physicians of Edinburgh*. 2009 2009;39(4):337-342. doi:10.4997/JRCPE.2009.411 | 1 |
| Soh KC, Kua EH, Ng TP. Ethnic differences in depressive symptomatology among older persons in the Singapore longitudinal ageing studies (SLAS). *Annals of the Academy of Medicine Singapore*. 2014 2014;43(10):S33-S34. | 1 |
| Sohn JH, Park YW, Chang SM, Kim BS, Kim SK, Cho MJ. Comorbid patterns of psychiatric disorders in Korean communities. *European Neuropsychopharmacology*. 2008 2008;18:S286. | 1 |
| Song J. How the impact of chronic pain on mental health varies by age and interference with life. *Psychosomatic Medicine*. 2019 2019;81(4):A61. doi:10.1097/PSY.0000000000000699 | 1 |
| Sousa NFDS, Barros MBA, Medina LPB, Malta DC, Szwarcwald CL. Association of major depressive disorder with chronic diseases and multimorbidity in Brazilian adults, stratified by gender: 2019 National Health Survey. *Revista Brasileira de Epidemiologia*. 2021 2021;24. doi:10.1590/1980-549720210015.SUPL.2 | 5 |
| Stanetić K, Petrović V, Stanetić B, et al. Screening of undiagnosed depression among elderly primary care patients: A cross-sectional study from the republic of srpska, Bosnia and Herzegovina. *Medicinski Glasnik*. 2020 2020;17(1):200-205. doi:10.17392/1103-20 | 4 |
| Stegmann ME, Ormel J, De Graaf R, et al. Functional disability as an explanation of the associations between chronic physical conditions and 12-month major depressive episode. *Journal of Affective Disorders*. 2010 2010;124(1):38-44. doi:10.1016/j.jad.2009.10.026 | 4 |
| Sternbach N, Chapnick J, Mould-Quevedo J. Burden of disease of pain in russia: Results from 2011 national health and wellness survey (NHWS). *Value in Health*. 2013 2013;16(3):A127. doi:10.1016/j.jval.2013.03.611 | 1 |
| Stewart DE. Physical symptoms of depression: Unmet needs in special populations. *Journal of Clinical Psychiatry*. 2003 2003;64:12-16. | 1 |
| Stewart ST, Woodward RM, Rosen AB, Cutler DM. The impact of symptoms and impairments on overall health in US national health data. *Med Care*. 2008-9 2008;46(9):954-62. doi:10.1097/MLR.0b013e318179199f | 6 |
| Stickley A, Leinsalu M, DeVylder JE, Inoue Y, Koyanagi A. Sleep problems and depression among 237 023 community-dwelling adults in 46 low- and middle-income countries. *Sci Rep*. 2019-8-19 2019;9(1):12011. doi:10.1038/s41598-019-48334-7 | 6 |
| Stockmarr A, Frølich A. Clusters from chronic conditions in the Danish adult population. *PLoS ONE*. 2024 2024;19(4). doi:10.1371/journal.pone.0302535 | 5 |
| Strine TW, Hootman JM, Chapman DP, Okoro CA, Balluz L. Health-related quality of life, health risk behaviors, and disability among adults with pain-related activity difficulty. *Am J Public Health*. 2005-11 2005;95(11):2042-8. doi:10.2105/ajph.2005.066225 | 5 |
| Strine TW, Kroenke K, Dhingra S, et al. The associations between depression, health-related quality of life, social support, life satisfaction, and disability in community-dwelling US adults. *J Nerv Ment Dis*. 2009-1 2009;197(1):61-4. doi:10.1097/NMD.0b013e3181924ad8 | 5 |
| Stubbs B, Koyanagi A, Schuch FB, et al. Physical activity and depression: A large cross‐sectional, population‐based study across 36 low‐ and middle‐income countries. *Acta Psychiatrica Scandinavica*. 2016 2016;134(6):546-556. doi:10.1111/acps.12654 | 5 |
| Stubbs B, Vancampfort D, Veronese N, et al. Depression and pain: primary data and meta-analysis among 237 952 people across 47 low- and middle-income countries. *Psychol Med*. 2017-12 2017;47(16):2906-2917. doi:10.1017/s0033291717001477 | 5 |
| Subramaniarn M, Vaingankar JA, Abdin E, Chong A. Psychiatric morbidity in pain conditions: Results from the Singapore mental health study. *Pain Research and Management*. 2013 2013;18(4):185-190. doi:10.1155/2013/798205 | 5 |
| Suija K, Kalda R, Maaroos HI. Patients with depressive disorder, their co-morbidity, visiting rate and disability in relation to self-evaluation of physical and mental health: a cross-sectional study in family practice. *BMC Fam Pract*. 2009-6-1 2009;10:38. doi:10.1186/1471-2296-10-38 | 5 |
| Suliman S, Stein DJ, Myer L, Williams DR, Seedat S. Disability and treatment of psychiatric and physical disorders in South Africa. *Journal of Nervous and Mental Disease*. 2010 2010;198(1):8-15. doi:10.1097/NMD.0b013e3181c81708 | 5 |
| Sun J, Zheng YB, Liu L, et al. The Impact of Quarantine on Pain Sensation among the General Population in China during the COVID-19 Pandemic. *Brain Sci*. 2022-1-5 2022;12(1). doi:10.3390/brainsci12010079 | 6 |
| Teh CF, Zaslavsky AM, Reynolds CF, 3rd, Cleary PD. Effect of depression treatment on chronic pain outcomes. *Psychosom Med*. 2010-1 2010;72(1):61-7. doi:10.1097/PSY.0b013e3181c2a7a8 | 2 |
| Thapa S, Shmerling RH, Bean JF, Cai Y, Leveille SG. Chronic multisite pain: evaluation of a new geriatric syndrome. *Aging Clin Exp Res*. 2019-8 2019;31(8):1129-1137. doi:10.1007/s40520-018-1061-3 | 5 |
| Thielke SM, Whitson H, Diehr P, et al. Persistence and remission of musculoskeletal pain in community-dwelling older adults: Results from the Cardiovascular Health Study. *Journal of the American Geriatrics Society*. 2012 2012;60(8):1393-1400. doi:10.1111/j.1532-5415.2012.04082.x | 5 |
| Tietjen G, Khubchandani J. Depression has additive effect on headache associated pain comorbidities. *Headache*. 2014 2014;54(8):1423-1424. doi:10.1111/head.12431/abstract | 1 |
| Tragesser SL, Bruns D, Disorbio JM. Borderline personality disorder features and pain: the mediating role of negative affect in a pain patient sample. *Clin J Pain*. 2010-5 2010;26(4):348-53. doi:10.1097/AJP.0b013e3181cd1710 | 2 |
| Tsang A, Von Korff M, Lee S, et al. Common Chronic Pain Conditions in Developed and Developing Countries: Gender and Age Differences and Comorbidity With Depression-Anxiety Disorders. *Journal of Pain*. 2008 2008;9(10):883-891. doi:10.1016/j.jpain.2008.05.005 | 6 |
| van Hecke O, Hocking LJ, Torrance N, et al. Chronic pain, depression and cardiovascular disease linked through a shared genetic predisposition: Analysis of a family-based cohort and twin study. *PLoS One*. 2017 2017;12(2):e0170653. doi:10.1371/journal.pone.0170653 | 5 |
| van Hecke O, Torrance N, Cochrane L, et al. Does a history of depression actually mediate smoking‐related pain? Findings from a cross‐sectional general population‐based study. *European Journal of Pain*. 2014 2014;18(9):1223-1230. doi:10.1002/j.1532-2149.2014.00470.x | 6 |
| Vanasse A, Courteau M, Courteau J, et al. *Chronic pain and depression: A complex epidemiological picture*. Springer-Verlag Publishing/Springer Nature; 2014:81-97. doi: doi:10.1007/978-2-8178-0414-9_6 | 1 |
| Vancampfort D, Hallgren M, Schuch F, et al. Sedentary behavior and depression among community-dwelling adults aged ≥50 years: Results from the irish longitudinal study on Ageing. *J Affect Disord*. 2020-2-1 2020;262:389-396. doi:10.1016/j.jad.2019.11.066 | 5 |
| Vasiliadis HM, D'Aiuto C, Lamoureux-Lamarche C, Pitrou I, Gontijo Guerra S, Berbiche D. Pain, functional disability and mental disorders as potential mediators of the association between chronic physical conditions and suicidal ideation in community living older adults. *Aging Ment Health*. 2022-4 2022;26(4):791-802. doi:10.1080/13607863.2021.1913478 | 6 |
| Vega R, Groenewald C, Bromberg MH, Beals‐Erickson SE, Palermo TM. Chronic pain prevalence and associated factors in adolescents with and without physical disabilities. *Developmental Medicine & Child Neurology*. 2018 2018;60(6):596-601. doi:10.1111/dmcn.13705 | 2 |
| Vehof J, Williams FMK. Genetic predisposition to chronic pain: From evolutionary advantage to a debilitating disease spectrum? *Pain Management*. 2014 2014;4(6):381-383. doi:10.2217/pmt.14.39 | 1 |
| Vickovic S, Grubor T, Stanisavljevic S, Andjelic N, Zdravkovic R. Cross-sectional study of chronic non-cancer pain, associated depression and quality of life among the population of users of aged care services of the gerontology center. *Acta Anaesthesiologica Scandinavica*. 2023 2023;67(4):537. doi:10.1111/aas.14218 | 1 |
| Von Korff M, Simon G. The relationship between pain and depression. *Br J Psychiatry Suppl*. 1996-6 1996;(30):101-8. doi:10.1192/S0007125000298474 | 1 |
| Vu HTT, Lin V, Pham T, et al. Determining Risk for Depression among Older People Residing in Vietnamese Rural Settings. *Int J Environ Res Public Health*. 2019-7-25 2019;16(15). doi:10.3390/ijerph16152654 | 6 |
| Wang H, Chen K, Zhou B. Living alone, health-related quality of life, life satisfaction, social support, and depressed mood: A population based study among the rural and Urban older adults in China. *American Journal of Epidemiology*. 2011 2011;173:S60. doi:10.1093/aje/kwr181 | 1 |
| Whooley MA. Screening for depression - A tale of two questions. *JAMA Internal Medicine*. 2016 2016;176(4):436-438. doi:10.1001/jamainternmed.2015.8493 | 1 |
| Wiedemann A, Wood AD, Luben RN, et al. Dimension of pain-related quality of life and self-reported mental health in men and women of the European Prospective Investigation into Cancer–Norfolk cohort: a population-based cross-sectional study. *British Journal of Pain*. 2018 2018;12(1):35-46. doi:10.1177/2049463717724566 | 6 |
| Wilkie R, Bucknall M, Jordan K, McBeth J. Widespread and regional pain predict social participation restriction in older adults: A prospective cohort study. *Rheumatology (United Kingdom)*. 2013 2013;52:i37. doi:10.1093/rheumatology/ket200 | 1 |
| Williams LJ, Pasco JA, Jacka FN, Dodd S, Berk M. Pain and the relationship with mood and anxiety disorders and psychological symptoms. *J Psychosom Res*. 2012-6 2012;72(6):452-6. doi:10.1016/j.jpsychores.2012.03.001 | 6 |
| Wittchen HU. The comorbidity of pain, anxiety and depression. *International Journal of Psychiatry in Clinical Practice*. 2013 2013;17:2. doi:10.3109/13651501.2013.851555 | 1 |
| Wong J, Fong D, Chan K. Risk factors associated with somatic complaints in chinese population: Findings from a population-based household survey. *Psychosomatic Medicine*. 2014 2014;76(3):A-89. doi:10.1097/PSY.0000000000000057 | 1 |
| Wong WS, Fielding R. The co-morbidity of chronic pain, insomnia, and fatigue in the general adult population of Hong Kong: Prevalence and associated factors. *J Psychosom Res*. 2012-7 2012;73(1):28-34. doi:10.1016/j.jpsychores.2012.04.011 | 7 |
| Wu CS, Yu SH, Lee CY, Tseng HY, Chiu YF, Hsiung CA. Prevalence of and risk factors for minor and major depression among community-dwelling older adults in Taiwan. *Int Psychogeriatr*. 2017-7 2017;29(7):1113-1121. doi:10.1017/s1041610217000199 | 5 |
| Wu LR, Parkerson Jr GR, Doraiswamy PM. Health perception, pain, and disability as correlates of anxiety and depression symptoms in primary care patients. *The Journal of the American Board of Family Practice / American Board of Family Practice*. 2002 2002;15(3):183-190. | 6 |
| Wynne-Jones G, Chen Y, Croft P, et al. Secular trends in work disability and its relationship to musculoskeletal pain and mental health: a time-trend analysis using five cross-sectional surveys (2002-2010) in the general population. *Occup Environ Med*. 2018-12 2018;75(12):877-883. doi:10.1136/oemed-2018-105171 | 5 |
| Xu A, Hilton E, Arkema R, Tintle NL, Helming LM. Epidemiology of chronic pain in Ukraine: Findings from the World Mental Health Survey. *PLoS One*. 2019 2019;14(10):e0224084. doi:10.1371/journal.pone.0224084 | 6 |
| Yamada K, Kubota Y, Iso H, Oka H, Katsuhira J, Matsudaira K. Association of body mass index with chronic pain prevalence: a large population-based cross-sectional study in Japan. *J Anesth*. 2018-6 2018;32(3):360-367. doi:10.1007/s00540-018-2486-8 | 7 |
| Yan Z, Lu R, Li Y, Zheng Z. Exploring the Risk Factors for Depressive Symptoms Among Chinese Rural Older Adults. *J Psychosoc Nurs Ment Health Serv*. 2020-2-1 2020;58(2):41-47. doi:10.3928/02793695-20191030-01 | 6 |
| Yongjun Z, Tingjie Z, Xiaoqiu Y, et al. A survey of chronic pain in China. *Libyan J Med*. 2020-12 2020;15(1):1730550. doi:10.1080/19932820.2020.1730550 | 4 |
| Zarei S, Bigizadeh S, Pourahmadi M, Ghobadifar MA. Chronic Pain and Its Determinants: A Population-based Study in Southern Iran. *Korean J Pain*. 2012-10 2012;25(4):245-53. doi:10.3344/kjp.2012.25.4.245 | 7 |
| Zarit SH, Griffiths PC, Berg S. Pain perceptions of the oldest old: a longitudinal study. *Gerontologist*. 2004-8 2004;44(4):459-68. doi:10.1093/geront/44.4.459 | 7 |
| Zenger M, Schaefert R, Van Der Feltz-Cornelis C, Brähler E, Häuser W. Validation of the patient-doctor-relationship Questionnaire (PDRQ-9) in a representative cross-sectional German population survey. *PLoS ONE*. 2014 2014;9(3). doi:10.1371/journal.pone.0091964 | 7 |
| Zhao SS, Holmes MV, Alam U. Disentangling the relationship between depression and chronic widespread pain: A Mendelian randomisation study. *Seminars in Arthritis and Rheumatism*. 2023 2023;60. doi:10.1016/j.semarthrit.2023.152188 | 2 |
| ***Forward and backward citation searching*** | |
| Annagür BB, Uguz F, Apiliogullari S, Kara I, Gunduz S. Psychiatric Disorders and Association with Quality of Sleep and Quality of Life in Patients with Chronic Pain: A SCID-Based Study. vol 15. 2014. doi:10.1111/pme.12390 | 2 |
| Arnow BA, Hunkeler EM, Blasey CM, et al. Comorbid depression, chronic pain, and disability in primary care. vol 68. 2006. doi:10.1097/01.psy.0000204851.15499.fc | 5 |
| Astutik E, Hidajah AC, Tama TD, Efendi F, Li CY. Prevalence and determinants of depressive symptoms among adults in Indonesia: A cross-sectional population-based national survey. vol 56. 2021. doi:10.1111/nuf.12508 | 4 |
| Baladón L, Fernández A, Rubio-Valera M, et al. Prevalence of mental disorders in non-demented elderly people in primary care. vol 27. 2015. doi:10.1017/S1041610214002841 | 5 |
| Barragan-Berlanga AJ, Mejia-Arango S, Gutierrez-Robledo LM. [Pain in the elderly: prevalence and associated factors] Dolor en adultos mayores de 50 anos: prevalencia y factores asociados. vol 49. 2007. doi:10.1590/S0036-36342007001000008 | 7 |
| Barrenetxea J, Pan A, Feng QS, Koh WP. Factors associated with depression across age groups of older adults: The Singapore Chinese health study. vol 37. 2022. doi:10.1002/gps.5666 | 5 |
| Beekman ATF, Deeg DJH, Smit JH, Vantilburg W. PREDICTING THE COURSE OF DEPRESSION IN THE OLDER POPULATION - RESULTS FROM A COMMUNITY-BASED STUDY IN THE NETHERLANDS. vol 34. 1995. doi:10.1016/0165-0327(94)00103-G | 7 |
| Beekman ATF, Deeg DJH, vanTilburg T, Smit JH, Hooijer C, vanTilburg W. Major and minor depression in later life: A study of prevalence and risk factors. vol 36. 1995. doi:10.1016/0165-0327(95)00061-5 | 4 |
| Beekman ATF, Kriegsman DMW, Deeg DJH, Vantilburg W. THE ASSOCIATION OF PHYSICAL HEALTH AND DEPRESSIVE SYMPTOMS IN THE OLDER POPULATION - AGE AND SEX-DIFFERENCES. vol 30. 1995. doi:10.1007/BF00784432 | 7 |
| Black SA, Markides KS, Miller TQ. Correlates of depressive symptomatology among older community-dwelling Mexican Americans: The Hispanic EPESE. vol 53. 1998. doi:10.1093/geronb/53B.4.S198 | 4 |
| Blay SL, Andreoli SB, Fillenbaum GG, Gastal FL. Depression morbidity in later life: Prevalence and correlates in a developing country. vol 15. 2007. doi:10.1097/JGP.0b013e3180654179 | 4 |
| Blyth FM, March LM, Brnabic AJM, Cousins MJ. Chronic pain and frequent use of health care. vol 111. 2004. doi:10.1016/j.pain.2004.05.020 | 6 |
| Blyth FM, March LM, Brnabic AJM, Jorm LR, Williamson M, Cousins MJ. Chronic pain in Australia: a prevalence study. vol 89. 2001. doi:10.1016/S0304-3959(00)00355-9 | 6 |
| Bokermann J, König HH, Hajek A. Pain: its prevalence and correlates among the oldest old. vol 36. 2024. doi:10.1007/s40520-023-02653-y | 4 |
| Braden JB, Zhang L, Zimmerman FJ, Sullivan MD. Employment outcomes of persons with a mental disorder and comorbid chronic pain. vol 59. 2008. doi:10.1176/appi.ps.59.8.878 | 6 |
| Brinda EM, Rajkumar AP, Attermann J, Gerdtham UG, Enemark U, Jacob KS. Health, Social, and Economic Variables Associated with Depression Among Older People in Low and Middle Income Countries: World Health Organization Study on Global AGEing and Adult Health. vol 24. 2016. doi:10.1016/j.jagp.2016.07.016 | 4 |
| Chou KL, Chi I. Reciprocal relationship between pain and depression in elderly Chinese primary care patients. vol 20. 2005. doi:10.1002/gps.1383 | 7 |
| Dirupo G, Rossel JB, Fournier N, et al. Correlates of chronic pain onset and recovery in the CoLaus cohort. vol 29. 2025. doi:10.1002/ejp.4712 | 7 |
| Dragioti E, Larsson B, Bernfort L, Levin L, Gerdle B. Prevalence of different pain categories based on pain spreading on the bodies of older adults in Sweden: a descriptive-level and multilevel association with demographics, comorbidities, medications, and certain lifestyle factors (PainS65+). vol 9. 2016. doi:10.2147/JPR.S119845 | 5 |
| Dueñas M, Ojeda B, Salazar A, et al. Use and satisfaction with the Healthcare System of the chronic pain patients in Spain: results from a nationwide study. vol 32. 2016. doi:10.1080/03007995.2016.1211519 | 4 |
| Dueñas M, Salazar A, Ojeda B, et al. A Nationwide Study of Chronic Pain Prevalence in the General Spanish Population: Identifying Clinical Subgroups Through Cluster Analysis. vol 16. 2015. doi:10.1111/pme.12640 | 4 |
| Dworkin SF, Vonkorff M, Leresche L. MULTIPLE PAINS AND PSYCHIATRIC DISTURBANCE - AN EPIDEMIOLOGIC INVESTIGATION. vol 47. 1990. [10.1001/archpsyc.1990.01810150039007](https://doi.org/10.1001/archpsyc.1990.01810150039007) | 5 |
| El-Metwally A, Shaikh Q, Aldiab A, et al. The prevalence of chronic pain and its associated factors among Saudi Al-Kharj population; a cross sectional study. vol 20. 2019. doi:10.1186/s12891-019-2555-7 | 6 |
| Gabilondo A, Vilagut G, Pinto-Meza A, Haro JM, Alonso J. Comorbidity of major depressive episode and chronic physical conditions in Spain, a country with low prevalence of depression. vol 34. 2012. doi:10.1016/j.genhosppsych.2012.05.005 | 2 |
| Gerrits M, van Marwijk HWJ, van Oppen P, van der Horst H, Penninx B. Longitudinal association between pain, and depression and anxiety over four years. vol 78. 2015. doi:10.1016/j.jpsychores.2014.10.011 | 5 |
| Gerrits M, van Oppen P, van Marwijk HWJ, Penninx B, van der Horst HE. Pain and the onset of depressive and anxiety disorders. vol 155. 2014. doi:10.1016/j.pain.2013.09.005 | 5 |
| Giraldo-Rodriguez L, Torres-Castro S, Roa-Rojas PA, Alvarez-Cisneros T. Demographic, socioeconomic and health determinants of depressive symptoms in adults 50 years and older from Mexico: a secondary data longitudinal analysis from the Mexican Health and Aging Study. vol 14. 2024. doi:10.1136/bmjopen-2023-075035 | 4 |
| Gureje O, Simon GE, Von Korff M. A cross-national study of the course of persistent pain in primary care. vol 92. 2001. doi:10.1016/S0304-3959(00)00483-8 | 2 |
| Gutiérrez A, Bravo RL, Tobin CT. Loneliness Exacerbates the Association Between Bodily Pain and Depressive Symptoms Among Middle-Aged and Older Latinx Adults. vol 34. 2022. doi:10.1177/08982643221127327 | 7 |
| Häuser W, Schmutzer G, Henningsen P, Brähler E. Chronic pain, pain disease, and satisfaction of patients with pain treatment in Germany. Results of a representative population survey. vol 28. 2014. doi:10.1007/s00482-014-1438-y | 6 |
| Heidemann C, Scheidt-Nave C, Beyer A-K, et al. Health situation of adults in Germany - Results for selected indicators from GEDA 2019/2020-EHIS. vol 6. 2021. doi:10.25646/8459 | 4 |
| Henderson JV, Harrison CM, Britt HC, Bayram CF, Miller GC. Prevalence, Causes, Severity, Impact, and Management of Chronic Pain in Australian General Practice Patients. vol 14. 2013. doi:10.1111/pme.12195 | 4 |
| Ho CSH, Feng L, Fam J, Mahendran R, Kua EH, Ng TP. Coexisting medical comorbidity and depression: Multiplicative effects on health outcomes in older adults. vol 26. 2014. doi:10.1017/S1041610214000611 | 4 |
| Hotopf M, Mayou R, Wadsworth M, Wessely S. Temporal relationships between physical symptoms and psychiatric disorder - Results from a national birth cohort. vol 173. 1998. doi:10.1192/bjp.173.3.255 | 5 |
| Inoue S, Kobayashi F, Nishihara M, et al. Chronic Pain in the Japanese Community-Prevalence, Characteristics and Impact on Quality of Life. vol 10. 2015. doi:10.1371/journal.pone.0129262 | 6 |
| Ji LL, Qiao XX, Jin YR, Si HX, Liu XY, Wang CL. "Age-Related Positivity Effect" in the Relationship Between Pain and Depression Among Chinese Community-Dwelling Older Adults: Sex Differences. vol 22. 2021. doi:10.1016/j.pmn.2021.02.010 | 7 |
| Kovacs N, Biro E, Piko P, Ungvari Z, Adany R. Age-related shifts in mental health determinants from a deprived area in the European Union: informing the national healthy aging program of Hungary. 2024. doi:10.1007/s11357-024-01182-4. doi:10.1177/0898264314556617 | 4 |
| Kulkarni RS, Shinde RL. Depression and Its Associated Factors in Older Indians: A Study Based on Study of Global Aging and Adult Health (SAGE)-2007. vol 27. 2015. | 4 |
| Langley PC, Ruiz-Iban MA, Molina JT, De Andres J, Castellon JRG-E. The prevalence, correlates and treatment of pain in Spain. vol 14. 2011. doi:10.3111/13696998.2011.583303 | 5 |
| Lee S, Ma YL, Tsang A. COMMUNITY-BASED CO-MORBIDITY OF DEPRESSION AND CHRONIC PHYSICAL ILLNESSES IN HONG KONG. vol 40. 2010. doi:10.2190/PM.40.3.h | 4 |
| Lee S, Tsang A, Huang YQ, et al. The epidemiology of depression in metropolitan China. vol 39. 2009. doi:10.1017/S0033291708004091 | 4 |
| Li YX, Liu MH, Sun XC, Hou TX, Tang SY, Szanton SL. Independent and synergistic effects of pain, insomnia, and depression on falls among older adults: a longitudinal study. vol 20. 2020. doi:10.1186/s12877-020-01887-z | 2 |
| Liberman O, Freud T, Peleg R, Keren A, Press Y. Chronic pain and geriatric syndromes in community-dwelling patients aged ≥65 years. vol 11. 2018. doi:10.2147/JPR.S160847 | 2 |
| Mallon T, Eisele M, König HH, et al. Lifestyle Aspects As A Predictor Of Pain Among Oldest-Old Primary Care Patients - A Longitudinal Cohort Study. vol 14. 2019. doi:10.2147/CIA.S217431 | 7 |
| Mäntyselkä PT, Turunen JHO, Ahonen RS, Kumpusalo EA. Chronic pain and poor self-rated health. vol 290. 2003. doi:10.1001/jama.290.18.2435 | 7 |
| Meher T, Muhammad T, Gharge S. The association between single and multiple chronic conditions and depression among older population in India: A comparative study between men and women. vol 37. 2022. doi:10.1002/gps.5639 | 4 |
| Mojtabai R, Olfson M. Major depression in community-dwelling middle-aged and older adults: prevalence and 2-and 4-year follow-up symptoms. vol 34. 2004. doi:10.1017/S0033291703001764 | 4 |
| Mookerjee N, Schmalbach N, Antinori G, et al. Association of Risk Factors and Comorbidities With Chronic Pain in the Elderly Population. vol 15. 2024. doi:10.1177/21501319241233463 | 5 |
| Murrell SA, Himmelfarb S, Wright K. PREVALENCE OF DEPRESSION AND ITS CORRELATES IN OLDER ADULTS. vol 117. 1983. doi:10.1093/oxfordjournals.aje.a113528 | 4 |
| Nakua EK, Amissah J, Tawiah P, Barnie B, Donkor P, Mock C. The prevalence and correlates of depression among older adults in greater Kumasi of the Ashanti region. vol 23. 2023. doi:10.1186/s12889-023-15361-z | 4 |
| Nicolosi GT, Falcao DVD, Batistoni SST, et al. Depressive symptoms in old age: relations among sociodemographic and self-reported health variables. vol 23. 2011. doi:10.1017/S1041610211000627 | 2 |
| Patel KV, Guralnik JM, Phelan EA, et al. Symptom Burden Among Community-Dwelling Older Adults in the United States. vol 67. 2019. doi:10.1111/jgs.15673 | 7 |
| Peltzer K, Phaswana-Mafuya N. Depression and associated factors in older adults in South Africa. vol 6. 2013. doi:10.3402/gha.v6i0.18871 | 4 |
| Pengpid S, Peltzer K. A Cross-Sectional and Longitudinal Study of Pain among Middle-Aged and Older Adults in Thailand. vol 2023. 2023. doi:10.1155/2023/1158899 | 6 |
| Phiri YVA, Aydin K, Yildiz NG, et al. Individual-level determinants of depressive symptoms and associated diseases history in Turkish persons aged 15 years and older: A population-based study. vol 13. 2022. doi:10.3389/fpsyt.2022.983817 | 4 |
| Reyes-Gibby CC, Aday LA, Todd KH, Cleeland CS, Anderson KO. Pain in aging community-dwelling adults in the United States: Non-Hispanic whites, non-Hispanic blacks, and Hispanics. vol 8. 2007. doi:10.1016/j.jpain.2006.06.002 | 6 |
| Rustoen T, Wahl AK, Hanestad BR, Lerdal A, Paul S, Miaskowski C. Prevalence and characteristics of chronic pain in the general Norwegian population. vol 8. 2004. doi:10.1016/j.ejpain.2004.02.002 | 4 |
| Saito T, Chen T, Yatsugi H, Chu TS, Liu X, Kishimoto H. Independent and combined associations of depressive symptoms and sleep disturbance with chronic pain in community-dwelling older adults. vol 7. 2022. doi:10.1097/PR9.0000000000001034 | 6 |
| Scott KM, Lim C, Al-Hamzawi A, et al. Association of Mental Disorders With Subsequent Chronic Physical Conditions World Mental Health Surveys From 17 Countries. vol 73. 2016. doi:10.1001/jamapsychiatry.2015.2688 | 2 |
| Scudds RJ, Ostbye T. Pain and pain-related interference with function in older Canadians: the Canadian study of health and aging. vol 23. 2001. doi:10.1080/09638280110043942 | 4 |
| Simon GE, VonKorff M, Piccinelli M, Fullerton C, Ormel J. An international study of the relation between somatic symptoms and depression. vol 341. 1999. doi:10.1056/NEJM199910283411801 | 5 |
| Stubbs B, Vancampfort D, Veronese N, et al. Depression and physical health multimorbidity: primary data and country-wide meta-analysis of population data from 190 593 people across 43 low- and middle-income countries. vol 47. 2017. doi:10.1017/S0033291717000551 | 5 |
| Subramaniam M, Abdin E, Sambasivam R, et al. Prevalence of Depression among Older Adults-Results from the Well-being of the Singapore Elderly Study. vol 45. 2016. 10.47102/annals-acadmedsg.V45N4p123 | 4 |
| Thapa DK, Visentin DC, Kornhaber R, Cleary M. Prevalence and factors associated with depression, anxiety, and stress symptoms among older adults: A cross-sectional population-based study. vol 22. 2020. doi:10.1111/nhs.12783 | 4 |
| Torija JRU, Mayor JMF, Salazar MPG, Buisán LT, Fernández RMT. Depressive symptoms in the elderly.: Prevalence and associated factors. vol 21. 2007. doi:10.1157/13099119 | 5 |
| Vietri J, Otsubo T, Montgomery W, Tsuji T, Harada E. The incremental burden of pain in patients with depression: results of a Japanese survey. vol 15. 2015. doi:10.1186/s12888-015-0488-8 | 2 |
| Wark S, Hussain R, Janicki MP, Knox M, Parmenter T. A comparative analysis of the prevalence and predictors of chronic pain in older adults with and without intellectual disability in Australia. vol 49. 2024. doi:10.3109/13668250.2023.2238482 | 2 |
| Werneck AO, Stubbs B. Bidirectional relationship between chronic pain and depressive symptoms in middle-aged and older adults. vol 89. 2024. doi:10.1016/j.genhosppsych.2024.05.007 | 7 |
| Wettstein M, Tesarz J. Increasing pain prevalence and intensity among middle-aged and older adults: Evidence from the German Ageing Survey. vol 168. 2023. doi:10.1016/j.jpsychores.2023.111233 | 7 |
| Wong WS, Fielding R. Prevalence and Characteristics of Chronic Pain in the General Population of Hong Kong. vol 12. 2011. doi:10.1016/j.jpain.2010.07.004 | 6 |
| Xi YT, Mielenz TJ, Andrews HF, et al. Prevalence of Depression in Older Adults and the Potential Protective Role of Volunteering: Findings From the LongROAD Study. 2025. doi:10.1111/jgs.19349 | 2 |
| Yadav UN, Thapa TB, Mistry SK, Pokhrel R, Harris MF. Socio-demographic characteristics, lifestyle factors, multi-morbid conditions and depressive symptoms among Nepalese older adults. vol 20. 2020. doi:10.1186/s12888-020-02680-3 | 4 |
| Zajacova A, Grol-Prokopczyk H, Zimmer Z. Pain Trends Among American Adults, 2002-2018: Patterns, Disparities, and Correlates. vol 58. 2021. doi:10.1215/00703370-8977691 | 5 |
| Note: Reasons for exclusion:  1) No cohort or cross-sectional study  2) Only specific subgroups or settings  3) Sample size <100  4) Assessment of pain and depression not reported  5) Only specific pain sites, pain types or mood disorders assessed and reported  6) Only used combined scales or subscale score not reported  7) No outcome data on pain by depression or depression by pain reported  The assignment of the reasons for exclusion was done hierarchically in the order presented. |  |

Table s3. Assessments of depression for each individual study

| **Assessments of depression** | | | | | | |
| --- | --- | --- | --- | --- | --- | --- |
| **Authors (year)** | **Country** | **Measurement** | **Respondent** | **Format** | **Duration of symptoms** | **Cut-off** |
| Atlantis et al. (2011)(6) | Australia | PAS | Self | Number of items N/R, scale from 0-12, maximum score 12 | Assessing symptoms in the last 2 weeks | Symptomatic depression >5 |
| Wong et al. (2020)(7) | Australia | KICA-dep | Self | N/R | N/R | Clinically significant depression ≥8 |
| Costa Cabral et al. (2014)(8) | Brazil | HADS | Self | 14 items (7 items each for two subscales: HADS-A and HADS-D), 4-point scale, overall/subscale score N/R | N/R | Present depression and/or anxiety >8 |
| Shega et al. (2013)(9) | Canada | MHI-5 | Self | 5 items, 6-point scale, maximum score 30 | N/R | Depression ≥12 |
| Tripp et al. (2006)(10) | Canada | PHQ-9 | Self | 9 items, 4-point scale, score N/R | N/R | N/R |
| Del pilar et al. (2024)(11) | Chile | GDS-15 | Self | 15 items, scale N/R, maximum score 15 | N/R | Depression >5, severe depression >10 |
| Moreno et al. (2022)(12) | Chile | GDS-15, one close-ended question | Self | 15 items, dichotomy (yes/no), scale N/R, maximum score 15; One close-ended question on diagnosis of depression by a doctor, scale or score N/A | Assessing symptoms in the last week | Depression ≥5 |
| Chi et al. (2004)(13) | China | GDS-15 | Self | 15 items, dichotomy (yes/no), scales or scores N/R, maximum score 15 | Assessing symptoms in the last 2 weeks | Depression ≥8 |
| Chen et al. (2012)(14) | China | CIDI-3.0 | Self | 9 items (2 core items plus 7 other items);  Duration/frequency, scales or scores N/R | Symptoms most of the day/nearly every day for 2 weeks per year | MDD: ≥1 core symptom plus 4 other symptoms, depressive symptoms without MDD: ≥1 core symptom plus <4 other symptoms |
| Gong et al. (2017)(15) | China | GDS-15 | Self | 15 items, dichotomy (yes/no), scale N/R, maximum score 15 | Assessing symptoms in the last week | Depression ≥6 |
| Liu et al. (2018)(16) | China | PHQ-9 | Self | 9 items, 4-point scale, maximum score 27 | N/R | Non-minimal: 0-4, slight: 5-9, moderate: 10-14,  moderately severe: 15-19, severe: 20-27 |
| Ma et al. (2021)(17) | China | CES-D | Self | 10 items, scales or scores N/R | N/R | Depression ≥10 |
| Si et al. (2019)(18) | China | GDS-5 | Self | 5 items, scales or scores N/R | N/R | Depression ≥2 |
| Tian et al. (2017)(19) | China | GDS-5 | Self | 5 items, dichotomy (yes/no), scale N/R, maximum score 5 | Assessing symptoms during the last week | Depression ≥2 |
| Borda et al. (2016)(20) | Colombia | GDS-15 | Self | 15 items, scale N/R, maximum score 15 | Assessing symptoms during the last week | Moderate depression: 6-10, severe depression: 11-15 |
| Arola et al. (2010)(21) | England | HADS | Self | 14 items (7 items each for two subscales: HADS-A and HADS-D), 4-point Likert scale, maximum subscale score 21 | N/R | Possible anxiety/depression: 8-10,  probable anxiety/depression >11 |
| Chou (2006)(22) | England | CES-D | Self | 8 items, dichotomy (yes/no), scales or scores N/R | N/R | Depression ≥3 |
| Westoby et al. (2008)(23) | England | HADS | Self | 14 items (7 items each for two subscales: HADS-A and HADS-D), 4-point scale, maximum subscale score 21 | N/R | Possible anxiety/depression: 8-21 |
| Ahrend et al. (2024)(24) | Germany | PHQ-8 | Self | 8 items, scale N/R, maximum score 24 | Assessing symptoms during the last 2 weeks | Depression ≥10 |
| Bauer et al. (2016)(25) | Germany | GDS-15 | Self | 15 items, dichotomy (yes/no), scale N/R, maximum score 15 | Assessing symptoms during the last 2 weeks | Depressed mood ≥5 |
| Denkinger et al. (2014)(26) | Germany | HADS | Self | 7 items (HADS-D), scales or scores N/R | N/R | Depression ≥8 |
| Häuser et al. (2013)(27) | Germany | PHQ-4 | Self | 4 items (2 items each from PHQ-2 and GAD-2), 4-point scale, overall/subscale score N/R | N/R | Depression ≥3 |
| Pengpid & Peltzer (2021)(28) | India | CIDI-SF | Self | 9 items, dichotomy (yes/no), scale N/R, 7 additional symptoms, maximum score 7 | 2 symptoms for most of the day ≥2 weeks | Depression ≥3 |
| Regan et al. (2013)(29) | Ireland | CES-D | Self | 20 items, scale N/R, maximum score 60 | N/R | Depression ≥16 |
| Goral et al. (2010)(30) | Israel | One close-ended question | Self | Diagnosis of depressive disorders (major depression, dysthymic disorder, minor depressive episode), diagnosis of anxiety disorders (generalized anxiety disorder, panic disorder, agoraphobia), scales or scores N/A | During the last 12 months | N/R |
| Yunus et al. (2018)(31) | Malaysia | GDS-15 | Self | 15 items, scales or scores N/R | N/R | N/R |
| Garcia-Perez et al. (2022)(32) | Mexico | CES-D | Self | 9 items, dichotomy (yes/no), scale N/R, maximum score 9 | Assessing symptoms during the last week | Low depressive symptoms ≤4,  high depressive symptoms ≥5 |
| Gutierrez et al. (2022)(33) | Mexico | CES-D | Self | 9 items, dichotomy (yes/no), scale N/R, maximum score 9 | Assessing symptoms during the last week | Depression ≥5 |
| Geerlings et al (2001)(34) | Netherlands | CES-D | Self | 20 items, 4-point scale from “rarely/none of the time” to “most/all of the time”, maximum score 60 | Assessing symptoms during the last week | Depression ≥16 |
| Hilderink et al. (2012)(35) | Netherlands | CES-D | Self | 20 items, 4-point scale from “rarely/none of the time” to “most/all of the time”, maximum score 60 | Assessing symptoms during the last week | Depression ≥16 |
| Gureje et al. (2009)(36) | Nigeria | CIDI 3.0 | Self | Presence of multiple disorders including depression, dichotomy (yes/no), scales or scores N/R | In the last 12 months | N/R |
| Satghare et al. (2016)(37) | Singapore | GMS-AGECAT | Self/by proxy | Semi-structured questionnaire for 9 diagnoses including depression, 6-point scale from “0=non-case" to “5=severe case”, score N/R | N/R | N/R |
| Zager et al. (2023)(38) | Slovenia | PHQ-8 | Self | 8 items, 4-point scale, score N/R | Assessing symptoms during the last 2 weeks | N/R |
| Dueñas et al. (2024)(39) | Spain | HADS | Self | 14 items (7 items each for two subscales), 4-point scale, maximum subscale score 21, overall score N/R | N/R | Possible anxiety/depression: 8-10,  presence anxiety/depression >10 |
| Rivera et al. (2019) (40) | Spain | MINI | Self | 2 items per disorder (16 mental disorders in total), dichotomy (yes/no), scales or scores N/R | N/R | N/R |
| Sendra-Gutierrez et al. (2017)(41) | Spain | PHQ-8 | Self | Number of items N/R, scales or scores N/R | Assessing symptoms during the last 2 weeks | N/R |
| Thomtén et al. (2011)(42) | Sweden | GHQ-12 | Self | 12 items, scale N/R, maximum score 12 | N/R | Depression ≥3 |
| Rouch et al. (2023)(43) | Switzerland | DIGS | Self | N/R | N/R | N/R |
| Ko et al. (2018)(44) | Taiwan | CES-D | Self | 20 items, 4-point scale, maximum score 60 | Assessing symptoms during the last week | Depression ≥16 |
| Erbaydar & Çilingiroğlu (2010)(45) | Turkey | BDI | Self | N/R | N/R | Depression ≥17 |
| Bollinger et al. (2020)(46) | USA | Three close-ended questions | Self | Assessing frequency and intensity of symptoms; medication intake, scale N/R, score N/A | N/R | Depressive symptoms: frequency of symptoms “a few times a year”, medication intake and symptom intensity at least “a little” |
| Emptage et al. (2005)(47) | USA | CES-D | Self | 8 items, dichotomy (yes/no), scale N/R, maximum score 8 | Assessing symptoms during the last week | Depression ≥3 |
| Janevic et al. (2017)(48) | USA | CES-D | Self | 8 items, dichotomy (yes/no), scales or scores N/R | N/R | Depression ≥4 |
| McCarthy et al. (2009)(49) | USA | One close-ended question | Self | Diagnosis of depression, dichotomy (yes/no), scales or scores N/A | N/R | N/R |
| Miller & Cano (2009)(50) | USA | SCID | Self | 9 items, dichotomy (presence/absence), scales or scores N/R | Assessing symptoms during the last 2 weeks | Probable depression ≥5; at least one “feeling down, depressed or hopeless” or “little interest or pleasure doing things” |
| Mullins et al. (2023)(51) | USA | PHQ-8 | Self | 8 items, 4-point scale from “none/minimal” to “severe”, assessing symptom intensity, score N/R | N/R | N/R |
| Ohayon & Schatzberg (2010)(52) | USA | Sleep-EVAL | Self | 48 items (positive answers trigger further items), 12 symptoms on 4-point scales assessing intensity and frequency, 4-point scale for weight change, maximum score 56 | N/R | N/R |
| Patel et al. (2013)(53) | USA | PHQ-2 | Self | 2 items, scales or scores N/R | N/R | Depression ≥3 |
| Shega et al. (2014)(54) | USA | CES-D | Self | 11 items, scale N/R, score from 0-22 | N/R | N/R |
| Shi et al. (2010)(55) | USA | CES-D | Self | 8 items, scale N/R, score from 0-8 | N/R | Depression ≥4 |
| Steffens et al. (2009)(56) | USA | CIDI-SF, NPI | Self/by proxy | CIDI-SF: 8 items, dichotomy (yes/no), scales or scores N/R  NPI: 12 neuropsychiatric disorders including depression; intensity and frequency in the last 2 weeks, scales or scores N/R | Assessing symptoms in the last 12 months | Depression: CIDI-SF ≥5; NPI N/R |
| Tian et al. (2005)(57) | USA | CES-D, CIDI-SF | Self | CES-D: 8-items, scale N/R, maximum score 8;  CIDI-SF: Number of items N/R, scale from 0-7, score N/R | Symptoms during the last week “all” or “most of the time” | Depression: CES-D: ≥4; CIDI-SF: ≥3 |
| Uebelacker et al. (2014)(58) | USA | CES-D | Self | 6-items, scale N/R, maximum score 18 | Assessing symptoms during the last week | Depression: ≥5 |
| Qiu et al (2022)(59) | China & England | CES-D | Self | CHARLS: 10 items, dichotomy (yes/no), scale N/R, maximum score 30;  ELSA: 8 items, dichotomy (yes/no), scale N/R, maximum score 8 | N/R | CHARLS: depression ≥12;  ELSA: depression ≥4 |
| Langley (2011)(60) | Europe | One close-ended question | Self | scales or scores N/A | Assessing depression in the last 12 months | N/R |
| Ohayon & Schatzberg (2003)(61) with companion report Ohayon (2004)(62) | Europe | Sleep-EVAL | Self | Ohayon und Schatzberg (2003): Number of items N/R, intensity on 6-point scale from “does not know” to “extremely”, frequency scale “most of the day” to “nearly every day”, treatment; further scales or scores N/R;  Ohayon (2004): 36 items (positive answers trigger further items), dichotomy (presence/absence) intensity on 6-point scale from “does not know” to “extremely”, further scales or scores N/R | N/R | N/R |
| Horackova et al. (2018)(63) | Europe and Israel | EURO-D | Self | 12 items, dichotomy (not present/present) scale N/R, maximum score 12 | Assessing symptoms during the last month | Major depression ≥4 |
| Ogliari et al. (2023)(64) | Europe and Israel | EURO-D | Self | 12 items, dichotomy (not present/present) scale N/R, maximum score 12 | Assessing symptoms during the last month | Major depression ≥4 |
| Raggi et al. (2020)(65) | China, Europe & Mexico | One close-ended question | Self | Assessing presence of depression, scales or scores N/A | N/R | N/R |
| Gureje et al. (2007)(66) | Worldwide | CIDI-WMH | Self | N/R | N/R | N/R |
| **Abbreviations:** BDI: Becks Depression Inventory; CIDI: Composite International Diagnostic Interview; CES-D: Center for Epidemiologic Studies Depression Scale; DIGS: Diagnostic Interview for Genetic Studies; GHQ-12: General Health Questionnaire 12; GMS-AGECAT: Geriatric Mental State Automated Geriatric Examination for Computer Assisted Taxonomy; HADS: Hospital Anxiety and Depression Scale; PHQ: Patient health questionnaire; MHI-5: Mental Health Inventory-5; MINI: Mini International Neuropsychiatric Interview; NPI: Neuropsychiatric Interview; N/R: Not reported; PAS: Psychogeriatric Assessment Scales; SCID: Structured Clinical Interview for DSM-IV-TR, USA: United States of America | | | | | | |

Table s4 Assessments of pain for each individual study

| **Assessments of pain** | | | | | | | | |
| --- | --- | --- | --- | --- | --- | --- | --- | --- |
| **Authors (year)** | **Country** | **Measurement** | **Respondent** | **Format** | **Characteristics** | **Type of pain (acute/chronic)** | **Cut-off** | |
| Atlantis et al. (2011)(6) | Australia | One close-ended question | Self | Persistent, bothersome or activity limiting pain in the last 12 months, scale from “none” to “daily” | Duration/frequency, presence | N/R | N/R | |
| Wong et al. (2020)(7) | Australia | BPI | Self | 3 items, duration/frequency on 3-point scale from “no pain” to “all of the time”;  intensity on scale from 0-5 “mild” to “severe”, location on “target sign diagram”; overall score N/R | Duration/frequency, intensity, location, presence | N/R | N/R | |
| Costa Cabral et al. (2014)(8) | Brazil | CPG | Self | 8 items, pain intensity and pain-related disability and number of days (disability days) on scale from 0-10, assessing the most bothersome pain in the last 3 months, pain graded into 4 hierarchical classes (“low intensity” to “severely limiting”); overall score N/R | Duration/frequency, intensity, interference, presence | Chronic pain | Chronic pain: pain of any intensity for ≥3 months and at least one episode of pain during the last 30 days | |
| Shega et al. (2013)(9) | Canada | VDS | Self | 1 item, 5-point scale from “none” to “very severe”, assessing symptoms in the last 4 weeks, score N/R | Intensity, presence | N/R | N/R | |
| Tripp et al. (2006)(10) | Canada | GCPS | Self | 7 items, three 11-point scales of pain intensity from “0=no pain” to “10=pain as bad as could be”; interference from “0=no interference” to “10=unable to carry on activities”; duration/frequency number of days with pain in the last 6 months;  Grading:  “I=low interference- low intensity”; “II=low interference-high intensity”; “III =high interference-moderately limiting”; “IV=high interference-severely limiting” | Duration/frequency, intensity, interference, presence | Chronic pain | N/R | |
| Del pilar et al. (2024)(11) | Chile | One close-ended question | Self | Pain in the last 4 weeks on 6-point Likert scale from “no pain” to “severe” | Intensity, presence | N/R | N/R | |
| Moreno et al. (2022)(12) | Chile | One close-ended question | Self | Pain in the last 4 weeks on 6-point scale from “no pain” to “severe” | Intensity, presence | N/R | N/R | |
| Chi et al. (2004)(13) | China | One close-ended question | Self | Pain >6 months, dichotomy (yes/no) | Presence | Chronic pain | N/R | |
| Chen et al. (2012)(14) | China | Four close-ended questions | Self | 4 chronic pain conditions in the last 12 months, dichotomy (yes/no) | Presence | Chronic pain | N/R | |
| Gong et al. (2017)(15) | China | One close-ended question | Self | Pain intensity on 4-point scale from “no” to “severe” | Intensity, presence | N/R | N/R | |
| Liu et al. (2018)(16) | China | One close-ended question, VAS | Self | Pain presence during the last 4 weeks, dichotomy (yes/no); intensity with VAS on 11-point scale from “0=no pain” to “10=worst imaginable pain”; overall score N/R | Intensity, presence | Acute pain | N/R | |
| Ma et al. (2021)(17) | China | Three close ended questions | Self | 5-point scale, location by number of painful sites equals intensity; treatment, dichotomy (yes/no) | Intensity,  location, treatment, presence | N/R | no pain=0, mild pain=1-3, moderate to severe pain=≥4 | |
| Si et al. (2019)(18) | China | Two close-ended questions, FPS-R | Self | Presence of pain, dichotomy (yes/no), duration/frequency in months; intensity with FPS-R visual scale from 0-10, “no pain” to “very much pain”; overall score N/R | Duration/frequency, intensity, presence | Chronic pain | Chronic pain: duration ≥3 months | |
| Tian et al. (2017)(19) | China | FPS-R | Self | Visual scale from 0-10 (in two step mode), assessing current pain, score N/R | Intensity, presence | N/R | Pain ≥2 | |
| Borda et al. (2016)(20) | Colombia | One close-ended question | Self | Pain intensity on 3-point scale from “no pain” to “severe pain” | Intensity, presence | N/R | N/R | |
| Arola et al. (2010)(21) | England | MOS SF-12 | Self | 2 items; 1 item presence in the last 4 weeks for >one day, dichotomy (yes/no); 1 item interference on 5-point scale from “not at all” to “extremely”; overall score N/R | Interference, presence | N/R | N/R | |
| Chou (2006)(22) | England | Two close-ended questions | Self | Presence as “often troubled with pain” (yes/no); intensity 3-point scale from “mild” to “severe” | Intensity, presence | N/R | N/R | |
| Westoby et al (2008)(23) | England | Two close-ended questions | Self | Presence as pain in the last 4 weeks for >one day, dichotomy (yes/no); location on body manikin | Location, presence | N/R | N/R | |
| Ahrend et al. (2024) (24) | Germany | One close-ended question | Self | Pain intensity during the last 4 weeks from “no pain” to “severe” | Intensity, presence | N/R | N/R | |
| Bauer et al. (2016)(25) | Germany | Two close-ended questions | Self | Pain presence per week “once or several times” dichotomy (yes/no); location on manikin | Location, presence | Chronic pain | Chronic local pain (CLP): pain <3 months and not fulfilling ACR-criteria; chronic widespread pain: pain >3 months plus fulfilling ACR-criteria | |
| Denkinger et al. (2014)(26) | Germany | Four close-ended questions, VAS/NRS | Self | Duration/frequency on 4-point scale from “days” to “years” and 6-point scale from “never” to “always” in the last week; intensity of the most painful site in the last week: NRS or VAS scale from 0 to 10; location: 13 body areas plus missing; quality: attacks/breakthrough, continuous, both; overall score N/R | Duration/frequency, intensity, quality, presence | N/R | N/R | |
| Häuser et al. (2013)(27) | Germany | WPI, one closed-ended question | Self | WPI: 1 item each for 19 pain sites in the last 7 days, scales or scores N/R;  One close-ended question: pain presence in the last 3 months, dichotomy (yes or no); overall score N/R | Location, presence | Chronic pain | ≥1 pain location in the last 3 months | |
| Pengpid & Peltzer (2021)(28) | India | One close-ended question | Self | Presence as “troubled by pain” plus treatment | Presence, treatment | N/R | N/R | |
| Regan et al. (2013)(29) | Ireland | Two close-ended questions | Self | Presence as “often suffered from pain”, dichotomy (yes/no); intensity on 3-point scale from “mild” to “severe” | Intensity, presence | Chronic pain | “moderate” or “severe” pain | |
| Goral et al. (2010)(30) | Israel | Three close-ended questions | Self | Presence, diagnosis and/or treatment  in the last 12 months, dichotomy (yes/no);  type: back/neck pain, strong/frequent headache, other | Presence, treatment, type | Chronic pain | Chronic pain: treatment for back/neck pain, strong/frequent headache or other chronic pain during the last year | |
| Yunus et al. (2018)(31) | Malaysia | Two close-ended questions | Self | Presence of chronic pain as ≥3 months in <6 months on every day/almost every day, dichotomy (yes/no); interference with daily work or activities, 5-point Likert scale from “not at all” to “extremely disturbing” | Interference, Presence | Chronic pain | Chronic pain: ≥3 months in <6 months, every day/almost every day persistently, in any part of the body | |
| Garcia-Perez et al. (2022)(32) | Mexico | Three close-ended questions | Self | Presence as “often suffer from pain”, dichotomy (yes/no); intensity on 4-point Likert scale from “without pain” to “severe”; interference with activities, dichotomy (yes/no) | Intensity, interference, presence | N/R | N/R | |
| Gutierrez et al. (2022)(33) | Mexico | Two close-ended questions | Self | Presence as “often suffer from pain”; dichotomy (yes/no); interference with daily activities; dichotomy (yes/no) | Interference, presence | N/R | N/R | |
| Geerlings et al. (2001)(34) | Netherlands | NHP5 | Self | 5 items, dichotomy (yes/no), 6-point scale from “0=low” to “5=high”, maximum score 5 | Presence | N/R | N/R | |
| Hilderink et al. (2012)(35) | Netherlands | NHP5 | Self | 5 items, dichotomy (yes/no), 6-point scale from “0=low” to “5=high”, maximum score 5 | Presence | N/R | Pain ≥2 | |
| Gureje et al. (2009)(36) | Nigeria | One close-ended question | Self | Presence and treatment in the last 12 months; dichotomy (yes/no) | Treatment, presence | Chronic pain | N/R | |
| Satghare et al. (2016)(37) | Singapore | Pain Questionnaire 10/66 | Self/by proxy | 3 items, duration/frequency as pain in the last month on 5-point scale from “never” to “every day”, intensity on 6-point scale from “mild pain” to “unbearable pain”, interference with activities, 4-point scale from “not at all” to “very much”; overall score N/R | Duration/frequency, intensity, interference, presence | N/R | N/R | |
| Zager et al. (2023)(38) | Slovenia | One close-ended question | Self | Pain intensity in the last 4 weeks on 6-point scale from “none” to “very severe” | Intensity, presence | N/R | N/R | |
| Dueñas et al. (2024)(39) | Spain | Two close-ended questions | Self | Duration/frequency as >4-5 days a week during the last month, dichotomy (yes/no); chronicity >3 months; dichotomy (yes/no) | Duration/frequency, presence | Chronic pain | Number of affirmative answers:  Non-chronic continuous pain: yes/no; Chronic pain: yes/yes | |
| Rivera et al. (2019)(40) | Spain | One close-ended question | Self | Type as arthritis/migraine/other; further details N/R | Type, presence | Chronic pain | N/R | |
| Sendra-Gutierrez et al. (2017)(41) | Spain | One close-ended question | Self | Intensity in the last 4 weeks on 6-point scale from “none” to “extreme” | Intensity, presence | N/R | N/R | |
| Thomtén et al. (2012)(42) | Sweden | PQ, VAS | Self | 4 items, pain presence as pain ≥1 month < 3 months, duration/frequency on 6-point scale from “almost every week” to “all the time”; intensity: VAS, scale from 0-10; interference 15 questions, dichotomy (yes/no); location details N/R; overall score N/R | Duration/frequency, intensity, interference, location, presence | N/R | Pain: ≥1 month <3 months | |
| Rouch et al. (2023)(43) | Switzerland | STOPNEP, BPI | Self | STOPNEP: filter questions for presence of chronic pain with positive answers leading to further characteristics; location: open question; duration/frequency: 4-point scale from “<6 months” to “≥3 years”;  BPI: intensity, during the last 24 hours with 3 items (highest, lowest and average intensity), scale from “0=no pain” to “10=worst pain imaginable”, maximum score 10;  overall score N/R | Duration/frequency, intensity, location, presence | Chronic pain | Chronic pain: persistent or recurrent daily pain >3 months | |
| Ko et al. (2018)(44) | Taiwan | Three close-ended questions | Self | Pain presence as any pain in the last year, dichotomy (yes/no); intensity on 3-point scale from “0=no pain” to “3=severe” for each site; location as 10 sites plus elsewhere; maximum score: highest score among all pain sites | Intensity, location, presence | N/R | Any pain in any part of the body for at least one month | |
| Erbaydar & Çilingiroğlu (2010)(45) | Turkey | VAS | Self | 11-point visual scale from “0=no pain” to “10=very severe pain”, score N/R | Intensity, presence | Chronic pain | Mild pain=1-3, moderate to severe pain=4-8, severe pain=9-10 | |
| Bollinger et al. (2020)(46) | USA | Two close-ended questions | Self | Duration/frequency as “never” to “every day” in the last 3 months; intensity from “a little/a lot/somewhere in between”; location and type from list of 14 sites/conditions | Duration/frequency, intensity, location, type, presence | Chronic pain | Pain on most days/every day or some days plus pain level somewhere in between/a lot in the last 3 months | |
| Emptage et al. (2005)(47) | USA | Two close-ended questions | Self | Presence as “often troubled with pain”, dichotomy (yes/no); intensity on 3-point scale from “mild” to “severe” | Intensity, presence | N/R | N/R | |
| Janevic et al. (2017)(48) | USA | Pain Disability Index | Self | Interference on scale from “0=no disability” to “10=total disability” in 7 domains, financial difficulties, dichotomy (yes/no), maximum score N/R; intensity: scale from 0-10, most severe pain on average last year, overall score N/R | Intensity, interference, presence | Chronic pain | High-impact chronic pain: duration of 7 months plus a mean pain impact score =7 in one or more of following areas (family and home, recreation/leisure, social activities, work and basic daily activities) | |
| McCarthy et al. (2009)(49) | USA | TPI | Self | Pain in 8 body areas during the last 3 months on 5-point scale from “none” to “all of the time”; intensity on a scale from 0-10, overall score N/R | Duration/frequency, intensity, location, presence | Chronic pain | Chronic pain: 1 of 2 criteria:  1. Pain in the last three months some/most/all of the time; 2. Pain of moderate or severe intensity (≥4/10) | |
| Miller & Cano (2009)(50) | USA | Three close-ended questions | Self | Presence as pain daily/most of the day over 6 months, dichotomy (yes/no); duration/frequency as “how long have you had this chronic pain?”; diagnosis from a doctor, dichotomy (yes/no); location and types of chronic pain | Duration/frequency, location, type, presence | Chronic pain | Chronic pain daily for most of the day over 6 months | |
| Mullins et al. (2023)(51) | USA | One close-ended question | Self | Duration/frequency as pain in the last 3 months on 4-point scale from “never” to “every day” | Duration/frequency, presence | Chronic pain | Chronic pain “most days” or “every day” in the last 3 months | |
| Ohayon & Schatzberg (2010)(52) | USA | Multiple questions | Self | 11 domains; duration/frequency as days/week in the last 3 months; location from 10 sites; intensity for each site on 7-point scale from “0=no pain” to “6=unbearable pain”, maximum score 54 | Duration/frequency, intensity, interference, location, treatment, type, presence | Chronic pain | Chronic pain: abdomen, shoulders, neck, back, arms, hands, legs, chest, headaches or migraine at interview for 3 days a week in at least mild intensity for at least 3 months | |
| Patel et al. (2013)(53) | USA | Two close-ended questions | Self/by proxy | Presence as “bothered by pain in the last month”, dichotomy (yes/no); location on 12 anatomical sites plus “other” | Location, presence | N/R | N/R | |
| Shega et al (2014)(54) | USA | Two close-ended questions, VDS | Self | Presence as “any pain” in the last 4 weeks; location on a map from 12 body areas; intensity with VDS on 7-point scale from “no pain” to “the most intense pain imaginable”; overall score N/R | Intensity, location, presence | N/R | Persistent pain: duration of 4 weeks | |
| Shi et al. (2010)(55) | USA | Three close-ended questions | Self | Presence as “often troubled with pain”, dichotomy (yes/no); intensity on 3-point scale from “mild” so “severe”; interference with routine/daily life, dichotomy (yes/no) | Intensity, interference, presence | N/R | N/R | |
| Steffens et al. (2009)(56) | USA | Two close-ended questions | Self | Presence as “pain most of the time” with intensity as “moderate” or “severe”; interference with usual activities | Intensity interference, presence | N/R | Pain for most of the time either moderate or severe, or pain interference with usual activities | |
| Tian et al. (2005)(57) | USA | Two close-ended questions | Self | Presence as “often experience pain without reference to physical pathology”, dichotomy (yes/no); intensity, 3-point scale from “mild” to “severe” | Intensity, presence | N/R | N/R | |
| Uebelacker et al. (2014)(58) | USA | SF-36 | Self | 2 items, scales or scores N/R | Presence | Acute pain | N/R | |
| Qiu et al. (2022)(59) | China & England | Two close-ended questions | Self | CHARLS: intensity on 3-point scale from “no pain” to “severe”, 15 locations;  ELSA: intensity on 11-point scale from “0=no pain” to “10=severe or excruciating”, 4 locations | Intensity, location, presence | N/R | N/R  CHARLS: N/R;  ELSA: no pain (scores 0), mild to moderate pain (scored 1-7), severe pain (scored 8-10) | |
| Langley (2011)(60) | Europe | Multiple questions | Self | 9 domains; duration/frequency in the last month on 4-point scale from “daily” to “weekly or less”; intensity on 3-point scale from “mild” to “severe”; type from 5 common causes; treatment | Duration/frequency, intensity, treatment, type, presence | N/R | N/R | |
| Ohayon & Schatzberg (2003)(61) with companion report Ohayon (2004)(62) | Europe | Multiple questions | Self | Ohayon & Schatzberg (2003) and Ohayon (2004):  Duration/frequency at least 6 months, interference with functioning, location and type from 5 conditions, treatment | Interference, location, treatment, type, presence | Chronic pain | Ohayon & Schatzberg (2003): pain > 6 months and consultation of a health care professional;  Ohayon (2004): pain >6 months, impaired daily functioning or medication use | |
| Horackova et al. (2018) (63) | Europe and Israel | One close-ended question | Self | Presence as “troubled by pain” | Presence | N/R | N/R | |
| Ogliari et al. (2023)(64) | Europe and Israel | Two close-ended questions | Self | Pain as “troubled by pain”, dichotomy (yes/no); intensity on 3-point scale from “mild” to “severe” | Intensity, presence | N/R | N/R | |
| Raggi et al. (2020)(65) | China, Europe & Mexico | One close-ended question | Self | Pain presence, dichotomy (present/absent) | Presence | N/R | N/R | |
| Gureje et al. (2007)(66) | Worldwide | U.S. Health Interview Survey | Self | Presence as “pain in the last 12 months/lifetime experience”; location and type from 4 conditions; maximum score of 4; 15 other medical conditions, scale or score N/R | Location, presence, type | Chronic pain | N/R | |
| Abbreviations: CIDI: Composite International Diagnostic Interview; CIDI-WMH: World Mental Health Version of the Composite International Diagnostic Interview; HADS: Hospital Anxiety and Depression Scale; PHQ-4: Patient health questionnaire 2; SCID: Structured Clinical Interview for DSM-IV-TR; CPG: Chronic pain grade; DIGS: Diagnostic Interview for Genetic Studies; GCPS: Graded Chronic Pain Status; GHQ-12: General Health Questionnaire; GMS-AGECAT: Geriatric Mental State Automated Geriatric Examination for Computer Assisted Taxonomy; GPQ: German pain questionnaire; MHI-5: Mental Health Inventory-5; MINI: Mini International Neuropsychiatric Interview; MOS SF-12: Medical outcome survey Short form 12; NHP5: Nottingham Health Profile 5; NPI: Neuropsychiatric Interview; TPI: Total Pain Index; VAS: Visual Analog Scale; VDS: Verbal Descriptor Scale; WPI: Widespread pain index; N/A: not applicable; N/R: not reported; PAS: Psychogeriatric Assessment Scales; PQ: The Pain Questionnaire; VAS: Visual Analog Scale; | | | | | | | |  |

Table s5. Measurements for depression and pain in studies with the primary objective to assess both conditions (n=22)

| **Authors (year)** | **Assessment of depression** | **Assessment of pain** |
| --- | --- | --- |
| Del pilar et al. (2024)(11) | GDS-15 | One close-ended question |
| Chen et al. (2012)(14) | CIDI-3.0 | Four close-ended questions |
| Tian et al. (2017)(19) | GDS-5 | FPS-R |
| Arola et al. (2010)(21) | HADS | MOS SF-12 |
| Chou (2006)(22) | CES-D | Two close-ended questions |
| Ahrend et al. (2024)(24) | PHQ-8 | One close-ended question |
| Denkinger et al. (2014)(26) | HADS | Four close-ended questions, VAS/NRS |
| Gutierrez et al. (2022)(33) | CES-D | Two close-ended questions |
| Geerlings et al. (2001)(34) | CES-D | NHP5 |
| Hilderink et al. (2012)(35) | CES-D | NHP5 |
| Rouch et al. (2023)(43) | DIGS | STOPNEP, BPI |
| Ko et al. (2018)(44) | CES-D | Three close-ended questions |
| Erbaydar & Çilingiroğlu (2010)(45) | BDI | VAS |
| Emptage et al. (2005)(47) | CES-D | Two close-ended questions |
| Miller & Cano (2009)(50) | SCID | Three close-ended questions |
| Mullins et al. (2023)(51) | PHQ-8 | One close-ended question |
| Ohayon & Schatzberg (2010)(52) | Sleep-EVAL | Multiple questions |
| Tian et al. (2005)(57) | CES-D, CIDI-SF | Two close-ended questions |
| Qiu et al. (2022)(59) | CES-D | Two close-ended questions |
| Ohayon & Schatzberg (2003)(61) with companion report Ohayon (2004)(62) | Sleep-EVAL | Multiple questions |
| Ogliari et al. (2023)(64) | EURO-D | Two close-ended questions |
| Gureje et al. (2007)(66) | CIDI-WMH | U.S. Health Interview Survey |

Table s6. Results for pain by depression or depression by pain as frequencies and/or effect estimates

| **Authors (year)** | **Age of included participants; proportion of females** | **Prevalence of depression (%)** | **Prevalence of pain (%)** | **Results for pain by depression** | | **Results for depression by pain** | |
| --- | --- | --- | --- | --- | --- | --- | --- |
|  |  |  |  | **Comorbid frequency (%)** | **Effect estimates (95% CI)** | **Comorbid frequency (%)** | **Effect estimates (95% CI)** |
| Atlantis et al. (2011)(6) | N/R | N/R | N/R | Pain occasional: 32,  pain daily: 43 | RR: 5.36 (1.05-27.34) | N/R | N/R |
| Wong et al. (2020)(7) | 60.9 years (mean);  55.5% female | 20.9 | 64.6 | N/R | N/R | Sometimes: 18.8,  all the time: 32.1 | OR: 1.08 (1.01-1.15) |
| Costa Cabral et al. (2014)(8) | 51.4 years (mean);  69% female | 11.6 | 42 ^ꚙ^ | N/R | N/R | 62.5 | PR: 1.59 (1.33-1.90) |
| Shega et al. (2013)(9) | N/R | N/R | No or very mild pain: 64.6,  moderate or greater pain: 35.4 | N/R | N/R | No or very mild pain: 16.4,  moderate or severe pain: 36.5 | N/R |
| Tripp et al. (2006)(10) | 50.2 years (mean);  53% female | MDD: 5.9,  Other depressive disorder: 8.7 | Grade I: 34, Grade II: 26,  Grade III: 8.5,  Grade IV: 8.0;  <90 days: 51,  >90 days: 49 ^ꚙ^ | N/R | N/R | Grade III and IV: 55;  depressive symptoms: 35 | Grade III and IV:  depressive symptoms: unadjusted OR: 3.0 (1.7-5.0), adjusted OR: 1.7 (0.9 - 3.2);  major disorder: unadjusted OR: 7.0 (4.0-12), adjusted OR: 4.5 (2.2-9.3) |
| Del pilar et al. (2024)(11) | N/R;  N/R | 18.1 | 71.4 | N/R | N/R | 84.5 | Depressive symptoms:  mild and very mild pain: OR: 2.17 (1.70-2.76),  moderate pain: OR: 2.67 (2,03-3,52),  severe and extreme Pain: OR: 5.2 (4.10-6.73);  Severe depression:  Mild and very mild pain: OR: 3.55 (2.02-6.25), moderate pain: OR: 5.46 (3.01-9.91),  Severe and extreme pain: OR: 13.93 (8.10-23.94) |
| Moreno et al. (2022)(12) | 77.2 years (mean);  61.3% female | Positive screen: 22.9,  self-report: 17.9 | N/R | N/R | N/R | N/R | Positive screen: OR: 1.19 (1.02-1.38),  self-report: OR: 1.27 (1.08-1.50) |
| Chi et al. (2004)(13) | N/R;  51.2% female | 60-74 years: 11.6,  >75 years: 14.4 | 23.7 ^ꚙ^ | 22.6 | OR: 1.93 (1.18-3.16) | N/R | N/R |
| Chen et al. (2012)(14) | N/R;  60.8% female | 3.3 | Other chronic pain: 3.97,  One chronic pain: 26.33,  Two or more pain: 9.23 ^ꚙ^ | Depressive symptoms without MDD: 41.0,  MDD: 64.2 | Other chronic pain and:  depressive symptoms without MDD: unadjusted OR: 1.90 (0.93-3.88), adjusted OR: 2.22 (1.08-4.62);  MDD: unadjusted OR: 3.87 (1.92-7.78), adjusted OR: 3.50 (1.71-7.19) | N/R | N/R |
| Gong et al. (2017)(15) | 70.7 years (mean);  58.5% female | Non-empty nest: 19.0,  empty-nest: 24.1 | Non-empty nest:  mild: 45.2,  moderate: 20.5,  severe: 2.4;  Empty nest:  mild: 50.2  moderate: 16.3,  severe: 3.3 | N/R | Mild pain: OR: 1.35 (1.05-1.73),  moderate pain: OR: 2.31 (1.72-3.11),  severe pain: OR: 2.76 (1.62-4.71) | N/R | N/R |
| Liu et al. (2018)(16) | N/R;  51.9% female | N/R | 66.2 ^◊^ | N/R | N/R | N/R | OR: 1.21,  adjusted OR: 1.07 (1.02-1.13) |
| Ma et al. (2021)(17) | N/R;  50.8% female | 33.9 | Mild pain: 11.8, moderate to severe pain: 18.4 | Mild pain: 50.1, moderate to severe pain: 70.1 | N/R | N/R | OR: 6.42 (5.83-7.08) |
| Si et al. (2019)(18) | 69.0 years (mean);  69.2% female | 12.1 | Nonchronic pain: 6.2, chronic pain: 41.1 ^ꚙ^ | N/R | N/R | Nonchronic pain: 8.9,  Chronic pain: 16.8 | Chronic pain: OR: 1.72 (1.20-2.48) |
| Tian et al. (2017)(19) | N/R | 10.9 | 39.6 | N/R | OR: 1.94 (1.15-3.39) | N/R | N/R |
| Borda et al. (2016)(20) | 71.2 years (mean);  63.4% female | Moderate: 19.5,  severe: 6.2 | 46.8 | N/R | N/R | Moderate depression: 62.8,  severe depression: 80.0 | Moderate depression: OR: 1.40 (1.00-1.97),  severe depression: OR: 2.80 (1.63-4.79) |
| Arola et al. (2010)(21) | N/R,  56.1% female | Baseline:  possible: 14, probable: 8.8;  3-year follow-up:  possible: 11.6, probable: 6.3 | Baseline: 38.1;  3-year follow- up: 37.4 | 3-year follow-up:  possible: 8,  probable: 3 | Unadjusted:  possible: OR: 2.03 (1.32-3.13),  probable: OR: 2.42 (1.24-4.69);  adjusted for age and gender:  possible: 2.27 (1.46-3.51),  probable: 2.53 (1.29-4.99);  adjusted for age, gender and baseline co-morbid psychopathology:  possible: 1.60 (1.00-2.54),  probable: 1.65 (0.81-3.37) | 3-year follow up: 50 | OR: 2.47 (1.96 – 3.11),  adjusted for age and gender: OR: 2.39 (1.89-3.02),  adjusted for age, gender and baseline affective symptoms: OR: 1.99 (1.57-2.54) |
| Chou (2006)(22) | 74.2 years (mean); 57.1% female | Depression at baseline: 24.8,  depression at 2-year follow-up: 26.9 | Pain at baseline: 30.1,  pain at 2-year follow-up: 30.5 | Pain at baseline: 50.1,  pain at 2-year follow-up: 49.3 | OR: 1.54 (1.18-1.77) | Depression at baseline: 41.2,  depression at 2-year follow-up: 43.0 | OR: 1.45 (1.27-1.88) |
| Westoby et al. (2009)(23) | 66.3 years (mean);  56% female | 4.8 | N/R | N/R | N/R | N/R | OR: 1.80 (1.1-3.1),  adjusted OR: 1.79 (1.0-3.1) |
| Ahrend et al. (2024)(24) | N/R; 51% female | 8.3 | Mild pain: 32.1,  moderate pain: 15.3,  severe pain: 11.5 | N/R | N/R | Mild pain: 6.5,  moderate pain: 14.4,  severe pain: 27.1 | Mild pain: OR: 1.8 (1.3-2.4),  moderate pain: OR: 2.8 (2.0-4.0),  severe pain: OR: 4.0 (2.8-5.6) |
| Bauer et al. (2016)(25) | 77.6 years (mean);  50.4% female | 15.9 | Chronic local pain: 57.5,  chronic widespread pain: 12.3 ^ꚙ^ | N/R | N/R | Chronic local pain: 15.1,  chronic widespread pain: 40.5 | Adjusted for age and sex:  chronic local pain: OR: 1.85 (1.02-3.35),  chronic widespread pain: OR: 6.91 (3.45-13.82);  adjusted for age, sex, social factors and psychological/physical comorbidities:  chronic local pain: OR: 1.36 (0.72-2.54),  chronic widespread pain: OR: 4.08 (1.90-8.74) |
| Denkinger et al. (2014)(26) | 76 years (mean);  N/R | N/R | N/R | Pain severity: 4.9;  multisite pain: 4.3;  chronicity:  days: 1.8,  weeks: 8.8,  months: 17.5,  years: 71.9;  quality:  breakthrough: 70.8,  continuous: 12.9,  both: 16.4;  frequency (last seven days):  never/rarely: 23.9,  sometimes/often: 46.2.  very often/ Always: 29.8 | Adjusted for age and sex:  pain severity: OR: 1.12 (1.01-1.23),  number of painful body areas: OR: 1.20 (1.11-1.31),  chronicity: OR: 1.00 (0.78-1.29),  frequency: OR: 1.18 (1.01-1.37),  quality: OR:  breakthrough: 1.08 (0.64-1.80),  continuous and breakthrough: 0.81 (0.42-1.59);  adjusted for age, sex, comorbidity and social network:  pain severity: OR: 1.12 (1.02-1.24),  number of painful body areas: OR: 1.10 (1.00-1.20),  chronicity: OR: 0.98 (0.75-1.27),  frequency: OR: 1.13 (0.97-1.32),  quality: OR:  breakthrough: 0.97 (0.57-1.65),  continuous and breaktrough: 0.68 (0.34-1.37) | N/R | N/R |
| Häuser et al. (2013)(27) | N/R;  53.4% female | N/R | 32.9 ^ꚙ^ | N/R | N/R | Local pain: 4.1,  oligolocal pain: 9.4,  multilocal pain: 28.8 | N/R |
| Pengpid & Peltzer (2021)(28) | N/R;  58.0% female | 7.6 | 12.5 | 14.4 | Unadjusted OR: 2.35 (2.06-2.69),  adjusted OR: 1.94 (1.70-2.22) | N/R | N/R |
| Regan et al. (2013)(29) | 64 years (mean);  52% female | 10 | N/R | 51.3 | OR: 3.69 (3.17-4.30) | N/R | N/R |
| Goral et al. (2010)(30) | 46.1 years (mean);  51% female | 4.7 | 29.9 ^ꚙ^ | N/R | N/R | 11.6 | OR: 2.11 (1.64-2.71) |
| Yunus et al. (2018)(31) | N/R;  60.8% female | N/R | 20.4 ^ꚙ^ | N/R | N/R | N/R | RR: 1.06 (1.03-1.09) |
| Garcia-Perez et al. (2022)(32) | 64.7 years (mean);  57.2% female | 29.8 | Frequent: 39.9,  severe: 9.3 | Low depressive symptoms:  mild/moderate pain: 57.6,  severe pain: 38.1;  high depressive symptoms:  mild/moderate pain: 42.4,  severe pain: 61.9 | Mild/moderate pain: OR: 2.40 (2.20-2.62),  severe pain: OR: 4.53 (3.97-5.16) | N/R | N/R |
| Gutierrez et al. (2022)(33) | 69.1 years (mean);  55% female | 32.1 | 39.3 | N/R | OR: 1.57 (1.32-1.87) | N/R | OR: 1.65 (1.41-1.93) |
| Geerlings et al. (2001)(34) | N/R;  57.7% female | 50.2 | Some symptoms of pain: 15.2,  many symptoms of pain: 11.3 | 87 | Some symptoms of pain: OR: 1.42 (1.15-1.77),  many symptoms of pain: OR: 1.72 (1.32-2.24) | 61 | N/R |
| Hilderink et al. (2012)(35) | 68.8 years (mean);  N/R | 22.7 | 24.4 | N/R | HR: 1.02 (1.01-1.04) | N/R | HR: 1.13 (1.05-1.22) |
| Gureje et al. (2009)(36) | N/R;  N/R | Lifetime: 3.1,  12-months: 1.1 | N/R | N/R | OR: 2.5 (1.4-4.7) | N/R | N/R |
| Satghare et al. (2016)(37) | N/R;  N/R | N/R | 19.5 | N/R | N/R | 37.6 | OR: 2.8 (2-4) |
| Zager et al. (2023)(38) | 49.2 years (mean);  50.4% female | N/R | Severe: 9.2 | Severe pain: 6.8 | N/R | N/R | N/R |
| Dueñas et al. (2024)(39) | N/R;  N/R | N/R | Non-chronic continuous pain: 7.7,  chronic pain: 25.9;  pain intensity:  very little/little: 4.7, moderate: 33.3, great: 57.6, unbearable: 4.4 ^ꚙ^ | N/R | N/R | Non-chronic continuous pain: 14.2, chronic pain: 22.2 | N/R |
| Rivera et al. (2019)(40) | 42.8 years (mean);  50.9% female | 6.5 | N/R | Pain conditions: 43.1,  chronic pain: 32.2 | Unadjusted OR: 2.77 (2.17-3.52),  adjusted OR: 2.25 (1.75-2.89) | N/R | N/R |
| Sendra-Gutierrez et al. (2017)(41) | 75.9 years (mean);  59.8% female | MDD: 6.2,  Other depressive disorders: 6.4 | Men: very slight: 11.0, slight: 15.1, moderate: 15.4, severe: 7.5, extreme: 1.2; Women:  very slight: 9.4,  slight: 16.3,  moderate: 27.5,  severe: 16.7,  extreme: 2.4 | Men: very slight: 2.8,  slight: 4.7,  moderate: 11.2,  severe: 35.6,  extreme: 60.0;  Women:  very slight: 8.5,  slight: 11.8,  moderate: 18.5,  severe: 36.7,  extreme: 63.4 | Men: OR:  very slight: 0.52 (0.19-1.42), slight: 1.01 (0.52-2.00), moderate: 1.85 (1.04-3.29), severe: 3.45 (1.87-6.37), extreme: 5.02 (1.79-14.10);  Women: OR:  very slight: 1.05 (0.59-1. 85), slight: 1.14 (0.73-1.78), moderate: 1.27 (0.86-1.88), severe: 2.02 (1.33-3.07), extreme: 3.85 (2.02-7.35) | N/R | N/R |
| Thomtén et al. (2012)(42) | N/R;  100% female | N/R | Baseline: 63.8,  follow-up: 61.3 | N/R | Pain at baseline and follow-up:  OR: 6.45 (5.23-7.95) | N/R | N/R |
| Rouch et al. (2023)(43) | N/R; N/R | N/R | N/R | N/R | Adjusted for pain and lifetime depression:  current: OR: 1.76 (1.15-2.71), remitted: OR: 1.33 (1.08-1.63);  adjusted for pain, lifetime depression and early life traumatic events:  current: OR: 1.73 (1.13-2.67), remitted: 1.31 (1.07-1.62);  adjusted for pain, lifetime depression, early-life traumatic events and personality scores:  current: OR: 1.52 (0.98-2.38), remitted: OR: 1.22 (0.99-1.52) | 51.8 | N/R |
| Ko et al. (2018)(44) | 74.5 years (mean);  53.1% female | 5.4 | 20.9 | 37.1 | N/R | N/R | N/R |
| Erbaydar & Çilingiroğlu (2010)(45) | 39.7 years (mean); 72.2% female | N/R | Acute: 18.1,  chronic: 50.6 ^ꚙ^ | N/R | N/R | 38.2 | N/R |
| Bollinger et al. (2020)(46) | N/R;  50.6% female | 39.2 | 32.2 ^ꚙ^ | N/R | N/R | 50.2 | OR: 1.67 (1.04 – 2.67) |
| Emptage et al. (2005)(47) | 57 years (mean);  N/R | 8.1 | Mild/moderate pain: 15.5,  severe pain: 2 | 53.1 | N/R | N/R | N/R |
| Janevic et al. (2017)(48) | N/R;  N/R | N/R | 8.2 ^ꚙ^ | N/R | N/R | N/R | OR: 3.30 (2.23-4.87),  adjusted OR: 1.92 (1.17-3.17) |
| McCarthy et al. (2009)(49) | 80 years (average);  62.8% female | N/R | Any pain: 74.6,  chronic pain: 52.0 ^ꚙ^ | N/R | N/R | Chronic pain: 11.0 | Univariate logistic regression: OR: 2.48 (1.40-4.55),  multivariate logistic regression: OR: 1.94 (1.03-3.66) |
| Miller & Cano (2009)(50) | 51.1 years (mean);  62.1% female | 4.6 | 21.9 ^ꚙ^ | N/R | N/R | 35.1 | N/R |
| Mullins et al. (2023)(51) | 47.8 years (mean);  51.6% female | None-minimal: 81.5, mild: 11.6, moderate: 4.2,  severe: 2.8 | 79.5 ^ꚙ^ | N/R | N/R | None-minimal: 57.6, mild: 22.3,  moderate: 11.4,  severe: 8.7 | N/R |
| Ohayon & Schatzberg  (2010)(52) | N/R;  51.2% female | 6.3 | 49 ^ꚙ^ | 66.3 | N/R | N/R | OR: 1.8 (1.1-2.8) |
| Patel et al. (2013)(53) | N/R; N/R | N/R | 52.9 | N/R | N/R | 68.9 | N/R |
| Shega et al. (2014)(54) | N/R; N/R | N/R | Pain presence: 66.9 | N/R | N/R | None to mild: 3.3,  moderate or greater: 5.0 | N/R |
| Shi et al. (2010)(55) | N/R; N/R | N/R | 34.1 | N/R | N/R | N/R | Unadjusted OR: 2.05 (1.87-2.25),  adjusted OR: 1.70 (1.51-1-91) |
| Steffens et al. (2009)(56) | N/R;  60% female | 11.19 | N/R | N/R | OR: 2.46 (1.33-4.53) | N/R | N/R |
| Tian et al. (2005)(57) | 59.8 years (mean);  56% female | 5.7 | Mild/moderate: 14.5, severe: 1.5 | 51 | N/R | N/R | N/R |
| Uebelacker et al. (2014)(58) | N/R;  100% female | N/R | N/R | N/R | Baseline:  unadjusted and adjusted OR: 0.58 (0.57-0.59);  3-year follow-up: unadjusted OR: 0.68 (0.67-0.70), adjusted OR: 0.69 (0.67-0.70) | N/R | N/R |
| Qiu et al. (2022)(59) | CHARLS:  57.8 years (mean), 48.7% female; ELSA:  63.2 years (mean) 54.4% female | CHARLS: baseline: 16.9,  follow-up: 22.4; ELSA: baseline: 11.1,  follow-up: 25.7 | CHARLS:  mild to moderate: 15.3, severe: 6.7;  ELSA:  mild to moderate: 19.5,  severe: 7.7 | CHARLS: mild to moderate: 32.1, severe: 36.0;  ELSA: mild to moderate: 34.9, severe: 45.8 | CHARLS: mild to moderate: HR: 1.29 (1.16-1.44), severe: HR: 1.42 (1.23-1.64);  ELSA: mild to moderate: HR: 1.46 (1.32-1.62), severe: HR: 1.62 (1.41-1.85);  Meta-analysis: mild to moderate: pooled HR: 1.37 (1.22-1.55);  severe: pooled HR: 1.52 (1.34-1.73) | CHARLS: 55.0;  ELSA: 55.8 | CHARLS: HR: 1.74 (1.61-1.89);  ELSA: HR: 1.65 (1.48-1.84);  Meta-analysis:  pooled HR: 1.71 (1.60-1.82) |
| Langley (2011)(60) | N/R;  N/R | N/R | Moderate pain: 12.0, severe pain: 4.61, daily pain: 8.85 | N/R | N/R | Mild pain: 19.74, moderate pain: 22.97, severe pain: 35.38, total pain population: 25.21 | N/R |
| Ohayon & Schatzberg (2003)(61) with companion report Ohayon (2004)(62) | N/R;  52% female | Ohayon & Schatzberg (2003): 16.5;  Ohayon (2004): 4.0 | Ohayon & Schatzberg (2003):  ≥1 chronic painful physical condition: 17.1 ^ꚙ^;  any pain:  women: 20.7,  men: 13.2;  Ohayon (2004):  ≥1 chronic painful physical condition: 17.1 ^ꚙ^ | Ohayon & Schatzberg (2003):  1/3 key symptoms for depression and ≥1 painful physical condition: 27.6,  >2 symptoms for depression and ≥1 painful physical condition: 28.5,  5 symptoms for depression and ≥1 painful physical condition: 37.9,  8 symptoms for depression and ≥1 painful physical condition: 61.9;  Ohayon (2004): 43.4 | Ohayon & Schatzberg (2003):  chronic painful physical condition: unadjusted OR: 4.39 (3.65-5.28), adjusted OR: 3.57 (2.91-4.38);  pain present day and night: unadjusted OR: 3.56 (2.94-4.30), adjusted OR: 1.6 (1.3-2.0);  Ohayon (2004): 4.0 (3.5-4.7 | N/R | N/R |
| Horackova et al. (2018)(63) | 74 years (mean);  53% female | 29 | 48 | 68 | OR: 1.97 (1.85-2.09) | N/R | N/R |
| Ogliari et al. (2023)(64) | 65.4 years (mean); 50.4% female | Follow-up: 13.6 | Mild pain: 9.8,  moderate pain: 18.4,  severe pain: 5.0 | N/R | N/R | Depression (follow-up) by pain (baseline):  mild pain: 15.0,  moderate pain: 20.1,  severe pain: 25.2 | Pain (baseline) and depression (follow-up):  mild pain: OR: 1.14 (1.01-1.28),  moderate pain: OR: 1.22 (1.10-1.34),  severe pain: OR: 1.22 (1.04-1.42) |
| Raggi et al. (2020)(65) | 60.1 years (mean);  53.3% female | Baseline: 15.2,  follow-up: 18.1 | 39.5 | Baseline: 22.3,  follow-up: 29.8 | Baseline: OR: 2.09 (1.91-2.30),  follow-up: OR: 3.27 (3.00-3.57) | N/R | N/R |
| Gureje et al. (2007)(66) | Colombia: 36.6 years (mean); 54.5% female;  Mexico: 35.2 years (mean); 52.3% female;  USA: 45.0 years (mean); 53.0% female;  Japan: 51.4 years (mean); 53.7% female;  PRC-Beijing: 39.8 years (mean), 47.5% female;  PRC-Shanghai: 42.9 years (mean), 48.1% female;  New Zealand: 44.6 years (mean), 52.2% female  Belgium: 46.9 years (mean), 51.7% female;  France: 46.3 years (mean), 52.2% female;  Germany: 48.2 years (mean); 51.7% female;  Italy: 47.7 years (mean), 52.0% female;  Netherlands: 45.0 years (mean), 50.9% female;  Spain: 45.5 years (mean), 51.4% female;  Ukraine: 46.1 years (mean), 55.1% female;  Lebanon: 40.3 years (mean), 48.1% female;  Nigeria: 35.8 years (mean); 51.0% female;  Israel: 44.4 years (mean); 51.9% female;  South Africa: 37.1 years (mean), 53.6% female | N/R | Colombia: one pain: 21.3 ^ꚙ^, ≥2 pains: 6.0 ^ꚙ^;  Mexico: one pain: 17.9 ^ꚙ^, ≥2 pains: 6.2 ^ꚙ^;  USA: one pain: 27.8 ^ꚙ^, ≥2 pains: 16.1 ^ꚙ^;  Japan: one pain: 20.2 ^ꚙ^, ≥2 pains: 7.9 ^ꚙ^;  PRC-Beijing: one pain: 23.8 ^ꚙ^, ≥2 pains: 13.2 ^ꚙ^;  PRC-Shanghai: one pain: 20.9 ^ꚙ^, ≥2 pains: 13.6 ^ꚙ^;  New Zealand: one pain: 25.9 ^ꚙ^, ≥2 pains: 13.2 ^ꚙ^;  Belgium: one pain: 25.8 ^ꚙ^, ≥2 pains: 14.6 ^ꚙ^;  France: one pain: 35.0 ^ꚙ^, ≥2 pains: 14.6 ^ꚙ^;  Germany: one pain: 22.1 ^ꚙ^, ≥2 pains: 10.4 ^ꚙ^;  Italy: one pain: 25.3 ^ꚙ^, ≥2 pains: 20.2 ^ꚙ^;  Netherlands: one pain: 20.7 ^ꚙ^, ≥2 pains: 12.5 ^ꚙ^;  Spain: one pain: 24.6 ^ꚙ^, ≥2 pains: 10.3 ^ꚙ^;  Ukraine: one pain: 26.4 ^ꚙ^, ≥2 pains: 33.9 ^ꚙ^;  Lebanon: one pain: 15.9 ^ꚙ^, ≥2 pains: 10.5 ^ꚙ^;  Nigeria: one pain: 16.8 ^ꚙ^, ≥2 pains: 13.6 ^ꚙ^;  Israel: one pain: 21.2 ^ꚙ^, ≥2 pains: 12.3 ^ꚙ^;  South Africa: one pain 25.5 ^ꚙ^, ≥2 pains: 22.8 ^ꚙ^ | N/R | N/R | Major depression:  Colombia: one pain: 8.6, ≥2 pains: 19.6;  Mexico: one pain: 8.5, ≥2 pains: 12.7;  USA: one pain: 8.5, ≥2 pains: 17.6;  Japan: one pain: 1.4, ≥2 pains: 7.1;  PRC-Beijing: one pain: 3.5, ≥2 pains: 5.6;  PRC-Shanghai: one pain: 0.5, ≥2 pains: 7.7;  New Zealand: one pain: 6.3, ≥2 pains: 12.6; Belgium: one pain: 7.1, ≥2 pains: 9.2;  France: one pain: 6.2, ≥2 pains: 11.1;  Germany: one pain: 4.2, ≥2 pains: 5.7;  Italy: one pain: 2.8, ≥2 pains: 6.6;  Netherlands: one pain: 7.8, ≥2 pains: 8.3;  Spain: one pain: 4.7, ≥2 pains: 10.6;  Ukraine: one pain: 7.1, ≥2 pains: 17.7; Lebanon: one pain: 4.3, ≥2 pains: 2.6;  Nigeria: one pain: 1.3, ≥2 pains: 2.8;  Israel: one pain: 8.4, ≥2 pains: 13.1;  South Africa: one pain: 4.8, ≥2 pains: 8.9 | One pain: pooled OR: 1.8,  ≥2 pains: pooled OR: 3.7 |
| Abbreviations: EU: European Union; HR: Hazard Ratio; MDD: Major depressive disorder; N/R: not reported; OR: Odds ratio; PRC: Peoples Republic of China; RR: Risk Ratio; UK: United Kingdom; USA: United states of America; ^ꚙ^: chronic pain; ^◊^: acute pain | | | | | | | |

Table s7. Operationalization of pain presence, duration/frequency and intensity

| **Autors; year** | **Description as reported** | **Operationalization** |
| --- | --- | --- |
| Arola et al. (2012)(21) and Westoby et al. (2009)(23) | “in the past 4 weeks have you had pain that has lasted for one day or longer in any part of your body?” | Presence |
| Regan et al. (2013),(29) Garcia-Perez et al. (2022)(32) and Gutierrez et al. (2022)(33) | “often suffer from pain” | Presence |
| Yunus et al. (2018)(31) | “within the last six months, did you experience pain in any part of your body that was continuous every day, or almost every day, for at least three months?” | Presence |
| Mullins et al. (2023)(51) | Pain in the last 3 months on 4-point scale from “never” to “every day” | Duration/frequency |
| Bollinger et al. (2020)(46) | “In the past 3 months, how often did you have pain?” | Duration/frequency |
| Mullins et al. (2023)(51) | “Pain […] in the previous 3 months: how often […] “never” to “every day” | Duration/frequency |
| Emptage et al. (2005),(47) Regan et al (2013),(29) Ogliari et al. (2023),(64) Tian (2005),(57) Langley (2011)(60) and Chou (2006)(22) | Intensity on 3-point scale from “mild” to “severe” | Intensity |
| Sendra-Gutierrez et al. (2017),(41) Zager et al. (2023),(38) Del pilar et al. (2024),(11) Moreno et al. (2022)(12) | Intensity in the last 4 weeks on 6-point scale from “none” to “extreme” / ”severe” | Intensity |
| Garcia-Perez et al. (2022)(32) | Intensity on 4-point Likert scale from “without pain” to “severe” | Intensity |

**References (supplemental material)**

1.	Davison TE, Bhar S, Wells Y, Owen PJ, You E, Doyle C, et al. Psychological therapies for depression in older adults residing in long-term care settings. Cochrane Database Syst Rev. 2024; doi:10.1002/14651858.CD013059.pub2

2.	Beecham E, Candy B, Howard R, McCulloch R, Laddie J, Rees H, et al. Pharmacological interventions for pain in children and adolescents with life-limiting conditions. Cochrane Database Syst Rev. 2015; doi:10.1002/14651858.CD010750.pub2

3.	Ipser JC, Wilson D, Akindipe TO, Sager C, Stein DJ. Pharmacotherapy for anxiety and comorbid alcohol use disorders. Cochrane Database Syst Rev. 2015; doi:10.1002/14651858.CD007505.pub2

4.	Majewski D, Ball S, Finn J. Systematic review of the relationship between comorbidity and out-of-hospital cardiac arrest outcomes. BMJ Open. 2019; doi:10.1136/bmjopen-2019-031655

5.	Xiong J, Lipsitz O, Nasri F, Lui LMW, Gill H, Phan L, et al. Impact of COVID-19 pandemic on mental health in the general population: A systematic review. J Affect Disord. 2020; doi:10.1016/j.jad.2020.08.001

6.	Atlantis E, Grayson DA, Browning C, Sims J, Kendig H. Cardiovascular disease and death associated with depression and antidepressants in the Melbourne Longitudinal Studies on Healthy Ageing (MELSHA). Int J Geriatr Psychiatry. 2011; doi:10.1002/gps.2532

7.	Wong A, Hyde Z, Smith K, Flicker L, Atkinson D, Skeaf L, et al. Prevalence and sites of pain in remote-living older Aboriginal Australians, and associations with depressive symptoms and disability. Intern Med J. 2021; doi:10.1111/imj.14870

8.	Cabral DMC, Bracher ESB, Depintor JDP, Eluf-Neto J. Chronic pain prevalence and associated factors in a segment of the population of São Paulo City. The Journal of Pain. 2014; doi:10.1016/j.jpain.2014.07.001

9.	Shega JW, Andrew M, Kotwal A, Lau DT, Herr K, Ersek M, et al. Relationship between persistent pain and 5-year mortality: a population-based prospective cohort study. J Am Geriatr Soc. 2013; doi:10.1111/jgs.12554

10.	Tripp DA, VanDenKerkhof EG, McAlister M. Prevalence and determinants of pain and pain-related disability in urban and rural settings in southeastern Ontario. Pain Res Manag. 2006; doi:10.1155/2006/720895

11.	del Pilar Ureta Fernández M, Lera L, Albala C. Association between pain and depression in chilean older adults. Reality that requires an immediate approach. Revista Medica de Chile. 2024; doi:10.4067/s0034-98872024000100069

12.	Moreno X, Sánchez H, Huerta M, Cea X, Márquez C, Albala C. Subjective Assessments of Quality of Life Are Independently Associated with Depressive Symptoms among Older Adults Enrolled in Primary Care in Chile2022 2022.

13.	Chi I, Yip PSF, Chiu HFK, Chou KL, Chan KS, Kwan CW, et al. Prevalence of Depression and Its Correlates in Hong Kong's Chinese Older Adults. The American Journal of Geriatric Psychiatry. 2005; doi:10.1176/appi.ajgp.13.5.409

14.	Chen X, Cheng HG, Huang Y, Liu Z, Luo X. Depression symptoms and chronic pain in the community population in Beijing, China. Psychiatry Res. 2012; doi:10.1016/j.psychres.2012.04.013

15.	Gong F, Zhao D, Zhao Y, Lu S, Qian Z, Sun Y. The factors associated with geriatric depression in rural China: stratified by household structure. Psychol Health Med. 2018; doi:10.1080/13548506.2017.1400671

16.	Liu XK, Xiao SY, Zhou L, Hu M, Liu HM. Different predictors of pain severity across age and gender of a Chinese rural population: a cross-sectional survey. BMJ Open. 2018; doi:10.1136/bmjopen-2017-020938

17.	Ma Y, Xiang Q, Yan CY, Liao H, Wang J. Relationship between chronic diseases and depression: the mediating effect of pain. 2021; doi:10.1186/s12888-021-03428-3

18.	Si HX, Wang CL, Jin YR, Tian XY, Qiao XX, Liu N, et al. Prevalence, Factors, and Health Impacts of Chronic Pain Among Community-Dwelling Older Adults in China2019 2019.

19.	Tian XY, Wang CL, Qiao XX, Liu N, Dong LJ, Butler M, et al. Association between pain and frailty among Chinese community-dwelling older adults: depression as a mediator and its interaction with pain. 2018; doi:10.1097/j.pain.0000000000001105

20.	Borda MG, Acevedo González JC, David DG, Morros González E, Cano CA. [Pain in the elderly: Quality of life, functionality and associated factors. SABE Study, Bogotá, Colombia]. Rev Esp Geriatr Gerontol. 2016; doi:10.1016/j.regg.2015.07.001

21.	Arola H-M, Nicholls E, Mallen C, Thomas E. Self-reported pain interference and symptoms of anxiety and depression in community-dwelling older adults: Can a temporal relationship be determined? European Journal of Pain. 2010; doi:10.1016/j.ejpain.2010.02.012

22.	Chou K-L. Reciprocal relationship between pain and depression in older adults: Evidence from the English longitudinal study of ageing. Journal of Affective Disorders. 2007; doi:10.1016/j.jad.2006.12.013

23.	Westoby CJ, Mallen CD, Thomas E. Cognitive complaints in a general population of older adults: prevalence, association with pain and the influence of concurrent affective disorders. Eur J Pain. 2009; doi:10.1016/j.ejpain.2008.11.011

24.	Ahrend JN, Jobski K, Bantel C, Hoffmann F. Pain intensity and comorbid depressive symptoms in the general population: An analysis of the german health update study (geda 2019/2020‐ehis). European Journal of Pain. 2024; doi:10.1002/ejp.4745

25.	Bauer H, Emeny RT, Baumert J, Ladwig KH. Resilience as a moderator of the association between chronic pain and depressive symptoms in the elderly: Results from the KORA-Age study. Psychosomatic Medicine. 2015; doi:10.1002/ejp.850

26.	Denkinger MD, Lukas A, Nikolaus T, Peter R, Franke S. Multisite pain, pain frequency and pain severity are associated with depression in older adults: results from the ActiFE Ulm study. Age Ageing. 2014; doi:10.1093/ageing/afu013

27.	Häuser W, Schmutzer G, Hinz A, Hilbert A, Brähler E. [Prevalence of chronic pain in Germany. A representative survey of the general population]. Schmerz. 2013; doi:doi:10.1007/s00482-012-1280-z

28.	Pengpid S, Peltzer K. Prevalence and correlates of major depressive disorder among a national sample of middle-aged and older adults in india. Aging & Mental Health. 2022; doi:10.1080/13607863.2021.2024796

29.	Regan CO, Kearney PM, Savva GM, Cronin H, Kenny RA. Age and sex differences in prevalence and clinical correlates of depression: first results from the Irish Longitudinal Study on Ageing. Int J Geriatr Psychiatry. 2013; doi:10.1002/gps.3955

30.	Goral A, Lipsitz JD, Gross R. The relationship of chronic pain with and without comorbid psychiatric disorder to sleep disturbance and health care utilization: results from the Israel National Health Survey. J Psychosom Res. 2010; doi:10.1016/j.jpsychores.2010.05.012

31.	Yunus RM, Hairi NN, Choo WY, Tan MP, Hairi F, Sooryanarayana R, et al. Elder Abuse and Chronic Pain: Cross-Sectional and Longitudinal Results from the Preventing Elder Abuse and Neglect Initiative. J Am Geriatr Soc. 2018; doi:10.1111/jgs.15370

32.	García-Pérez A, González-Aragón Pineda AE, Sandoval-Bonilla BA, Cruz-Hervert LP. Prevalence and factors associated with the depressive symptoms in rural and urban Mexican older adults: evidence from the Mexican Health and Aging Study 2018. Salud Publica Mex. 2022; doi:10.21149/13340

33.	Gutierrez S, Wong R, Milani SA. The pain and depressive symptoms cascade: A bidirectional analysis of the Mexican Health and Aging Study 2012-2015. 2022; doi:10.1002/gps.5812

34.	Geerlings SW, Twisk JWR, Beekman ATF, Deeg DJH, van Tilburg W. Longitudinal relationship between pain and depression in older adults: sex, age and physical disability. 2002; doi:10.1007/s127-002-8210-2

35.	Hilderink PH, Burger H, Deeg DJ, Beekman AT, Oude Voshaar RC. The temporal relation between pain and depression: results from the longitudinal aging study Amsterdam. Psychosom Med. 2012; doi:10.1097/PSY.0b013e3182733fdd

36.	Gureje O, Uwakwe R, Oladeji B, Makanjuola VO, Esan O. Depression in adult Nigerians: results from the Nigerian Survey of Mental Health and Well-being. J Affect Disord. 2010; doi:10.1016/j.jad.2009.04.030

37.	Satghare P, Chong SA, Vaingankar J, Picco L, Abdin E, Chua BY, et al. Prevalence and correlates of pain in people aged 60 years and above in Singapore: Results from the wise study. Pain Research and Management. 2016; doi:10.1155/2016/7852397

38.	Zager Kocjan G, Avsec A, Kavčič T. Feeling too low to be active: Physical inactivity mediates the relationship between mental and physical health. Soc Sci Med. 2024; doi:10.1016/j.socscimed.2023.116546

39.	Dueñas M, De Sola H, Salazar A, Esquivia A, Rubio S, Failde I. Prevalence and epidemiological characteristics of chronic pain in the spanish population Results from the pain barometer. European Journal of Pain. 2024; doi:10.1002/ejp.4705

40.	Rivera M, Porras-Segovia A, Rovira P, Molina E, Gutiérrez B, Cervilla J. Associations of major depressive disorder with chronic physical conditions, obesity and medication use: Results from the PISMA-ep study. Eur Psychiatry. 2019; doi:10.1016/j.eurpsy.2019.04.008

41.	Sendra-Gutiérrez JM, Asensio-Moreno I, Vargas-Aragón ML. Characteristics and factors associated with depression in the elderly in Spain from a gender perspective. Actas Esp Psiquiatr. 2017;

42.	Thomtén J, Soares JJF, Sundin Ö. Pain among women: Associations with socio-economic factors over time and the mediating role of depressive symptoms. Scand J Pain. 2012; doi:10.1016/j.sjpain.2011.12.003

43.	Rouch I, Strippoli MPF, Dorey JM, Ranjbar S, Laurent B, Von Gunten A, et al. Psychiatric disorders, personality traits, and childhood traumatic events predicting incidence and persistence of chronic pain: Results from the CoLaus|PsyCoLaus study. Pain. 2023; doi:10.1097/j.pain.0000000000002912

44.	Ko KT, Yu SH, Lee CY, Tseng HY, Chiu YF, Hsiung CA. Moderating effect of depression on the association between pain and activities of daily living in older adults. Psychogeriatrics. 2018; doi:10.1111/psyg.12332

45.	Erbaydar NP, Cilingroglu N. Chronic pain and depression: A descriptive survey among adult primary health care centre patients2010 2010.

46.	Bollinger MJ, Hudson TJ, Hu B, Han X, Long CR, McElfish P. The relationship between sociodemographic, behavioral, and clinical variables and pain in the Native Hawaiian and Pacific Islander population. Asian American Journal of Psychology. 2020; doi:10.1037/aap0000173

47.	Emptage NP, Sturm R, Robinson RL. Depression and Comorbid Pain as Predictors of Disability, Employment, Insurance Status, and Health Care Costs. Psychiatric Services. 2005; doi:10.1176/appi.ps.56.4.468

48.	Janevic MR, McLaughlin SJ, Heapy AA, Thacker C, Piette JD. Racial and Socioeconomic Disparities in Disabling Chronic Pain: Findings From the Health and Retirement Study. J Pain. 2017; doi:10.1016/j.jpain.2017.07.005

49.	McCarthy LH, Bigal ME, Katz M, Derby C, Lipton RB. Chronic pain and obesity in elderly people: results from the Einstein aging study. J Am Geriatr Soc. 2009; doi:10.1111/j.1532-5415.2008.02089.x

50.	Miller L, Cano A. Comorbid chronic pain and depression: Who is at risk? Journal of Pain. 2009; doi:doi:10.1016/j.jpain.2009.01.242

51.	Mullins PM, Yong RJ, Bhattacharyya N. Associations between chronic pain, anxiety, and depression among adults in the United States. Pain Pract. 2023; doi:10.1111/papr.13220

52.	Ohayon MM, Schatzberg AF. Chronic pain and major depressive disorder in the general population. J Psychiatr Res. 2010; doi:10.1016/j.jpsychires.2009.10.013

53.	Patel KV, Guralnik JM, Dansie EJ, Turk DC. Prevalence and impact of pain among older adults in the United States: findings from the 2011 National Health and Aging Trends Study. Pain. 2013; doi:10.1016/j.pain.2013.07.029

54.	Shega JW, Tiedt AD, Grant K, Dale W. Pain measurement in the National Social Life, Health, and Aging Project: presence, intensity, and location. J Gerontol B Psychol Sci Soc Sci. 2014; doi:10.1093/geronb/gbu101

55.	Shi Y, Hooten WM, Roberts RO, Warner DO. Modifiable risk factors for incidence of pain in older adults. Pain. 2010; doi:10.1016/j.pain.2010.07.021

56.	Steffens DC, Fisher GC, Langa KM, Potter GG, Plassman BL. Prevalence of depression among older Americans: The Aging, Demographics and Memory Study. International Psychogeriatrics. 2009; doi:10.1017/S1041610209990044

57.	Tian H, Robinson RL, Sturm R. Labor market, financial, insurance and disability outcomes among near elderly Americans with depression and pain. J Ment Health Policy Econ. 2005;

58.	Uebelacker LA, Eaton CB, Weisberg R, Sands M, Williams C, Calhoun D, et al. Social support and physical activity as moderators of life stress in predicting baseline depression and change in depression over time in the Women's Health Initiative. Social Psychiatry and Psychiatric Epidemiology: The International Journal for Research in Social and Genetic Epidemiology and Mental Health Services. 2013; doi:10.1007/s00127-013-0693-z

59.	Qiu YJ, Ma YJ, Huang XB. Bidirectional Relationship Between Body Pain and Depressive Symptoms: A Pooled Analysis of Two National Aging Cohort Studies. 2022; doi:10.3389/fpsyt.2022.881779

60.	Langley PC. The prevalence, correlates and treatment of pain in the European Union. 2011; doi:10.1185/03007995.2010.542136

61.	Ohayon MM, Schatzberg AF. Using chronic pain to predict depressive morbidity in the general population. Arch Gen Psychiatry. 2003; doi:10.1001/archpsyc.60.1.39

62.	Ohayon MM. Specific characteristics of the pain/depression association in the general population. J Clin Psychiatry. 2004;

63.	Horackova K, Kopecek M, Machů V, Kagstrom A, Aarsland D, Motlova LB, et al. Prevalence of late-life depression and gap in mental health service use across European regions. Eur Psychiatry. 2019; doi:10.1016/j.eurpsy.2018.12.002

64.	Ogliari G, Ryg J, Andersen-Ranberg K, Scheel-Hincke LL, Collins JT, Cowley A, et al. Association between pain intensity and depressive symptoms in community-dwelling adults: longitudinal findings from the Survey of Health, Ageing and Retirement in Europe (SHARE). Eur Geriatr Med. 2023; doi:10.1007/s41999-023-00835-5

65.	Raggi A, Leonardi M, Mellor-Marsá B, Moneta MV, Sanchez-Niubo A, Tyrovolas S, et al. Predictors of pain in general ageing populations: results from a multi-country analysis based on ATHLOS harmonized database. 2020; doi:10.1186/s10194-020-01116-3

66.	Gureje O, Von Korff M, Kola L, Demyttenaere K, He Y, Posada-Villa J, et al. The relation between multiple pains and mental disorders: results from the World Mental Health Surveys. Pain. 2008; doi:10.1016/j.pain.2007.05.005
